# ARABIDOPSIS Bsister and SEEDSTICK MADS-box transcription factors modulate maternal nutrient flow for seed development in Arabidopsis

**DOI:** 10.1038/s41467-026-77573-2

**Published:** 2026-09-10

**Authors:** Camilla Banfi, Nicola Babolin, Chiara Astori, Chiara Mizzotti, Carlotta Nardi, Rosario Vega-Léon, Giulia Leo, Ueli Grossniklaus, Matthew R. Tucker, Fabrizio Araniti, Riccardo Aiese Cigliano, Walter Sanseverino, Ignacio Ezquer, Jose M. Muino, Kerstin Kaufmann, Maurizio Di Marzo, Lucia Colombo

**Affiliations:** 1 Dipartimento di Bioscienze, Università degli Studi di Milano, Milan, Italy; 2 Plant Cell and Molecular Biology, Humboldt-Universität zu Berlin, Institute of Biology, Berlin, Germany; 3 Department of Plant and Microbial Biology & Zurich-Basel Plant Science Center, University of Zurich, Zurich, Switzerland; 4 Waite Research Institute, School of Agriculture, Food and Wine, The University of Adelaide, Urrbrae, SA Australia; 5 Dipartimento di Scienze Agrarie e Ambientali - Produzione, Territorio, Agroenergia, Università degli Studi di Milano, Milan, Italy; 6 Sequentia Biotech, Barcelona, Spain

**Keywords:** Seed development, Plant morphogenesis

## Abstract

Successful seed development in Angiosperms depends on the coordinated transport and allocation of sugars from maternal tissues to the developing embryo and endosperm. In *Arabidopsis thaliana*, ovules function as carbohydrate sink organs, accumulating starch in both gametophytic and sporophytic domains prior to fertilization. During early embryogenesis, the stored starch is then mobilized. Despite extensive knowledge of starch metabolism in photosynthetic tissues, the regulatory mechanisms governing sugar transport in reproductive organs remain poorly understood. Recent studies have identified fertilization-dependent changes in nutrient flow, including callose-mediated modulation of symplastic transport at the phloem unloading site of developing seeds. However, the molecular players orchestrating these transitions are still largely unknown. Here, we show that the MADS-box transcription factors ABS/TT16 and STK play critical roles in regulating maternal nutrient flow during ovule maturation and seed development. We dissect their functional roles using omics and genetic approaches, underscoring the importance of different ovule tissues in coordinating sugar transport pathways for post-fertilization development. Our findings reveal a previously underappreciated layer of genetic control over nutrient allocation in reproductive tissues and provide new insights into the metabolic reprogramming required for successful seed formation.

## Abstract (web summary)

The modulation of nutrient flow to developing seeds from maternal tissues is essential for the development of the next generation. Here the authors show that MADS-box transcription factors contribute to this process by regulating sugar metabolism.

## Introduction

Angiosperm ovules are complex structures in which the haploid female gametophyte (embryo sac) is surrounded by diploid sporophytic tissues, comprising the ovule integuments, nucellus, chalaza and the funiculus^1^. Arabidopsis has bitegmic ovules; each integument is initially composed of two layers named, from inside to out, inner integument 1 (ii1), inner integument 2 (ii2), outer integument 1 (oi1), and outer integument 2 (oi2)^2^. Before fertilization, the ii1, termed endothelium, divides to form the fifth integument layer, called ii1’, which is located between the ii1 and ii2^1–3^. At ovule maturity, the integuments completely envelop the seven-celled female gametophyte containing the female gametes, the egg cell (EC) and the central cell (CC). Double fertilization of the EC and the CC marks the conversion of ovules into seeds, after which the two fertilization products, the embryo and the endosperm, grow enclosed by the maternal seed coat^4^. In mature ovules, the residual nucellus and chalaza region are located between the embryo sac and the funiculus^1^, mediating nutrient transfer from the maternal plant to the embryo sac before fertilization, and afterwards, to the next generation. Indeed, communication and solute exchange between the maternal sporophyte and the fertilization products are crucial for proper seed development^5^.

Sugars (mainly sucrose) are transported for long distances through the phloem, from the photosynthetic tissues to the chalazal region of ovules and seeds, where the vasculature ends (phloem end) and nutrient unloading occurs^6–10^. Afterwards, nutrients move from cell to cell either symplastically or apoplastically, eventually reaching the developing embryo and endosperm^8^. Symplastic transport involves the diffusion of solutes through cells via plasmodesmata (PD), whose aperture can be modulated by the deposition and breakdown of callose, a β−1,3-glucan polymer, leading to symplastic isolation or connection. Callose turnover is mediated by the antagonistic activity of Glucan Synthases (GSL/CalS) and β−1,3-Glucanases (BG), whose regulation has not been fully dissected^11^. Overexpression of *Plasmodesmata-Localized Proteins 1* (*PDLP1*) and *PDLP5* reduces symplastic connectivity by recruiting callose at plasmodesmata^12,13^. In roots, a feedback loop between the phytohormone auxin and *PDLP* expression has been described, likely mediated by Auxin Response Factors (ARFs), but whether a similar mechanism operates in ovules or seeds is unknown^14^. Recently, a fertilization-dependent mechanism regulating seed size has been identified, which depends on callose turnover at the chalazal phloem end^10^. Overexpression of the *β−1,3-Glucanase BG_PPAP* results in increased seed size, likely due to enhanced nutrient efflux through plasmodesmata, while its knock-out mutation is associated with smaller seeds^10^. The model proposes that callose starts to be deposited in the ovule chalaza before fertilization, and its removal in the seed is needed to enable symplastic nutrient transport^10^. However, it remains unclear how this mechanism is regulated, and if it plays a role in the fertilization process and early stages of seed development.

Although part of nutrient transport occurs through PDs, distinct symplastic domains are present, through which solute movement is mediated by transporters via the apoplast. Sugar transport in Arabidopsis is guided by three groups of transporters named Sugars Will Eventually be Exported Transporters (SWEET), Sucrose Transporters (SUT/SUC), and Monosaccharide Transporters (MST)^15^. In the seed, SWEET transporters specifically mark distinct compartments^16^. Concurrent mutation of multiple *SWEET* genes negatively affects embryo growth and seed size, likely due to reduced nutrient supply, but still results in viable seeds^9^, suggesting that multiple ways of sugar movement coexist. Similarly, *Sugar Transport Protein* (*STP)*, belonging to the *MST* superfamily, and *SUT/SUC* encoding genes show a certain specificity in expression in the seed coat, embryo and endosperm^9,16^.

Arabidopsis ovules and seeds behave as sink organs, in which the soluble sugars are converted into starch in both gametophytic and sporophytic tissues^17–19^. Starch is a complex branched glucose polymer that serves as a major carbohydrate source in plants, used to sustain metabolism, growth and development^20^. Starch formation in sink tissues relies on the translocation of hexose phosphate (e.g., Glucose 6-phosphate) from the cytosol into amyloplasts, mainly mediated by Glucose 6-Phosphate/Phosphate Translocators (GPT)^17,21,22^. In mature-stage ovules, the largest starch deposit is localized in the CC, whereas after fertilization the starch contained in the embryo sac is metabolized; concurrently, starch accumulates in the seed chalaza and in the persistent nucellus^17,18^. Increased starch content in Arabidopsis seeds was observed in mutants for genes involved in the starch degradation pathway, such as *GLUCAN, WATER DIKINASE* (*GWD*) and *β-AMYLASES* (*BAM*)^23^. Elevated starch accumulation was also reported in ovules and seeds of loss-of-function double mutants for *ARABIDOPSIS Bsister/TRANSPARENT TESTA 16* (*ABS/TT16*) and *SEEDSTICK* (*STK*). *abs stk* plants are additionally characterized by reduced fertility^19^. *ABS* and *STK* are sporophytically expressed MADS-box genes involved in several aspects of ovule and seed development. *ABS* regulates the differentiation and pigmentation of the endothelium, as well as nucellus degeneration^3,18,19,24–28^. In the *abs* mutant, the endothelium exhibits abnormal cell morphology, and ovules show a pronounced nucellus. After fertilization, *abs* seeds fail to accumulate proanthocyanidins (PAs) that give the typical pigmentation to the Arabidopsis seeds^27^. In contrast to *abs*, the *stk* mutant is characterized by ectopic accumulation of PAs in the seed coat^29^. Before fertilization, *STK* controls ovule identity redundantly with *SHATTERPROOF 1* (*SHP1*) and *SHP2*^30^. *STK* is also required for proper seed coat differentiation, seed size determination, and seed abscission^29–35^. Until now, a role for these two TFs in controlling sugar metabolism was not reported.

Here, we integrate RNA sequencing (RNA-seq), Chromatin Immunoprecipitation Sequencing (ChIP-seq), and metabolomic profiles to unveil *ABS* and *STK* downstream pathways. We dissect the *abs stk* phenotype, highlighting compromised development of the embryo during the early stages of seed formation, and we link this phenotype to disrupted sugar metabolism and transport. Furthermore, we propose a model in which these two TFs act upstream of the hormone signaling pathway and regulate callose turnover. Our results demonstrate that *ABS* and *STK* have pivotal importance in seed formation by modulating the multistep maternal nutrient flow in developing ovules and seeds.

## Results

### Simultaneous loss of *ABS* and *STK* function causes ovule and seed defects

**Fig. 1:**
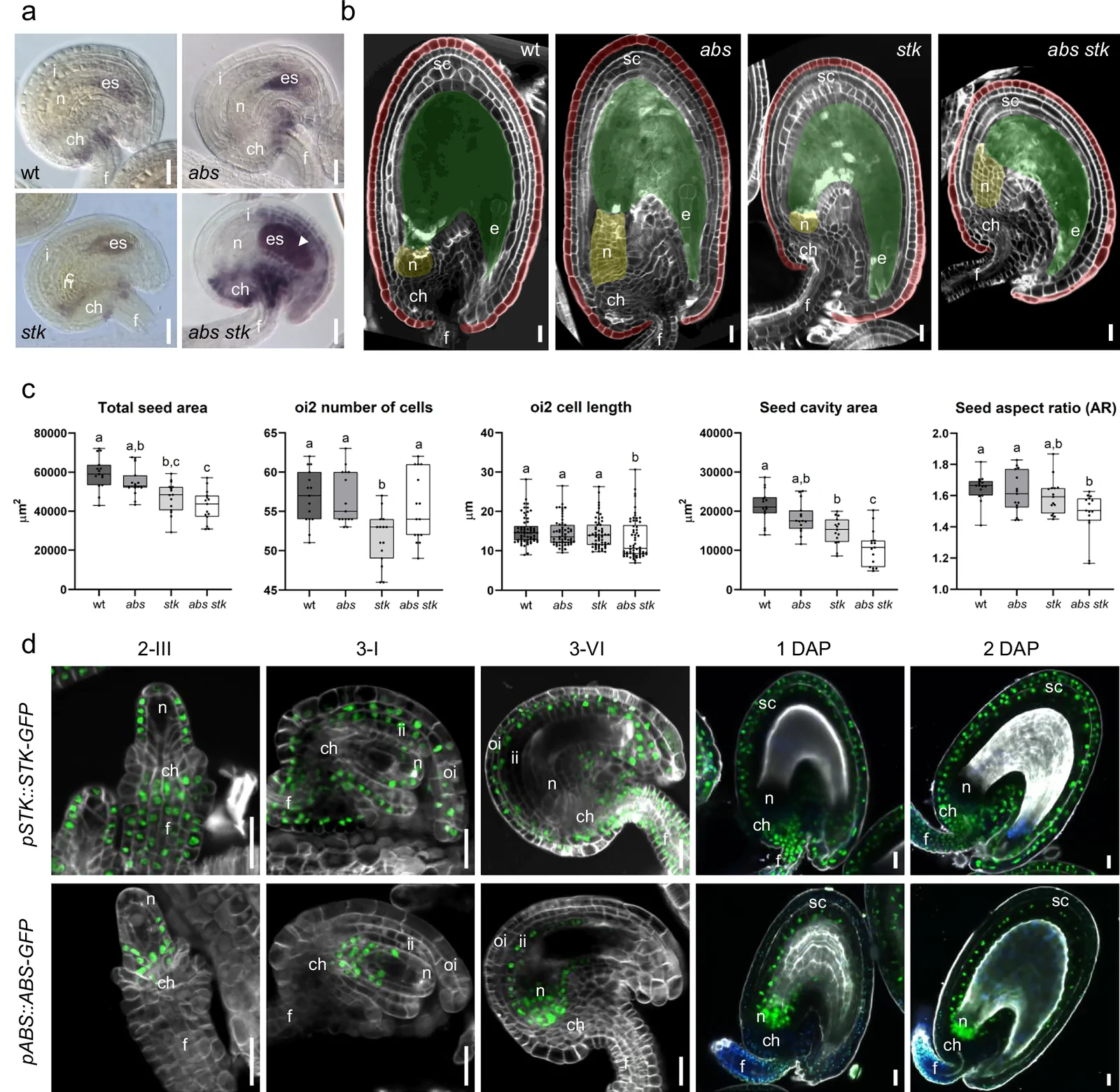
*abs stk* mutant is characterized by increased starch accumulation in ovules and defective seed development. **a** Microscopy images of wt, *abs*, *stk* and *abs stk* mature ovules stained with Lugol’s solution that marks starch accumulation (purple). The white arrowheads highlight the position of the CC in the *abs stk* mutant, where the largest starch deposit is present. **b** Confocal images of wt, *abs*, *stk* and *abs stk* seeds at 2 DAP. Seeds were stained with SR2200 (white). The colors indicate the oi2 (red), the nucellus (yellow) and the seed cavity (green). **c** Box plots showing the total seed area, the number and length of the cells composing the oi2, the seed cavity area, and the aspect ratio of wt, *abs*, *stk*, and *abs stk* seeds at 2 DAP (*n* = 15). Box plot elements correspond to: dots = single values; center line = median; box limits = 25 and 75 percentiles; whiskers = lowest and highest values. In each graph, different letters above the bars represent significant differences (*p* < 0.05) as determined by one-way ANOVA (total seed area, oi2 number of cells, seed cavity area, seed aspect ratio) or nested ANOVA (oi2 cell length) followed by Tukey’s HSD, whereas the same letters indicate absence of a statistical difference between the respective genotypes. Four independent measurements showed similar results. Source data are provided as a Source data file. **d** Expression of *pSTK::STK-GFP* and *pABS::ABS-GFP* during ovule and seed development (marked in green). Cell walls are stained with SR2200 (white) in 2-III, 3-I and 3-VI stages; in 1 DAP and 2 DAP stages, cell walls are marked in white, while chlorophyll autofluorescence is marked in blue. ch chalaza, e embryo, es embryo sac, f funiculus, i integuments, ii inner integument, n nucellus, oi outer integuments, sc seed coat. Scale bars 20 µm. Experiments were repeated on at least three independent individuals with similar results, and representative images are shown in (**a**, **b**, **d**).

The *abs stk* double mutant, rather than the single mutants, significantly impacts ovule and seed development, causing partial sterility^19^. The *abs stk* ovules are characterized by impaired ii1 periclinal division and differentiation, resulting in four-layered integuments (missing the ii1’ layer), a smaller embryo sac, and an increased ovule starch content^3,18^ (Fig. 1a). At the end of wild type (wt) ovule development, starch accumulates within the embryo sac, at the boundary between the nucellus and the chalaza, between the chalaza and the funiculus, and in the micropylar integuments (Fig. 1a). A similar pattern is observed in *abs* and *stk* single mutant ovules (Fig. 1a). By contrast, the *abs stk* ovules display ectopic starch accumulation in all these domains, with the largest starch deposit located inside the CC and around the chalaza (Fig. 1a). The starch enclosed inside the embryo sac is degraded after fertilization in wt seeds, but persists in most *abs stk* seeds^19^. At 2 Days After Pollination (DAP), *abs stk* seeds are smaller in comparison to wt and exhibit a pronounced nucellus resulting from its lack of degeneration caused by the *ABS* mutation^36^ (Fig. 1b, c). The reduced seed area of the double mutant is similar to that of the *stk* single mutant, whose seeds are also characterized by fewer oi2 cells^34^. Even if the number of oi2 cells of *abs stk* seeds is not significantly different compared to the wt, those cells have reduced length (Fig. 1c). The *abs* and *stk* mutations are additive in determining the cavity area in the double mutant seeds, which is reduced compared to both single mutants and the wt (Fig. 1b, c). The double mutant seeds are also smaller and characterized by a reduced aspect ratio, which indicates a rounder shape. *abs stk* ovule and seed phenotypes are consistent with impaired sugar metabolism, which is associated with altered starch accumulation and reduced seed size^10^. Furthermore, most of the *abs stk* seeds eventually abort at different stages, resulting in only 11% of viable seeds at maturity^19^.

To determine ABS and STK protein localization during ovule and seed development, we used functional *pSTK::STK-GFP* and *pABS::ABS-GFP* marker lines^3,29^. Both fusion proteins are localized only in the sporophytic tissues of the ovule and seed (Fig. 1d, Supplementary Fig. 1). In young ovules at stage 2-III, we detected STK-GFP in the upper nucellus, in the basal part of the chalaza, and in the funiculus. Later, at stage 3-I, the GFP signal was detected in the outer integuments and the outermost layer of the inner integuments (oi1, oi2, ii2), in the upper nucellus, until its degeneration at stage 3-VI, and in the funiculus. ABS-GFP was localized in the lower part of the nucellus (ovules stage 2-III, 3-I), in which it persisted until the mature ovule stage, and in the two innermost layers of the inner integuments (ii1 and ii1’) at stages 3-VI (Fig. 1d). In seeds until 3 Days After Pollination (DAP), STK-GFP is localized in the three outermost layers of the seed coat, in the chalaza and in the funiculus, whereas the ABS protein localization is restricted to the two innermost layers of the seed coat and in the nucellus (Fig. 1d, Supplementary Fig. 1). These data reveal striking complementarity of the expression patterns for ABS and STK, suggesting that the two proteins do not need to be localized in the same cell types to jointly regulate ovule and seed development.

### Transcriptome analysis in *abs stk* mutants highlights deregulation of genes related to sugar metabolism

**Fig. 2:**
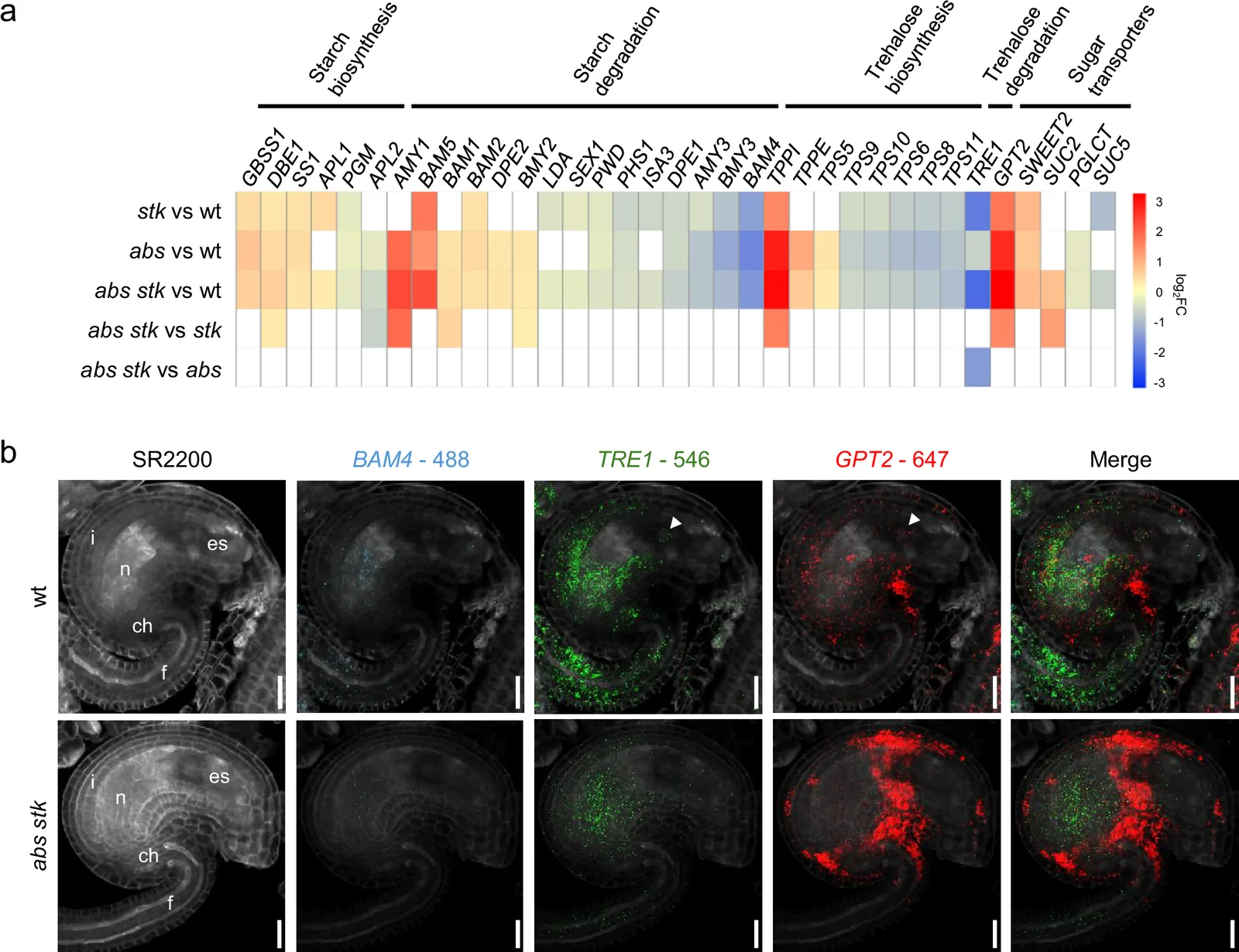
Starch and sucrose metabolic genes are deregulated in the *abs stk* double mutant before fertilization. **a** Heatmap showing the DEGs involved in starch and trehalose metabolism, and sugar transport, in the mutant-to-wt comparisons and in the double-to-single mutant comparisons derived from RNA-seq on pre-fertilization samples. FDR ≤ 0.05 was considered to identify the DEGs. *n* = 4. The genes shown in this table were filtered for the DEGs in the *abs stk* to wt comparison. Inside each category, the genes are ordered for the log_2_FC in the *abs stk* to wt comparison from the highest (red) to the lowest (blue). **b** HCR-RNA FISH showing the expression of *BAM4* (blue), *TRE1* (green) and *GPT2* (red) in wt and *abs stk* mature ovules. Ovules are counterstained with SR2200 (white) to visualize cell walls. For each genotype, the same ovule is shown as individual fluorescence channels for *BAM4*, *TRE1*, and *GPT2*, as well as in the merged image displaying all probes together. The white arrowheads indicate the probe signal in the CC. ch chalaza, es embryo sac, f funiculus, i integuments, n nucellus. Scale bars 20 µm. Experiments were repeated on three independent individuals with similar results, and representative images are shown in (**b**).

To identify the molecular networks controlled by *STK* and *ABS* in ovules before fertilization, we performed mRNA sequencing (RNA-seq) from isolated pistils of wt, *abs*, *stk*, and *abs stk* mutants (Supplementary Fig. 2a). Differential expression analysis identified differentially expressed genes (DEGs) between the wt and the three mutant backgrounds. The double and single mutants were also compared to identify genes with unique or additive expression changes (Supplementary Fig. 2b, Supplementary Dataset 1). KEGG (Kyoto Encyclopedia of Genes and Genomes) pathway enrichment analysis revealed common enriched pathways in *abs*, *stk*, and *abs stk* to wt comparisons. Among the common enriched categories, there were metabolic pathways (ath01100), biosynthesis of secondary metabolites (ath01110), carbon metabolism (ath01200), and starch and sucrose metabolism (ath00500) (Supplementary Dataset 1). These data are consistent with the previously described roles of *ABS* and *STK* in the flavonoid pathway^25,29^, and in cell wall synthesis and modification^32,33,35^. Furthermore, the enrichment of the starch and sugar metabolism term (ath00500) in the *abs stk* to wt comparison is consistent with the excess of starch in double mutant ovules. Considering the gene ontology results and the *abs stk* phenotypes, we focused our attention on genes involved in starch metabolism and sugar transport (Supplementary Table 1). In the *abs stk* to wt comparison, the most upregulated gene in starch biosynthesis was *GRANULE-BOUND STARCH SYNTHASE 1* (*GBSS1*), which encodes an enzyme required in the initial phase of starch biosynthesis (Fig. 2a)^37^. On the other hand, *β-AMYLASE 4* (*BAM4*) was the most downregulated gene involved in starch degradation^38^. Among the genes coding for the sugar transporters, *GLUCOSE 6-PHOSPHATE/PHOSPHATE TRANSLOCATOR 2 (GPT2*) was highly upregulated in *abs stk*, whereas *SUCROSE-PROTON SYMPORTER 5* (*SUC5*), encoding a sucrose transporter, was the strongest downregulated gene (Fig. 2a)^22,39,40^. Starch metabolism is reported to be influenced by trehalose, a disaccharide associated with physiological effects on growth and carbon allocation in plants^41^. Most of the genes involved in trehalose synthesis, belonging to the *TREHALOSE-6-PHOSPHATE SYNTHASE* (*TPS*) and *TREHALOSE-6-PHOSPHATE PHOSPHATASE (TPP)* gene families, were downregulated in the *abs stk* double mutant compared to wt (Fig. 2a). However, the most DEGs were *TPPI*, which was upregulated in the double mutant, and *TREHALASE 1* (*TRE1*), the only gene involved in trehalose degradation, which was downregulated^42^. All 35 genes with altered expression in *abs stk* flowers compared to wt are predicted to be expressed in mature unfertilized wt ovules according to the transcriptomic data produced by Zhang and collaborators^43^ (Supplementary Table 1). To compare the precise expression changes, at the cell-specific level, of *BAM4*, *GPT2* and *TRE1*, we performed HCR-RNA FISH, which enables the simultaneous visualization of the three transcripts on whole-mount samples (Fig. 2b). *BAM4* expression was barely detectable in most sporophytic and gametophytic tissues of wt ovules, while in the *abs stk* mutant the probe signal was confined to the nucellus. *TRE1* and *GPT2* had a similar expression profile, as they mark ovule integuments, the basal part of the nucellus, chalaza, and funiculus in wt ovules. Signal was also present in correspondence to the CC. However, *TRE1* signal remained predominantly confined to the nucellus of *abs stk* ovules. Conversely, *GPT2* was ectopically expressed in the *abs stk* micropylar integuments, surrounding the embryo sac, and around the chalazal region. Interestingly, these domains correspond to the starch accumulation area in *abs stk* mutant ovules (Fig. 1a). Similar results were obtained with digoxigenin-based in situ hybridization (Supplementary Fig. 3).

The analysis of the DEGs related to sugar and starch metabolism suggests that there is no additive effect in the double mutant with respect to the single mutants. Of the 35 DEGs between *abs stk* and wt, 26 were differentially expressed in *stk* and 29 in *abs* compared to wt, suggesting that *ABS* and *STK* act redundantly on many genes in these pathways (Fig. 2a). In conclusion, these findings indicate that *ABS* and *STK* modulate the expression of genes involved in starch and trehalose metabolism in ovules.

### STK directly regulates sugar metabolism-related genes

**Fig. 3:**
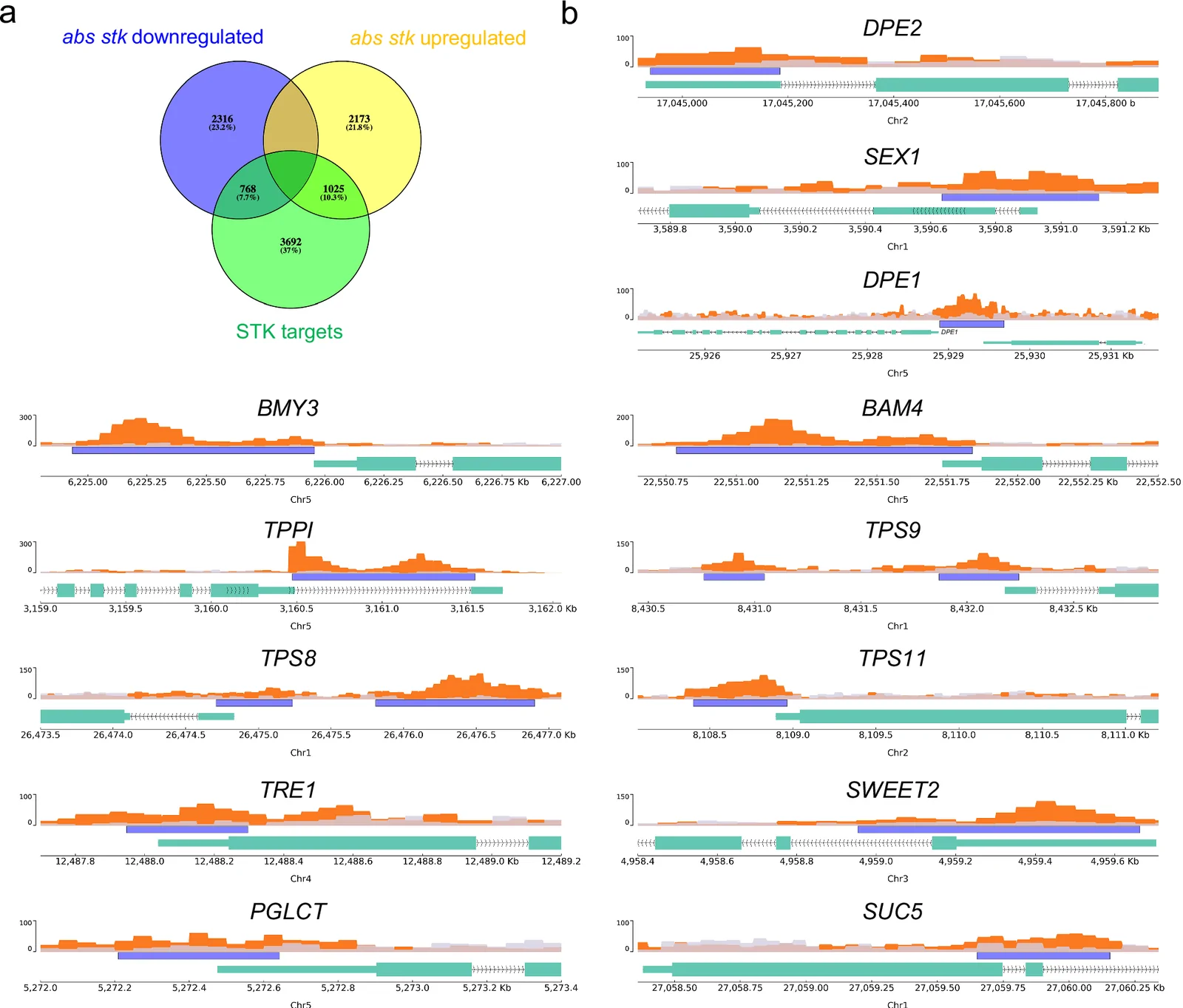
STK directly binds starch and sucrose metabolic genes. **a** Venn diagram representing the intersection between DEGs in *abs stk* vs wt comparison identified by RNA-seq in pre-fertilization samples and STK targets identified by ChIP-seq in pre-fertilization samples. **b** Visualization of STK binding sites identified by ChIP-seq in pre-fertilization samples at selected genes involved in starch and sugar metabolism (indicated in Fig. 2a). Orange tracks represent the ChIP-seq enrichment signal; gray overlay tracks indicate the background signal; blue boxes below the tracks mark the significant peak regions. Gene models are depicted with thin green boxes (utr), thick green boxes (exons), and connecting lines (introns); arrows indicate the direction of transcription.

MADS-box transcription factors regulate transcription by binding consensus sequences, CC(A/T)_6_GG, named CArG box, in the regulatory region of their target genes^44^. To gain deeper insights into the possible involvement of the MADS-box TF STK in the control of sugar metabolism genes, we performed a Chromatin immunoprecipitation sequencing (ChIP-seq) experiment using inflorescences before anthesis of *pSTK::STK-GFP* plants (Fig. 1d). We obtained 6799 significant peaks mapped to the reference genome with a total of 5485 genes that were directly bound by STK. Overall, 93% of the peaks were in the promoter region of the 5486 putative STK target genes (Supplementary Fig. 4, Supplementary Dataset 2). Functional enrichment analysis (Gene Ontology, GO) of target genes revealed that the most significant categories of the molecular functions were transcription factor activity, sequence-specific DNA binding (GO:0003700), and DNA binding (GO:0003677) (Supplementary Dataset 2). Interesting data were obtained from the analysis of the GO terms of the biological processes. In this case, the enriched categories were seed coat (GO:0010214), fruit development (GO:0010154), gynoecium development (GO:0048467), and flavonoid biosynthetic process (GO:0009813), consistent with previously reported roles of *STK*^29,33,45–47^. Other significant categories included carbohydrate metabolism, such as response to sucrose (GO:0009744), carbohydrate transport (GO:0008643), carbohydrate biosynthetic process (GO:0016051), and trehalose biosynthetic process (GO:0005992), suggesting a possible involvement of this TF in directly regulating sugar metabolism (Supplementary Dataset 2). Finally, one of the most enriched categories in the analysis of KEGG pathways was starch and sucrose metabolism (ath00500) (Supplementary Dataset 2). In particular, STK was found to bind the genomic sequences of one starch biosynthetic gene, eight genes involved in starch degradation, twelve genes involved in trehalose metabolism, and eleven genes encoding sugar transporters (Supplementary Table 1). Comparison of the ChIP-seq and RNA-seq datasets at the pre-fertilization stage revealed that only a minority of genes were both STK targets and DEGs in the *abs stk* to wt comparison (18%) (Fig. 3a, Supplementary Dataset 3). This suggests that, although STK directly regulates many genes, several of the DEGs in the *abs stk* mutant compared to wt are unlikely direct STK targets, and may be ABS targets instead. Interestingly, we identified thirteen DEGs related to the starch and sugar metabolism as direct STK targets; most of them (except *TPPI* and *SUC5*) had one or two peaks mainly localized in the promoter region (Fig. 3b). Among these were *BAM4* and *TRE1*, which showed overlapping expression with *STK* in the ovule integuments, chalaza and funiculus (Fig. 2b). The other targets were related to starch degradation (*DPE2*, *SEX1*, *DPE1*, *BMY3*), trehalose biosynthesis (*TPS9*, *TPS8*, *TPS11*) and sugar transport (*SWEET2*, *PGLCT*). Most of the STK targets were downregulated in *abs stk* compared to the wt (10/13), while only three were upregulated (Supplementary Table 1). Together, these identify STK as a direct regulator of several genes involved in sugar metabolism, mostly acting as a transcriptional activator.

### *STK* and *ABS* loss-of-function mutations impact sugar content

**Fig. 4:**
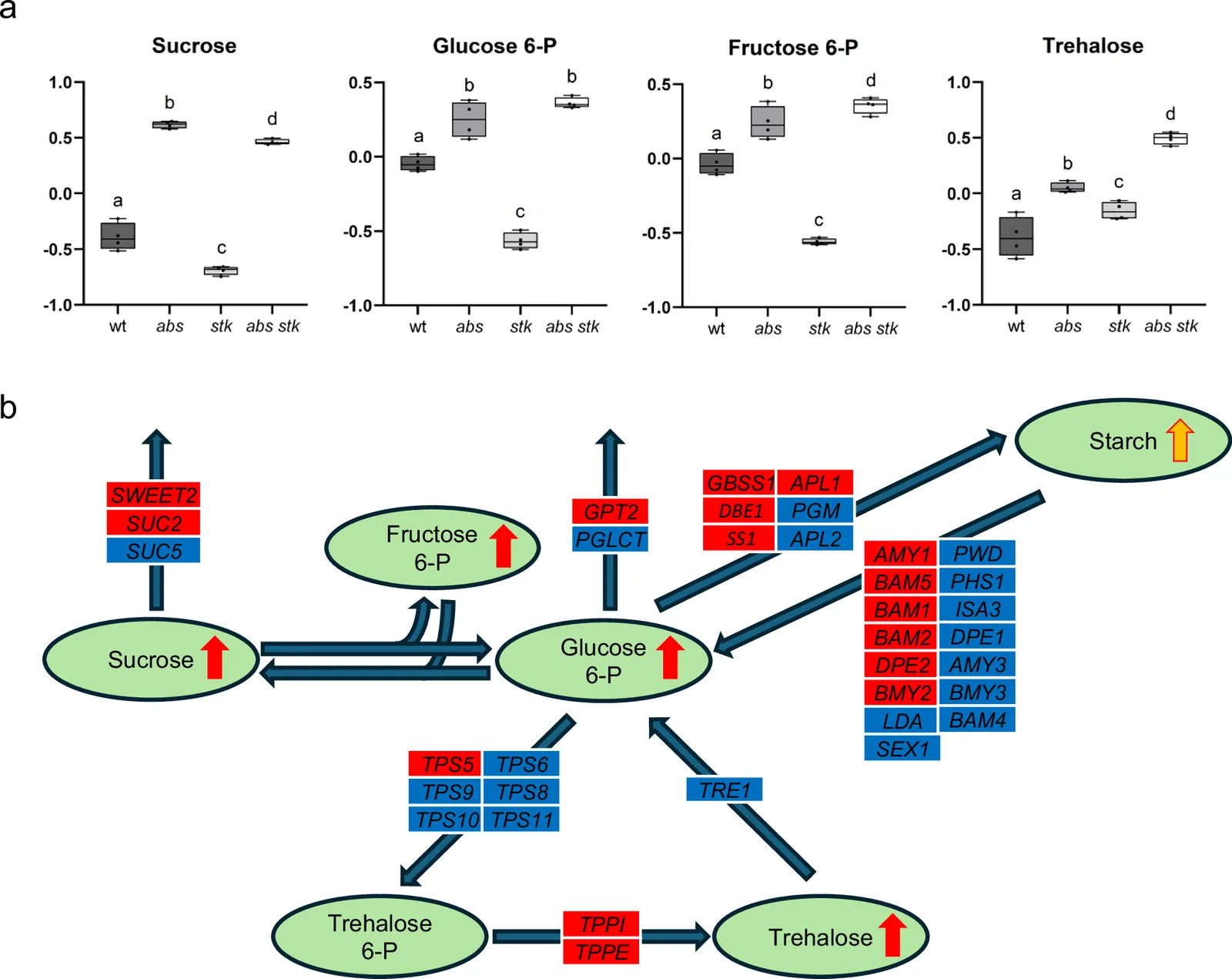
Sugar accumulation is perturbed in the abs stk mutant. **a** Box plots showing the quantification of sucrose, glucose 6-P, fructose 6-P and trehalose in wt, *abs*, *stk* and *abs stk* flowers by GC-MS. Box plot elements correspond to: dots = single values; center line = median; box limits = 25 and 75 percentiles; whiskers = lowest and highest values. In each graph, different letters above the bars represent significant differences (*p* < 0.05) as determined by univariate one-way ANOVA using the LSD test as post-hoc, whereas the same letters indicate absence of a statistical difference between the respective genotypes. *n* = 4. **b** Schematic representation of alterations in the starch and sucrose metabolic pathway in the *abs stk* to wt comparison integrating the transcriptomic and metabolomic data. The colored rectangles represent the DEGs in the *abs stk* to wt comparison involved in each step of the pathway; downregulated genes are indicated in blue, whereas upregulated genes are indicated in red. The arrows in the circles (representing the metabolites) indicate the altered quantity of the metabolite in the *abs stk* to wt comparison. Red arrows indicate sugars with a significantly higher quantity in the *abs stk* mutant compared to wt from the metabolic analysis. The yellow arrow refers to the larger starch accumulation observed by Lugol’s staining in *abs stk* ovules compared to wt, and already reported in literature^19^.

To evaluate if the differences in gene expression in *abs stk* mutants correlate with metabolic alterations, we performed a Gas Chromatography/Mass Spectrometry (GC/MS)-driven metabolomic analysis on *abs, stk*, and *abs stk* mutant flowers before anthesis. The untargeted metabolomic analysis allowed the extraction of 323 compounds, of which 135 were annotated (Supplementary Fig. 5a, b, Supplementary Dataset 4). Among them, soluble amino acids and sugars were some of the classes characterized by the highest VIP (Variable Importance in Projection) score values (Supplementary Fig. 5c). Univariate analysis revealed that the amount of 122 out of 135 metabolites significantly differed between the wt and at least one of the three mutants (Supplementary Fig. 6, Supplementary Dataset 4). Considering the *abs stk* starch phenotype and the deregulation of genes involved in starch metabolism in the double and single mutants compared to the wt, we focused attention on soluble sugars. Four of these, including sucrose, glucose 6-phosphate, fructose 6-phosphate, and trehalose, were more abundant in the *abs stk* double mutant compared to the wt and at least one of the single mutants (Fig. 4a). Sucrose is the major transport form for photo-assimilated carbon and the major source of energy for non-photosynthetic tissues like ovules and seeds^48^. We found significantly higher sucrose accumulation in the *abs* and *abs stk* mutants compared to wt, whereas *stk* showed lower sucrose accumulation (Fig. 4a). The hydrolysis of sucrose produces two important monosaccharides, glucose and fructose, which, upon phosphorylation, provide carbon for cell wall and starch biosynthesis^49^. The accumulation of glucose 6-phosphate and fructose 6-phosphate were similar to that of sucrose, with lower levels in the *stk* mutant and higher levels in *abs* and *abs stk* compared to the wt (Fig. 4a). The level of trehalose was additive in the double mutant with respect to the two single mutants, as the highest level of this compound was present in the *abs stk* double mutant, followed by *abs* and *stk* single mutants and the wt (Fig. 4a). This suggests that the two TFs may act together to limit trehalose accumulation.

By integrating the results of transcriptomic and metabolomic analyses, we generated a graphical representation of the key metabolic nodes related to the aforementioned sugars, highlighting the metabolic alterations in *abs stk* with respect to wt (Fig. 4b). The high starch content in the double mutant may not be solely attributed to elevated levels of the glucose 6-phosphate precursor, that was present at the same level in the *abs* mutant (Fig. 4a), but also from the upregulation of several genes involved in starch biosynthesis and the downregulation of genes responsible for its degradation (Fig. 4b). Similarly, increased trehalose, which was consistent with upregulation of *TPP* genes, mediating the dephosphorylation of trehalose 6-phosphate, coupled with a marked downregulation of *TRE1*, may contribute to regulate starch accumulation^41^ (Fig. 4b).

The integration of omics data suggests that the altered level of starch in *abs stk* double mutant ovules possibly derives from the mis-regulation of multiple categories of ovule-expressed genes, which cause an overall sugar balance modification.

### Modulation of starch metabolism partially rescues *abs stk* early embryo development and seed set

**Fig. 5:**
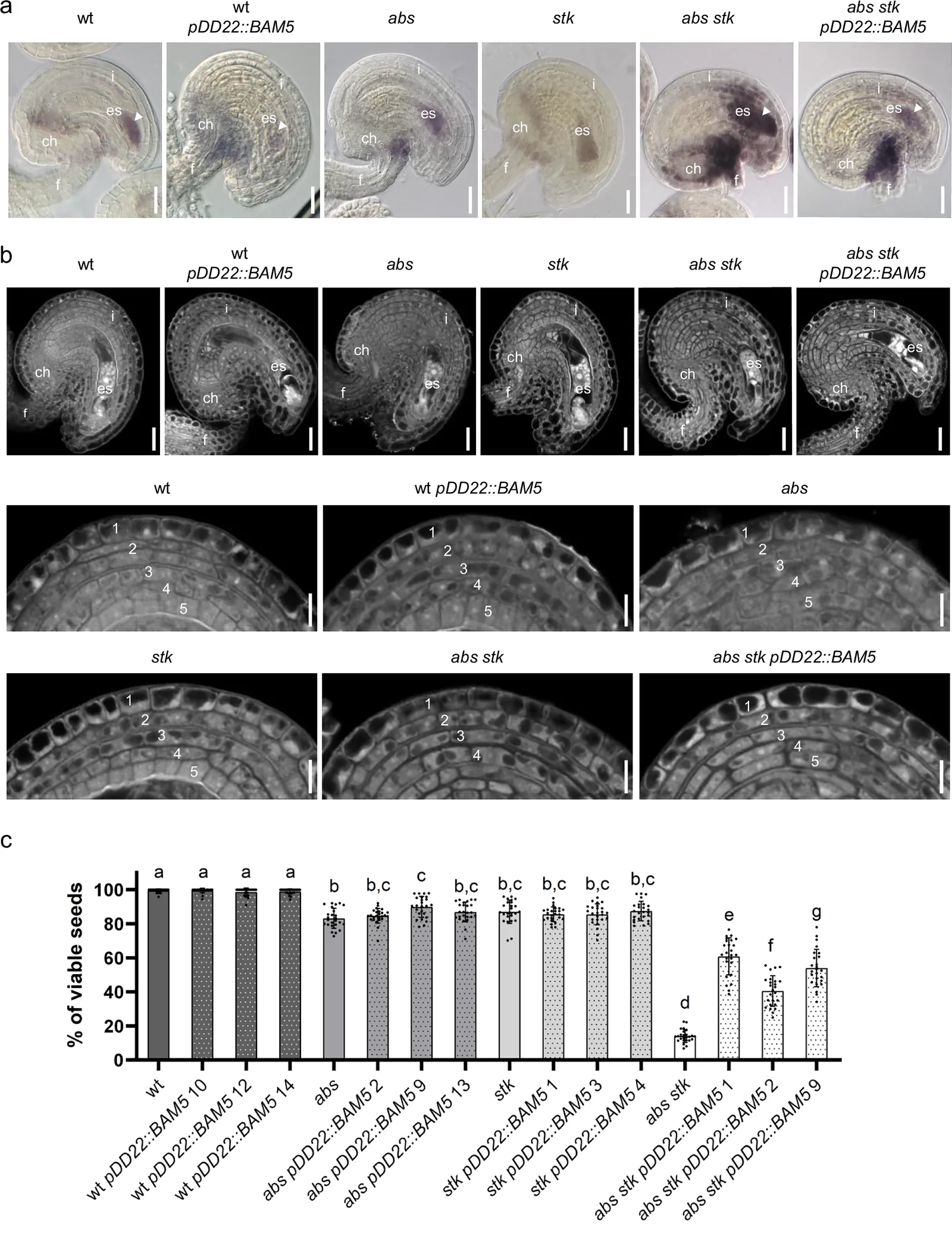
Starch mobilization in *abs stk* central cell partially rescues seed formation and integument defects. **a** Microscopy images of wt, wt *pDD22::BAM5*, *abs*, *stk*, *abs stk*, and *abs stk pDD22::BAM5* mature ovules stained with Lugol’s solution that marks starch accumulation (purple). The white arrowheads indicate the position of the CC in the *pDD22::BAM5* lines and control genotypes. **b** Confocal images of wt, wt *pDD22::BAM5*, *abs*, *stk*, *abs stk*, and *abs stk pDD22::BAM5* mature ovules, and magnification of the ovule integuments showing the number of integument layers. Ovules were stained by Feulgen staining. **c** Bar plots showing the percentage of viable seeds in siliques of wt, wt *pDD22::BAM5* 10, wt *pDD22::BAM5* 12, wt *pDD22::BAM5* 14, *abs*, *abs pDD22::BAM5* 2, *abs pDD22::BAM5* 9, *abs pDD22::BAM5* 13, *stk*, *stk pDD22::BAM5* 1, *stk pDD22::BAM5* 3, *stk pDD22::BAM5* 4, *abs stk*, *abs stk pDD22::BAM5* 1, *abs stk pDD22::BAM5* 2, and *abs stk pDD22::BAM5* 9 (*n* = 25). The graph represents the mean values ± SD, and the dots represent the single values. Different letters above the bars represent significant differences (*p* < 0.05) as determined by one-way ANOVA followed by Tukey’s HSD, whereas the same letters indicate absence of statistical difference between the respective genotypes. Measurements were repeated three times with similar results. Source data are provided as a Source data file. Ch chalaza, es embryo sac, f funiculus, i integuments. Scales bars 20 µm; magnification of the ovule integuments 10 µm. Experiments were repeated on at least three independent individuals with similar results, and representative images are shown in (**a**, **b**).

We evaluated whether the *abs stk* defects could be due to an inability to mobilize starch in the embryo sac, required for early stages of embryo development. As the largest starch deposit in *abs stk* is located in proximity to the CC^19^ (Fig. 1a), we ectopically expressed the starch-degrading enzyme BAM5 under the control of the CC-specific *pDD22* promoter^50^, where *BAM5* is not predicted to be expressed^51^. We observed that in the presence of *pDD22::BAM5* construct, both wt and *abs stk* lines showed less prominent lugol signal in the embryo sac with respect to the control conditions, suggesting reduced starch accumulation (Fig. 5a). Surprisingly, we observed a rescue of the ii1 periclinal division in the *abs stk pDD22::BAM5* line, resulting in five-layered ovules at maturity (Fig. 5b). To evaluate the percentage of developing embryos, we crossed the wt and the mutant female plants with the *pWOX9::WOX9-GFP* reporter line as pollen donor^52^. At 3 DAP, we observed a higher percentage of seeds expressing WOX9-GFP in *abs stk pDD22::BAM5* (71%) compared to *abs stk* plants (50%) (Supplementary Fig. 7a, b). Furthermore, analysis of developing seeds in the siliques of three independent *abs stk pDD22::BAM5* lines showed an increase in seed viability up to 61% compared to 14% in the *abs stk* double mutant (Fig. 5c, Supplementary Fig. 7c).

**Fig. 6:**
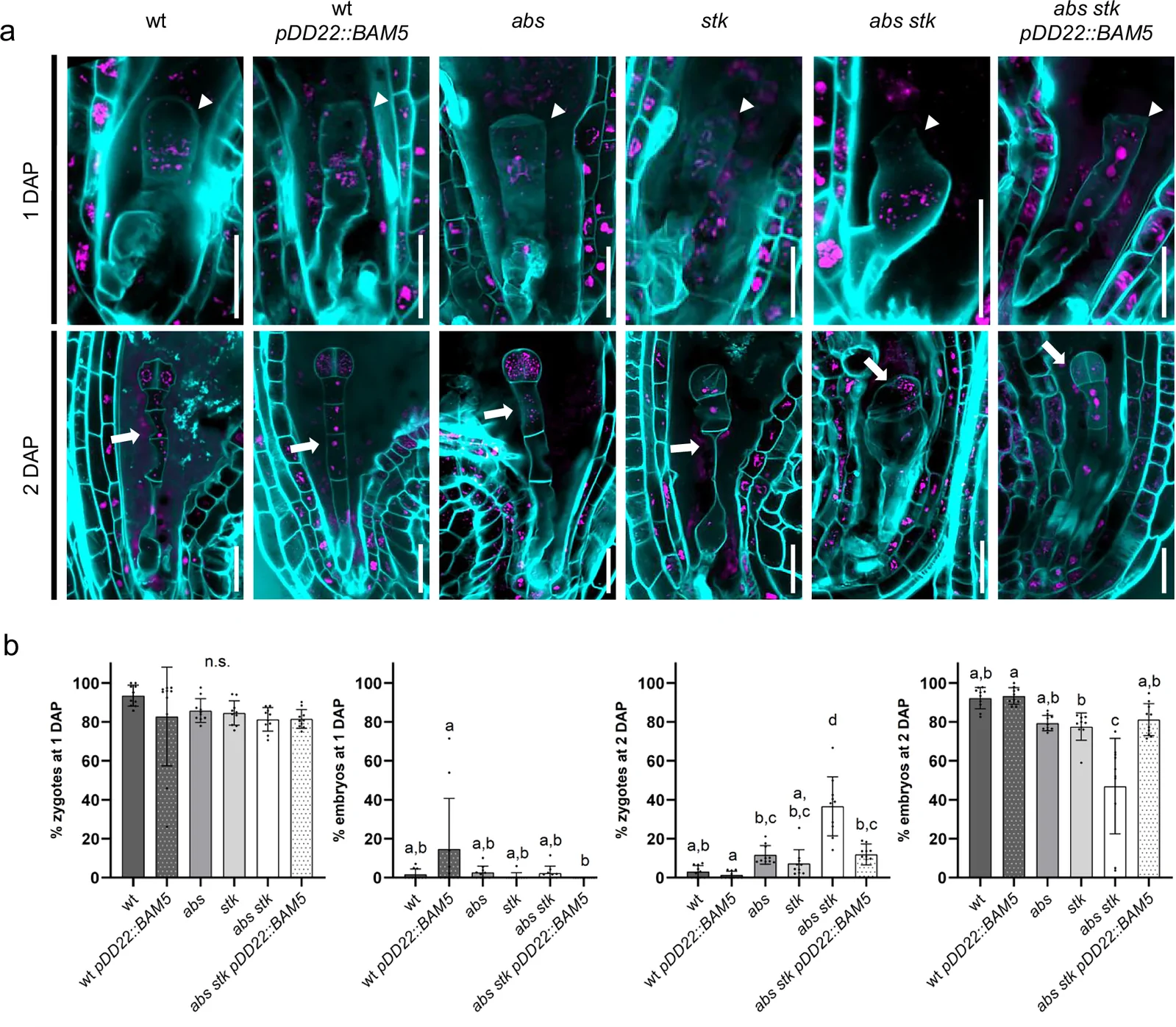
Mobilization of starch in *abs stk* central cell rescues embryo development. **a** Confocal images of wt, wt *pDD22::BAM5*, *abs*, *stk*, *abs stk*, and *abs stk pDD22::BAM5* seeds at 1 DAP and 2 DAP showing the presence of zygotes and embryos. Arrowheads point to the zygotes, while arrows point to the embryos. Seeds were stained with SR2200 (cyan) and propidium iodide (purple). Scale bars 20 µm. **b** Bar plots showing the percentage of seeds bearing zygotes and seeds bearing embryos observed at 1 and 2 DAP by confocal analysis (*n* = 10 siliques). The percentage of unfertilized ovules and aborted ovules in the same siliques is shown in Supplementary Fig. 8. The graphs represent the mean values ± SD, and the dots represent the single values. Different letters above the bars represent significant differences (*p* < 0.05) as determined by one-way ANOVA followed by Tukey’s HSD, whereas the same letters indicate absence of statistical difference between the respective genotypes. Three independent measurements showed similar results. Source data are provided as a Source data file. Experiments were repeated on at least three independent individuals with similar results, and representative images are shown in (**a**).

Since at 3 DAP the *abs stk* phenotype was not fully rescued by *pDD22::BAM5* expression, we monitored zygote and embryo formation in seeds at 1 and 2 DAP by confocal laser scanning microscopy. In wt seeds at 1 DAP, the zygote had an elongated shape before it divided by asymmetrical cell division, resulting in the embryo-proper reaching the 2/4-cell stage at 2 DAP (Fig. 6a)^53,54^. At 1 DAP, the percentage of seeds bearing zygotes was not significantly different among all the analyzed genotypes, indicating that *abs stk* is not defective in EC fertilization (Fig. 6b, Supplementary Fig. 8). However, at 2 DAP, the development of *abs stk* embryos was delayed; we observed a higher percentage of seeds still containing zygotes (37%) in comparison to all other analyzed genotypes (Fig. 6a, b). Remarkably, the *pDD22::BAM5* construct rescued early embryo development in *abs stk*, whereby the percentage of developing zygotes and embryos at 2 DAP was similar to the wt (Fig. 6b). In conclusion, in the *abs stk* mutant, zygotes likely degenerate or stop developing just after fertilization, consistent with the WOX9-GFP embryonic marker being expressed in just ~50% of the *abs stk* seeds at 3 DAP (Supplementary Fig. 7a, b).

Collectively, these data demonstrate that the simultaneous absence of *ABS* and *STK* in ovule sporophytic tissues impacts the early stages of embryo development. This seems to be caused by the altered availability or regulation of sugars required to sustain the growth of the early embryo, as embryo development could be partially restored by mobilizing the starch in the CC and fertilization-derived endosperm.

### Callose turnover is disrupted in the phloem unloading domain of *abs stk* seeds after fertilization

**Fig. 7:**
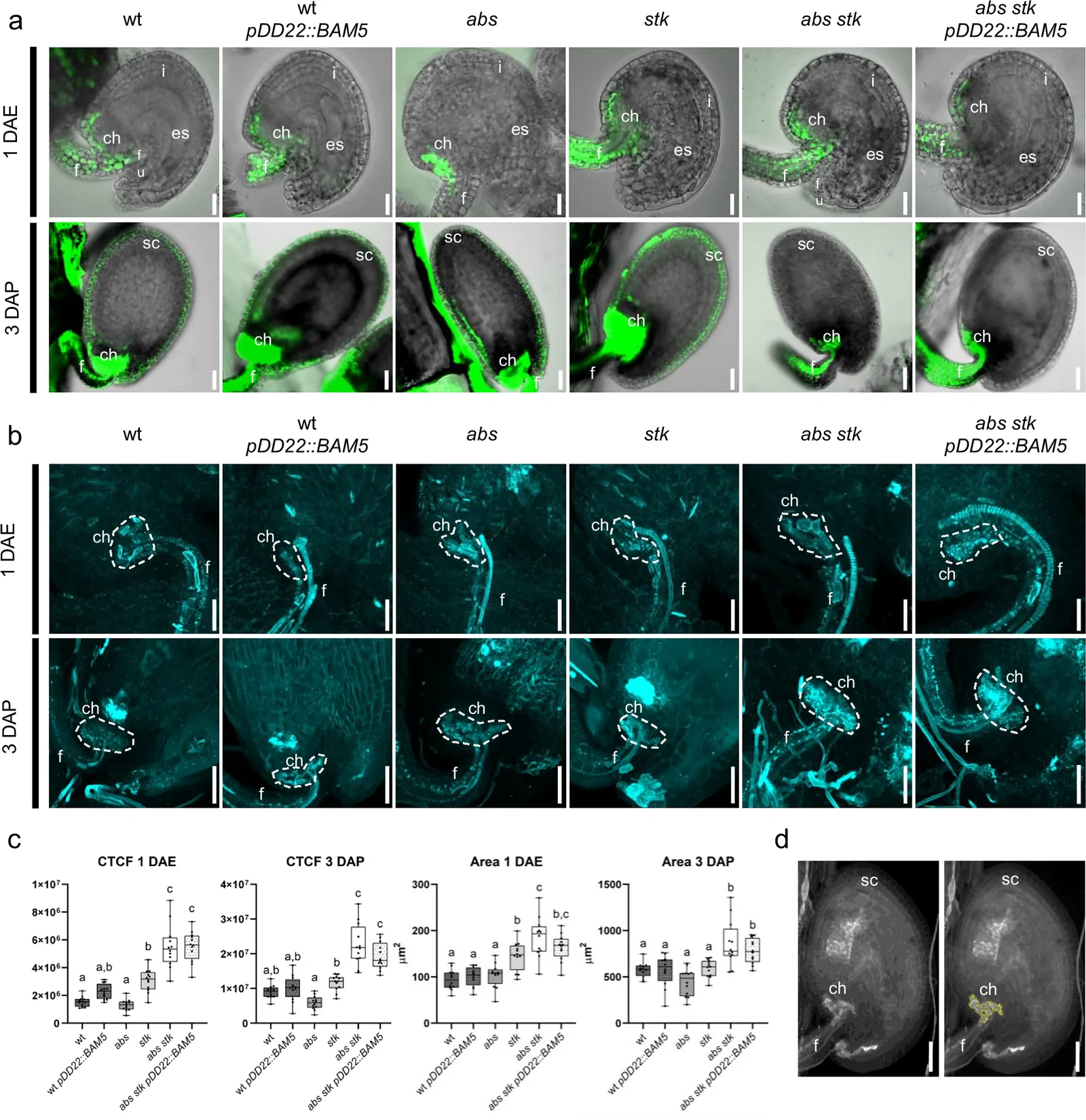
*abs stk* early seeds have defects in nutrient flow and callose deposition. **a** Confocal images of ovules at 1 DAE and seeds at 3 DAP showing CFDA symplastic tracer accumulation (green) in wt, wt *pDD22::BAM5*, *abs*, *stk*, *abs stk*, and *abs stk pDD22::BAM5* mature ovules at 1 DAE and seeds at 3 DAP. The GFP channel is merged with the brightfield channel to highlight the ovules and seeds profile. **b** 3D reconstruction of Z-stack confocal images of mature ovules at 1 DAE and developing seeds at 3 DAP stained with aniline blue (cyan), highlighting callose deposition at the chalazal phloem end (dashed line). **c** Box plots representing the Corrected Total Cell Fluorescence (CTCF) and the area of the aniline blue signal measured in the chalaza of 1 DAE ovules and 3 DAP seeds. Box plot elements correspond to: dots = single values; center line = median; box limits = 25 and 75 percentiles; whiskers = lowest and highest values. In each graph, different letters above the bars represent significant differences (*p* < 0.05) as determined by one-way ANOVA followed by Tukey’s HSD, whereas the same letters indicate absence of a statistical difference between the respective genotypes. Three independent measurements showed similar results. Source data are provided as a Source data file. **d** Example of Z-project of confocal images of *abs stk* 3 DAP seeds stained with aniline blue (white) that have been used for the quantification analysis in **c**. The area occupied by aniline blue signal (Region of Interest: ROI) is highlighted in yellow. Representative images for the other genotypes and stages are shown in Supplementary Fig. 9. ch chalaza, es embryo sac, f funiculus, i integuments, sc seed coat. Scale bar 20 µm (ovules 1 DAE) and 50 µm (seeds 3 DAP). Experiments were repeated on at least three independent individuals with similar results, and representative images are shown in (**a**, **b**, **d**).

Although the *abs stk* early embryo phenotype was partially rescued by mobilizing the starch inside the female gametophyte, in the *abs stk pDD22::BAM5* rescue lines the seed set was still significantly lower in comparison to the wt (Fig. 5c). One possible explanation for this difference is that ectopic starch breakdown in the CC increases free sugars to sustain the early stages of zygote growth and division, but additional sporophytic defects might still be present in the *abs stk pDD22::BAM5* lines. Since sugars typically move from the sporophytic tissue to the embryo sac, we analyzed the nutrient flow during early seed development by using the 5(6)-Carboxyfluorescein diacetate (CFDA), a fluorescent molecule transported through the phloem vasculature and subsequently into the ovule through symplastic connections^55,56^. In all genetic backgrounds, the fluorescent tracer was visible in the funiculus and the chalaza, but not in the integuments of unfertilized ovules at 1 Day After Emasculation (1 DAE), indicating the absence of symplastic connectivity (Fig. 7a). However, at 3 DAP, we detected the CFDA tracer in the outer layers of the seed coat in wt, wt *pDD22::BAM5*, *abs* and *stk* single mutants, as symplastic movement is restored after fertilization (Fig. 7a)^6^. Conversely, in the *abs stk* double mutant and in *abs stk pDD22::BAM5* seeds, CFDA migration was still blocked in the chalazal seed coat at 3 DAP, consistent with the hypothesis of compromised nutrient flow during early seed development (Fig. 7a). Recently, it has been demonstrated that the symplastic transport of nutrients from the phloem end toward the seed is regulated by callose degradation in response to fertilization^10^. We thus investigated callose accumulation in unfertilized ovules and seeds by aniline blue staining. In both stages, signal was visible in a small group of cells in the chalaza, where the maternal vascular tissues terminates and the nutrient unloading occurs (Fig. 7b). The signal intensity of this specific area was measured as Corrected Total Cell Fluorescence (CTCF), revealing an increase in *abs stk* and *abs stk pDD22::BAM5* at both 1 DAE and 3 DAP with respect to all the other analyzed genotypes (Fig. 7c, d, Supplementary Fig. 9). Similarly, a larger area occupied by aniline blue signal was measured in both *abs stk* and *abs stk pDD22::BAM5* ovules and seeds compared to wt (Fig. 7c and Supplementary Fig. 9). The aniline blue quantification indicates an increased callose amount at the phloem pole of *abs stk* ovules and seeds, likely blocking symplastic flow in seeds. No difference was observed between *abs stk* and *abs stk pDD22::BAM5* plants, which correlates with CFDA tracer analysis (Fig. 7a). Interestingly, an intermediate value of signal intensity and area was observed in *stk* mutant ovules at 1 DAE with respect to wt and *abs stk*. However, in *stk* seeds at 3 DAP, the callose amount was similar to that of wt.

These results suggest that in the *abs stk* double mutant, sap flow from the sporophytic tissues to the embryo sac is impeded at the early stages of embryo development.

### Callose-related genes are regulated by *STK* and *ABS* in seeds at early developmental stages

**Fig. 8:**
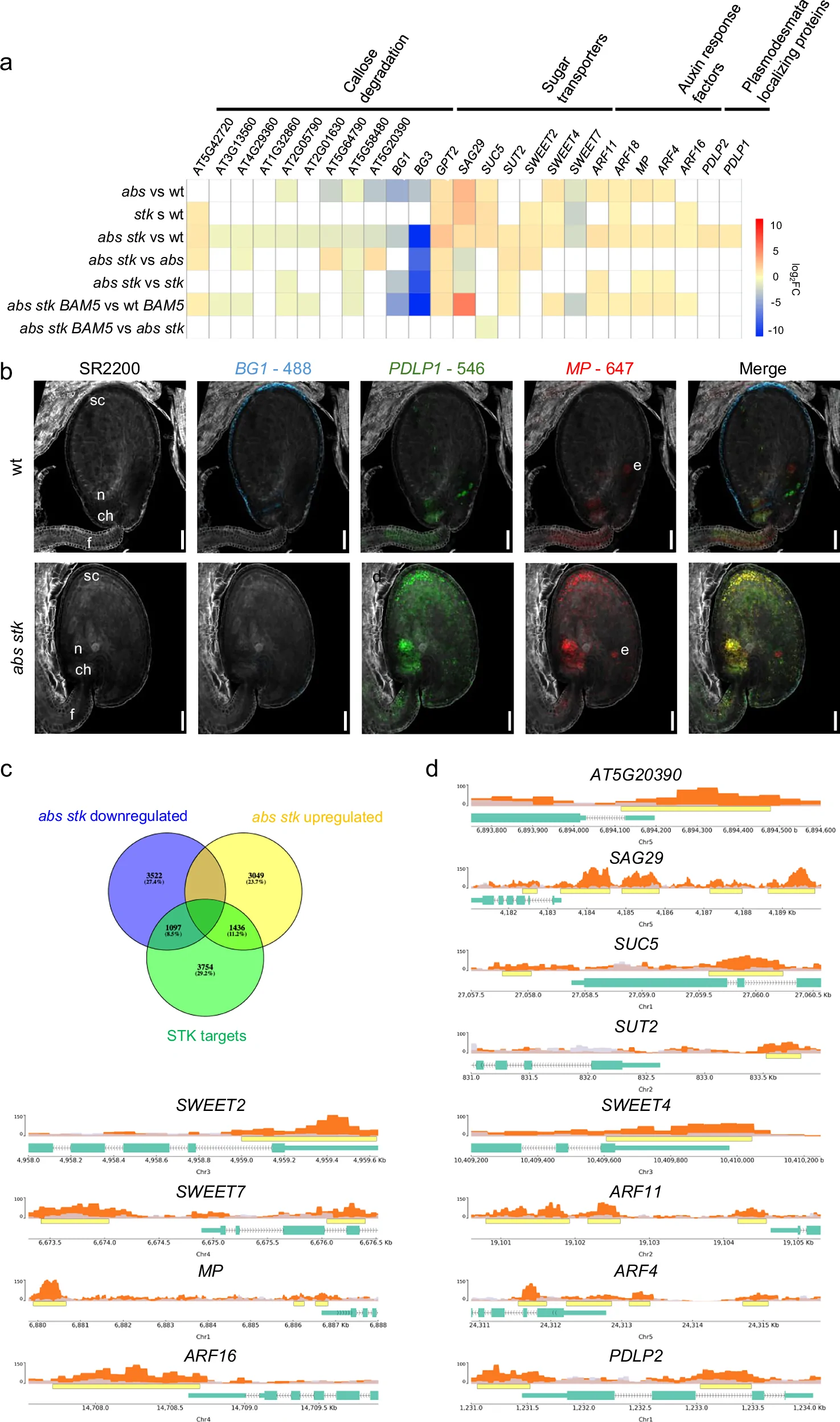
Genes involved in callose turnover, sugar transport, and auxin response are deregulated after fertilization by *ABS* and *STK* mutations. **a** Heatmap showing the DEGs involved in callose metabolism, sugar transport, and auxin response in the mutant-to-wt comparisons, in the double-to-single mutants comparisons, and in the *abs stk pDD22::BAM5* comparisons derived from RNA-seq data of post-fertilization samples. FDR ≤ 0.05 was considered to identify the DEGs. *n* = 4. The genes shown in this table were filtered for the DEGs in *abs stk* to wt comparison. Inside each category, the genes are ordered for the log_2_FC in *abs stk* to wt comparison from the highest (red) to the lowest (blue). **b** HCR-RNA FISH showing the expression of *BG1* (blue), *PDLP1* (green), and *MP* (red) in wt and *abs stk* 3 DAP seeds. Seeds are counterstained with SR2200 (white) to visualize cell walls. For each genotype, the same seed is shown as individual fluorescence channels for *BG1*, *PDLP1*, and *MP*, as well as in the merged image displaying all probes together. **c** Venn diagram representing the intersection between DEGs in *abs stk* vs wt comparison identified by RNA-seq in post-fertilization samples and STK targets identified by ChIP-seq in post-fertilization samples. **d** Visualization of STK binding sites identified by ChIP-seq in post-fertilization samples at the genes indicated in (**a**). Orange tracks represent the ChIP-seq enrichment signal; gray overlay tracks indicate the background signal; yellow boxes below the tracks mark the significant peak regions. Gene models are depicted with thin green boxes (utr), thick green boxes (exons), and connecting lines (introns); arrows indicate the direction of transcription. Ch chalaza, e embryo, f funiculus, sc seed coat. Scale bars 50 µm. Experiments were repeated on three independent individuals with similar results, and representative images are shown in (**b**).

The increased callose amount is consistent with blocked movement of CFDA and nutrient flow in the *abs stk* double mutant seeds, but not in the single mutants. This unique phenotype prompted us to examine callose-related genes whose expression was deregulated in the *abs stk* double mutants compared to the single mutants and wt. To this aim, we performed an RNA-seq experiment on siliques at 3 DAP (Supplementary Fig. 10, Supplementary Dataset 5). Recently, Liu and collaborators revised the number of genes predicted to be involved in callose degradation to a total of forty-two^10^. On the other hand, only twelve genes encoding callose synthesis have been reported in Arabidopsis^57^ (Supplementary Table 2). Among the DEGs in the *abs stk* double mutant compared to the wt, we identified eleven *β−1,3-glucanases* (*BG*), but no genes involved in callose biosynthesis (*glucan synthases*, *GSL*) (Fig. 8a). Notably, ten out of eleven *BGs* were downregulated in the *abs stk* mutant compared to wt (Fig. 8a), which correlates with higher callose accumulation and suggests that concerted activity of multiple hydrolytic genes may be required to remove the callose plug. Genes such as *BG1* and *BG3*, which are essential for callose degradation, were among the most downregulated in the *abs stk* double mutant compared to both wt and the single mutants (Fig. 8a), but they are not predicted to be expressed in the seed chalazal region in wt conditions according to snRNA-seq^58^ (Supplementary Table 2). We analyzed the *BG1* expression profile in wt and *abs stk* 3 DAP seeds through HCR-RNA FISH. In wt, *BG1* expression is confined to the seed coat and just above the callose plug in the chalaza, whereas it remains barely expressed only in the chalaza and micropylar seed coat in *abs stk* (Fig. 8b).

Recently, it has been proposed that auxin might modulate plasmodesmata aperture in roots through callose by the regulation of ARFs and plasmodesmata-localized proteins (PDLPs)^14,59^. Therefore, we considered whether a similar mechanism might be involved in callose deposition in the chalazal seed coat. Out of twenty-three *ARFs*, *ARF4*, *MONOPTEROS* (*MP*/*ARF5*), *ARF11*, *ARF16*, and *ARF18* were upregulated in the *abs stk* mutant compared to the wt at 3 DAP (Fig. 8a). Among them, *MP* was also upregulated in *abs* single mutant compared to wt, while *ARF16* was upregulated in *stk* vs wt, suggesting some specificity of ABS and STK in the regulation of *ARF* genes (Fig. 8a). Regarding *PDLPs*, *PDLP1* and *PDLP2* were upregulated in *abs stk* with respect to the wt, but, notably, not in the single mutant vs wt comparisons (Fig. 8a). Interestingly, only *MP, ARF18* and *PDLP1* were predicted to be expressed in the chalazal seed coat from snRNA-seq data, with *MP* and *PDLP1* having the higher expression in this region^58^ (Supplementary Fig. 11a, b, Supplementary Table 2). We thus evaluated the spatial expression of *MP* and *PDLP1* in wt and *abs stk* 3 DAP seeds, detecting a partially overlapping expression profile. Both genes were expressed in the funiculus, in the callose plug accumulation region, and in the persistent nucellus in the wt condition. *MP* expression was also detected in the embryo (Fig. 8b). In *abs stk*, these genes were ectopically expressed in the seed chalaza and in the seed coat, indicating clear deregulation of these genes.

Given that nutrient flow is impaired in the double mutant, we further examined the expression of genes encoding sugar transporters, including the families of *SWEET* and *SUC*, in the RNA-seq dataset. Of the seventeen *SWEETs*, three were upregulated in the *abs stk* mutant in comparison to wt, while only *SWEET7* was downregulated. Also, among the nine *SUC* genes, two were upregulated in the double mutant compared to the wt, and none were downregulated (Fig. 8a). None of these genes were predicted to be expressed in the seed coat chalazal domain^60^ (Supplementary Table 2), consistent with our hypothesis that the symplastic flow in *abs stk* seed chalaza is affected, rather than the apoplastic transport.

Collectively, most of the DEGs identified in the comparison of *abs stk pDD22::BAM5* to wt *pDD22::BAM* showed a similar trend when comparing *abs stk* to wt, suggesting that the embryo rescue mediated by the *pDD22::BAM5* construct was not related to callose turnover, sugar transport and the ARF-PDLP pathway, but likely only to starch mobilization (Fig. 8a).

The *STK* expression domain, but not *ABS*, partially overlaps with the chalazal seed coat domain in seeds at 1–3 DAP^60^ (Fig. 1d, Supplementary Fig. 1). To assess how many of these callose and solute transport-related genes might be direct targets of STK, we performed ChIP-seq on developing fertilized siliques of *pSTK::STK-GFP*. This revealed 8231 significant peaks, with a total of 6287 genes directly bound by STK (Supplementary Dataset 6). The STK binding profile across the genome region was similar in pre- and post-fertilization samples, with the majority of peaks primarily located in the promoter region of the target genes (compare Supplementary Fig. 4 and Supplementary Fig. 12a). The majority of STK targets were shared between pre- and post-fertilization samples (4382 genes), although there were several genes targeted by STK only in pre- (1103) or post-fertilization (1905) stages (Supplementary Fig. 12b and Supplementary Dataset 7). Thirteen post-fertilization STK targets involved in callose metabolism and sugar transport were also DEGs in the RNA-seq comparison of *abs stk* vs wt 3 in DAP siliques (Fig. 8c, d, Supplementary Table 2). Out of several callose turnover-associated genes differentially expressed in *abs stk* compared to wt, only one uncharacterized *β−1,3-glucanase* (AT5G20390) showed a peak in the promoter region (Fig. 8d). This suggests that ABS might be the primary regulator of callose removal in the seed chalaza, or intermediate factors regulated by *STK* and *ABS* act upstream of the callose hydrolytic machinery. Consistent with this hypothesis, four *ARFs*, including *ARF4, MP, ARF11* and *ARF16*, and one *PDLP* (*PDLP2*), were found to be either direct targets of STK and upregulated in the *abs stk* mutant compared to wt, with one or multiple peaks mainly localized in the promoter region (Fig. 8d). The other targets belonged to the category of sugar transporters (*SAG29/SWEET15*, *SUC5*, *SUT2*, *SWEET2*, *SWEET4*, *SWEET7*) (Fig. 8d).

Our results suggest that both STK and ABS appear to be part of a mechanism that regulates the expression of nutrient flux-related genes after fertilization.

### STK and ABS control callose gate opening after fertilization to ensure proper seed development

**Fig. 9:**
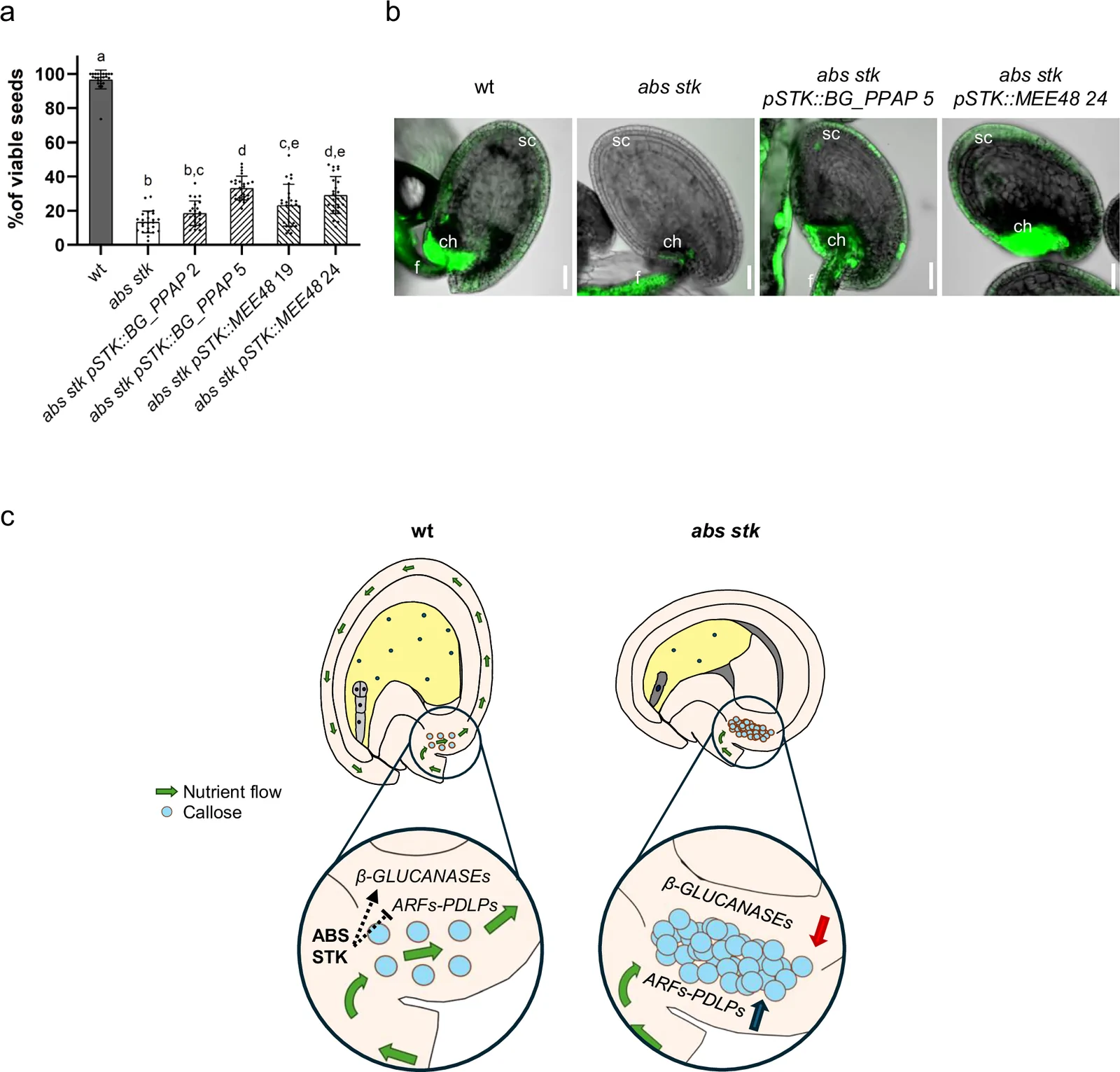
Ectopic degradation of callose partially rescues *abs stk* seed set. **a** Bar plot showing the percentage of viable seeds in siliques of wt, *abs stk*, *abs stk pSTK::BG_PPAP* 2, *abs stk pSTK::BG_PPAP* 5*, abs stk pSTK::MEE48* 19, and *abs stk pSTK::MEE48* 24 (*n* = 24). The graph represents the mean values ± SD, and the dots represent the single values. Different letters above the bars represent significant differences (*p* < 0.05) as determined by one-way ANOVA followed by Tukey’s HSD, whereas the same letters indicate absence of statistical difference between the respective genotypes. Measurements were repeated three times with similar results. Source data are provided as a Source data file. **b** Confocal images of seeds at 3 DAP showing CFDA symplastic tracer signal (green) in wt, *abs stk*, *abs stk pSTK::BG_PPAP* 5, and *abs stk pSTK::MEE48* 24. The GFP channel is merged with the brightfield channel to highlight the seed profile. **c** Putative model representing ABS and STK activity in regulating callose deposition and nutrient flow in the chalazal seed coat. Ch chalaza, f funiculus, sc seed coat. Scale bars 50 µm. Experiments were repeated on three independent individuals with similar results, and representative images are shown in (**b**).

To evaluate whether *abs stk* seed developmental defects were mainly due to the lack of callose degradation at the phloem end upon fertilization, we ectopically expressed callose-degrading enzymes in *abs stk* under the *STK* promoter, which is active in ovule and seed chalaza (Fig. 1d, Supplementary Fig. 1). We tested two characterized BGs, BG_PPAP and MEE48, in parallel. Constitutive expression of *BG_PPAP* is able to increase nutrient transport in the Arabidopsis seed^10^, while a *MEE48* ortholog was previously used to reduce callose deposition in Arabidopsis ovules^61^. Of the two independent transformant lines analyzed for each construct, one *abs stk pSTK::BG_PPAP* line and two *abs stk pSTK::MEE48* lines partially rescued the seed set compared to the *abs stk* mutant. The percentage of developed seeds in siliques of the rescue lines was between 23% and 33% compared to the 13% observed in *abs stk* (Fig. 9a). We confirmed reduced aniline blue fluorescence in the chalaza of the rescue lines compared to the *abs stk* mutant, consistent with a reduced callose level, yet still significantly higher with respect to wt (Supplementary Fig. 13). We thus evaluated whether the reduction in callose amount restored the nutrient flow in the *abs stk* seeds by visualizing CFDA tracer. Remarkably, in 3 DAP seeds of *abs stk pSTK::BG_PPAP* and *abs stk pSTK::MEE48* rescue lines we observed a low percentage (5-10%) of seeds exhibiting fluorescence in the seed coat, likely because callose removal was incomplete (Fig. 9b). Nonetheless, the fluorescence percentage observed in the rescue lines of *abs stk pSTK::BG_PPAP* and *abs stk pSTK::MEE48* was notably higher than that of the *abs stk* mutant, where none of the seeds exhibited a CFDA signal in the seed coat. In the mutant, the tracer was entirely obstructed in the seed chalaza across all seeds.

These results indicate that the defects in seed set observed in the *abs stk* mutant are at least partially attributed to the lack of callose degradation in the chalaza, which in turn impacts seed development.

Taken together, our findings support the hypothesis that the callose degradation at the phloem end gate, which regulates symplastic nutrient flux in the seed chalaza, is controlled by ABS and STK. This regulation may involve both direct and non-cell autonomous complex networks, including genes encoding callose-degrading enzymes (*BGs*), together with *ARF*-*PDLP* modules (Fig. 9c).

## Discussion

The crosstalk between the female gametophyte and the sporophytic tissues surrounding it is fundamental for ovule development^62–64^. In *abs stk* ovules, the inner integument fails to differentiate correctly, and endothelium identity is lost^19^. Moreover, *abs stk* mutants show defective embryos that lead to high seed sterility. Although the inner integument is needed for the correct development of a mature female gametophyte^65^, genetic ablation of the endothelium in ovules and seeds still results in the formation of viable embryo and endosperm^27,65^. In *abs stk* ovules, no apparent defects are observed in female gametophyte formation, suggesting that the two layers of the inner integument are sufficient for gametogenesis. Furthermore, our data presented here suggest that the developmental defects observed during embryogenesis in the double mutant are likely independent of the lack of endothelium differentiation. Hence, the basis for the post-fertilization defects in *abs stk*, and the pathways involved, may have been overlooked in previous studies. Other than the lack of endothelium differentiation, the double mutant presents a range of phenotypes. For example, *abs stk* seeds possess rounder, smaller seeds and impaired seed coat expansion compared to wt (Fig. 1). This phenotype can be attributed to the *stk* mutation, which has previously been associated with reduced seed coat cell elongation and smaller seeds, with reduced length^34,66^. Nevertheless, the *stk* mutation alone is not responsible for the fertility defects that characterize the *abs stk* double mutant, as the percentage of viable seeds in the double mutant is significantly lower than in the *stk* single mutant (Fig. 5). In addition, young seeds of *abs stk* show a lack of nucellus degeneration, but this phenotype is also observed in *abs* mutants that still show higher levels of seed fertility. Again, these data point towards other ABS- and STK-dependent ovule pathways being compromised that contribute to seed developmental defects.

Although the *abs stk* ovules harbor a mature gametophyte, the lack of *ABS* and *STK* function in the sporophytic tissues impacts the development of the embryo (Fig. 6) and endosperm^19^. These phenotypes are of sporophytic maternal origin^67^, as the double heterozygotes *abs/ABS stk/STK* plants, or even the *abs/ABS stk/stk* or *abs/abs stk/STK* plants, do not present the same fertility defects^19^. In barley and wheat, the Bsister MADS-box gene *MADS31* is sporophytically expressed in the ovule nucellus, which differentiates to become the primary solute transfer tissue in the grain. *mads31* mutants exhibit defects in nucellus differentiation, partial defects in gametophyte development, and reduced seed set^68^. Even if the *abs* mutation alone does not affect gametophyte development and seed set in Arabidopsis, the role of Bsister genes in controlling communication between the sporophytic and gametophytic generations may be conserved. A previous study showed that at 3 DAP, only half of the *abs stk* seeds contain a developing endosperm; however, the percentage of developing embryos had not been investigated^19^. Here, by analyzing the presence of zygotes, we demonstrated that the EC is correctly fertilized in *abs stk* seeds (Fig. 6). However, embryo development is delayed and arrests at later stages (Supplementary Fig. 7). It has been shown that mutations in sucrose transporter-encoding genes (*SWEET4/11/12*/*15* and *SUC5*), which are expressed in the persistent nucellus and seed coat, cause a delay in embryo development including a reduction of the size of the embryo and the whole seed^9,18,39^. Nonetheless, the *sweet11/12/15* and *suc5* mutants form a normal embryo. Most of the sugar transporter-encoding genes are upregulated or expressed normally in the *abs stk* mutant compared to wt pre- and post-fertilization, suggesting that other factors are likely involved in embryo arrest.

Many genes involved in the starch and sucrose metabolic pathways are deregulated in the *abs stk* double mutant pistils, possibly contributing to the starch over-accumulation observed in double mutant ovules (Figs. 1 and 2). Previous studies showed that *STK* is involved in the modulation of key sugar metabolism genes during seed coat development^32,33^, but no information is available for *ABS*. Our data demonstrate that STK is a direct transcriptional regulator of genes involved in starch breakdown, trehalose and sugar transport. Members of the glucan water dikinase (GWD) and amylase (AMY and BAM) families play a crucial role in catalyzing the hydrolysis of this polyglycan into maltose units^37^. *GWD1* and *BAM4*, direct targets of STK, were downregulated in the *abs stk* double mutant and in the single mutant compared to wt (Figs. 2 and 3). Furthermore, *BAM4* expression was confined to the nucellus of unfertilized *abs stk* ovules and absent from the embryo sac (Fig. 2). However, only the *abs stk* double mutant accumulated more starch in ovules, and this phenotype is likely caused by a general imbalance of the sugar metabolic pathways. For example, *GPT2*, encoding a translocator of Glucose 6-P from plastids to build starch, is ectopically expressed in *abs stk* ovules. Notably, the expression domain of *GPT2* overlaps with the ovule region where a significant amount of starch is present in the *abs stk* double mutant (Figs. 1 and 2). We also report higher levels of trehalose in the *abs stk* mutant in comparison with the wt and the single mutants (Fig. 4). Higher trehalose levels may inhibit starch degradation and induce its overaccumulation^69^. The downregulation of *TRE1*, a direct STK target, in the *abs stk* double mutant ovules possibly contributes to higher trehalose accumulation and, in turn, increases the quantity of starch (Fig. 2).

ABS and STK interact with SEP3 in yeast, suggesting they may form a complex^70^. However, the localization of STK and ABS proteins in ovules and seeds suggests that they act in different domains. STK is mainly expressed in the outer integuments and the funiculus, whereas ABS is detected in the nucellus and the two inner integuments (Fig. 1). RNA-seq and ChIP-seq data indicate that both genes are involved in the regulation of the sugar metabolic pathway before fertilization (Supplementary Table 1). This regulation should occur, at least partially, in a non-cell autonomous manner, as *ABS* and *STK* are not detected in the embryo sac, where the largest starch deposit is present in the *abs stk* mutant. Some of the STK direct targets are also regulated by ABS, as they are differentially expressed in the *abs* single mutant in comparison to the wt. Nevertheless, other metabolic genes are differentially expressed exclusively in one of the two single mutants compared to wt, suggesting that these two TFs could regulate genes from the same pathway in a redundant manner, but in different domains of ovules and seeds. Consistent with this hypothesis, in seeds, nutrient transporters belonging to the UMAMITs, for amino acids, and SWEETs, for sucrose, specifically mark distinct seed compartments^8,9,71^. From these domains, they transport nutrients from the sporophytic tissues to the fertilization products, and since multiple transporters exist, the mutation of single genes does not compromise seed viability^8^.

Our results suggest that the fine-tuned pre- and post-fertilization sugar balance has a major impact on early seed development. Moreover, this balance relies on the communication between the sporophyte and gametophyte. In fact, we rescued embryo development by enhancing starch mobilization in the embryo sac and endosperm of *abs stk* seeds (Fig. 5). In wt seeds, as soon as double fertilization takes place, the starch accumulated in the CC is broken down, likely mobilizing nutrients to sustain the developing embryo, and potentially allowing for further influx of sugars. In *abs stk*, a larger starch deposit is observed in the embryo sac of unfertilized ovules. Similarly, starch is maintained unused after fertilization^19^, and fertility is only restored through ectopic expression of *BAM5*. This suggests that sugar transport pathways are functional prior to fertilization, but their turnover appears to be compromised. Starch degradation may therefore increase the mobilization of free sugars to support embryo growth just after fertilization. Sugars are transported to the ovules and seeds by long-distance transport through the phloem tissues, which end in the chalazal region. Here they are symplastically unloaded just after fertilization and transported to the other seed domains^6^. The phloem unloading occurs in wt and *abs* and *stk* single mutant seeds, but is blocked in the *abs stk* double mutant, as suggested by analysis of CFDA, an exogenous marker for symplastic transport (Fig. 7). The absence of nutrient transport from the phloem end gate to the embryo sac likely causes starvation that compromises the early stages of zygote development in *abs stk* seeds. Recently, a strong relationship has been demonstrated between sugar signaling and cell cycle progression^72^; this supports our hypothesis that the altered sugar accumulation and metabolism in *abs stk* seeds may contribute to early stages of embryo division and growth. Considering this, our data also demonstrate that enhancing the sugar availability in the embryo sac induces division in the endothelial cell layer (Fig. 5).

Recently, the nutrient supply to the seed has been related to callose removal at the chalazal phloem end in response to successful fertilization. In the fertilization-defective *generative cell-specific 1* (*gcs1*) mutant, nutrient flow is not triggered due to increased callose accumulation, preventing the waste of nutrients^10^. Callose over-accumulates at the phloem end region of *abs stk* seeds, blocking transport, and the starch mobilization in the embryo sac is not sufficient to induce callose removal (Fig. 7). Similarly, the *AtBG_ppap* loss-of-function mutation, disrupting a putative plasmodesmata-associated β−1,3-glucanase, causes increased callose accumulation in the chalazal seed domain and smaller seeds. However, in the *Atbg_ppap* mutants, the transport of nutrients is not completely compromised, and seed viability is not affected, indicating that other redundant genes may control callose gate opening at the phloem end gate^10^.

Our RNA-seq results indicate that many genes involved in callose degradation are downregulated in the *abs stk* double mutant after fertilization, possibly contributing to impaired callose removal (Fig. 8). Notably, one of the most downregulated genes in the *abs stk* double mutant is *BG1*, which has already been linked to the deficiency in callose degradation observed in the *gcs1* mutant^10^. *BG1* is not expressed in the callose plug region in the seed coat chalazal domain, suggesting it could act in a non-cell autonomous way, or most likely, other β−1,3-glucanases may regulate callose gate opening (Fig. 8). For example, two uncharacterized *BGs* that are differentially expressed only in *abs stk* siliques compared to wt (AT2G01630, AT1G32860) are predicted to be expressed in the chalazal seed coat domain, thus may be directly involved in the absence of callose removal in *abs stk* seeds (Supplementary Table 2). It has been reported that auxin plays a role in modulating plasmodesmata aperture through the interplay between the *ARFs* and *PDLPs*^11,14,59^. Plants overexpressing *PDLP5* are characterized by increased callose levels and suppressed trafficking through plasmodesmata^14^. In the *abs stk* double mutant, the ectopic expression of *ARF5/MP* and *PDLP1* genes in seeds might contribute to the impermeability of plasmodesmata by callose deposition, thus blocking nutrient flux to the seed (Fig. 8). Supporting this hypothesis, we demonstrated that ectopic expression of *BG_PPAP* or *MEE48*, encoding another β−1,3-glucanase, in the *STK* expression domain is sufficient to reduce the callose level in the chalazal region of *abs stk*. This contributes to increased *abs stk* seed fertility and a low rescue of nutrient unloading, likely due to incomplete callose degradation, supporting a role for ABS and STK in the regulation of this process. Although our results suggest that *ABS* and *STK* directly or indirectly regulate callose deposition via *BGs* and *ARF-PDLP* modules, further analyses are needed to identify the signal(s) that induce callose degradation and opening of the phloem end gate.

Our findings demonstrate the importance of STK and ABS, two MADS-box transcription factors, in controlling ovule development and the flux of solutes between the maternal sporophyte and the next generation. The two proteins appear to act in concert from different ovule tissues to support sugar accumulation and metabolism, which is required for embryo growth, and to subsequently remove callose barriers adjoining the nucellus and seed coat that are required for post-fertilization seed development. Thus, ABS and STK provide a critical regulatory link between maternal nutrient supply and seed developmental processes.

## Methods

### Plant material and growth conditions

All plants used in this study were of the Columbia-0 (Col-0) accession. Seeds were sown either directly on soil or on Murashige and Skoog (MS) medium^73^ and then moved to soil. Before sowing in germination medium, seeds were surface-sterilized by incubation in 0.1% Triton X-100 for 30 min, followed by 0.1% Triton X-100 and 10% bleach for 10 min, and rinsed three times with sterile water. After 10 days, the seedlings were moved to soil and grown in a controlled growth chamber under long-day conditions (16 h light/8 h dark) at 22 °C. The *abs* (*tt16-6*) and *stk* (*stk-2*) mutants were already described^30,74^. Mutants were screened using PCR with specific oligonucleotides listed in Supplementary Dataset 9. The *pSTK::STK-GFP* marker line used in this work was previously generated^29^. The *pABS::ABS-GFP* was kindly provided by E. Magnani and previously described^3^, and the *pWOX9::WOX9-GFP* was previously described^52^.

### Plasmid construction and Arabidopsis transformation

The expression vector pK2GW7 was modified by Doulix (Venice, Italy) to substitute the *p35S* followed by the Gateway cassette with the synthetically generated *pDD22* promoter upstream of the CDS of *BAM5*. For the *DD22* promoter, 935 bp before ATG were considered, as described previously^50^. To generate *pSTK:BG_PPAP* and *pSTK:MEE48* constructs, the relative CDS were cloned downstream of the *STK* promoter (−1840/+1088) in the pBGW0 expression vector with the primer described in Supplementary Dataset 9. The resulting expression vectors were transformed into *Agrobacterium tumefaciens* by electroporation, and Arabidopsis plants were transformed using the floral dip method^75^. Seeds of transformed plants were selected on kanamycin (100 mg L^−1^) (*pDD22:BAM5*) or Basta herbicide (*pSTK:BG_PPAP* and *pSTK:MEE48*) on MS medium or directly on soil, respectively. The presence of the construct was assessed by PCR with the oligonucleotides listed in Supplementary Dataset 9. Two or three independent T1 lines were selected, depending on the experiment, and the T2 plants were further analyzed.

### Fertility analyses

The fertility analysis was performed by opening mature siliques of wt, *abs*, *stk*, *abs stk*, wt *pDD22::BAM5*, *abs pDD22::BAM5, stk pDD22::BAM5*, and *abs stk pDD22::BAM5*, counting the number of viable seeds per silique using a Leica MZ6 stereomicroscope (25 siliques for each genetic background). Images were acquired and processed using Axiovision (version 4.1) software. To complement the silique analysis, we quantified the percentage of developing embryos using a marker line carrying the *pWOX9::WOX9-GFP* construct. These plants (used as pollen donors) were crossed with wt *pDD22::BAM5*, *abs pDD22::BAM5, stk pDD22::BAM5, abs stk pDD22::BAM5*, and *abs stk*. The derived siliques were collected at 3 DAP, dissected in water on a glass slide to obtain seeds, and finally observed using a Nikon A1 confocal laser scanning microscope to count the number of seeds expressing the GFP signal (15 siliques for each genetic background). GFP was excited with a 488 nm laser, and emission was detected between 520 and 540 nm. Confocal images were analyzed using Fiji software^76^. The seed set analysis for the *pSTK::BG_PPAP* and *pSTK::MEE48* lines was performed as described above (24 siliques for each genetic background). The statistical differences were calculated using one-way ANOVA with post-hoc Tukey’s HSD test (*p* < 0.05).

### Feulgen staining of ovules

For the morphological characterization of mature ovules, pistils were isolated from flowers just before anthesis (stage 12c)^77^. The reagents and protocol used to perform Feulgen staining were described previously^78^. The samples were excited with a laser at 488 nm and emission was detected between 515 and 600 nm. Images were captured using a Nikon A1 confocal laser scanning microscope. Confocal images were analyzed using Fiji software^76^.

### Lugol staining of ovules

To visualize starch accumulation, pistils were isolated from flowers just before anthesis (stage 12c)^77^. Starch staining was performed with Lugol’s solution as previously described^19^. We then dissected the pistils in water to isolate mature ovules. The signal was observed using a Zeiss Axiophot D1 microscope equipped with DIC optics. Images were recorded with an Axiocam MRc5 camera (Zeiss) using the Axiovision program (version 4.1).

### Expression analysis by in situ hybridization

In situ hybridization and probe synthesis were performed as described previously^79^. Arabidopsis inflorescences before anthesis were sampled, fixed, and embedded in paraffin. Plant tissue sections (8 μm) were probed with antisense digoxigenin (DIG)-labeled probes. RNA probes were amplified using the oligonucleotides described in Supplementary Dataset 9. The signal was observed using a Zeiss Axiophot D1 microscope equipped with DIC optics. Images were recorded with an Axiocam MRc5 camera (Zeiss) using the Axiovision program (version 4.1).

### Expression analysis by HCR-RNA FISH

Arabidopsis pistils and siliques were collected at 1 day after emasculation (1 DAE) and at 3 days after pollination (3 DAP), respectively. Prior to fixation, samples were dissected to maximize exposure of the tissues of interest, thereby facilitating confocal imaging and minimizing the need for deep tissue acquisition. Whole-mount HCR-RNA FISH was performed following a protocol adapted from Huang et al.^80^. In particular, all incubation and washing steps were carried out under continuous agitation (200–300 rpm). Samples were fixed in 4% formaldehyde solution, and tissue permeabilization (i.e., cell wall digestion) was conducted using Enzyme Mix A, applied twice for 20 min each rather than a single 3 min treatment as described in the original protocol^81^. HCR-RNA FISH hybridization and amplification were conducted according to the third-generation HCR methodology previously described^81^, and the protocols provided by Molecular Instruments. Probe sets targeting the transcripts of interest were designed and synthesized by Molecular Instruments. Amplification was performed using X1-488, X2-647, and X3-546 HCR amplifier hairpins. Hybridization probes and amplification hairpins used in this study are listed in Supplementary Table 3. For probe hybridization, samples were pre-incubated in HCR Probe Hybridization Buffer (Molecular Instruments) for 15 min at 37 °C. The hybridization solution was prepared by adding 1 μL of each probe set (1 μM stock) to 100 μL of pre-heated HCR Probe Hybridization Buffer. Samples were incubated in the probe solution overnight (~20 h) at 37 °C. Following amplification, cell walls were counterstained with Renaissance SR2200 (reference). Samples were incubated in SR2200 staining solution for 3 h and subsequently washed with SSC-T buffer before confocal imaging.

### RNA-seq material and data analysis

Total RNA was extracted from a pool of isolated pistils from stage 9 to stage 12^77^ of wt, *abs*, *stk*, and *abs stk*, in four biological replicates, using the Macherey Nagel “Nucleospin RNA Plant” kit following the manufacturer’s instructions (pre-fertilization samples). For post-fertilization samples, RNA was extracted in the same way from a pool of 3 DAP siliques of wt, *abs*, *stk*, *abs stk*, wt *pDD22::BAM5*, and *abs stk pDD22::BAM5*, in four biological replicates. mRNA-seq with stranded oligo-dT-specific library preparation and sequencing of 20 million PE150 reads was performed by Biomarker Technologies (BMK) with the Illumina NovaSeq X platform. The obtained raw reads were trimmed with Trimmomatic 0.39^82^, with the following parameters: ILLUMINACLIP:1:30:6, LEADING: 0, TRAILING: 20, AVGQUAL: 20, MINLEN: 35. STAR 2.7.10b^83^ was used for mapping, with --alignIntronMax 10000 on the TAIR10 genome. Counts were obtained using featureCounts 2.1.1^84^, using Araport11 annotation, with the following parameters: -p, - -countReadPairs. HTSFilter^85^ was used to filter the counts. Principal component analysis (PCA) was performed to identify clustering between the four biological replicates of each sample. DESeq2^86^ was used to obtain the DEGs. Only DEGs with an FDR ≤ 0.05 were considered statistically significant. DEG analysis was performed on RStudio, version 4.4.1. Venn Diagrams were generated with the online tool Venny 2.1.0. The functional enrichment analysis of RNA-seq (KEGG) was performed using the online tool ShinyGO 0.81 with an FDR cut-off value of 0.05 to select the enriched categories.

### ChIP-seq material and data analysis

ChIP-seq was performed as described previously^87,88^ with the following modifications. Approximately 1 g of inflorescences with flowers until stage 12 (pre-fertilization) and siliques from stage 14 to stage 16 (post-fertilization) of *stk* plants complemented with *pSTK::STK-GFP*^29^, in three biological replicates, were crosslinked with 1% (v/v) formaldehyde under vacuum for 2 × 15 min, with mixing in between. The crosslinking reaction was stopped by adding glycine to 0.25 M concentration. The fixed tissue was ground in liquid nitrogen, followed by nuclei isolation and chromatin extraction. The chromatin was sonicated using the S220 (Covaris) sonicator with the following settings: Peak Power = 140.0; Cycles/Burst = 200; Duty Factor = 5.0; Duration = 590 s; temp = 6 °C. Samples were centrifuged, and a portion of the sonicated chromatin (supernatant) was set aside as input control. Five microliters of anti-GFP antibody (Abcam, ab290) was added to the sonicated chromatin and mixed at 4 °C for 1 h. A portion of µMACS Protein A MicroBeads (Miltenyi Biotec) was blocked with 0.5% BSA in IP buffer, mixed at 4 °C for 6 h and added to the mix. The immunoprecipitated chromatin was captured by mixing at 4 °C for 1 h. The samples were passed through a magnetic µColumn (Miltenyi Biotec) fixed on a µMACS Separator (Miltenyi Biotec) and washed three times with each IP buffer, high-salt buffer, LiCl buffer, and TE buffer. The immunoprecipitated chromatin was eluted with hot elution buffer (1% SDS, 0.1 M NaHCO_3_). To reverse the crosslinking, input and eluate samples were incubated with 250 mM NaCl in a thermomixer at 65 °C for 16 h and 1000 rpm, followed by incubation with 0.615 mg/ml proteinase K at 65 °C for 1 h and 1000 rpm. The de-crosslinked DNA was ethanol-precipitated and purified using the DNA Clean & Concentrator kit (Zymo Research). The ChIP-seq library was prepared with the ThruPLEX DNA-seq Kit (Takara Bio) following the manufacturer’s instructions. The DNA libraries were size-selected for 100-700 bp fragments with the AMPure XP beads (Beckman Coulter). The size-selected libraries were assessed on a 4200 TapeStation (Agilent) and the concentration measured using a Qubit 4 instrument (Invitrogen). The libraries were sequenced using an Illumina high-throughput sequencing device.

The quality of the raw sequencing reads was assessed with *FastQC*. Potential adapter sequences were removed from the sequencing reads using *Trimmomatic*^82^, and the quality of trimmed reads was reassessed. Trimmed reads were mapped to the Arabidopsis TAIR10 reference genome using *Bowtie2*^89^. Only reads with MAPQ score >40 were kept. Peak calling and fragment pileup were performed using *Macs2*^90^ with parameters: *-p 0.05 -B –SPMR –mfold 2 20 -g 101274395*. For the STK-GFP ChIP-seq, the regions declared significant by *Macs2* were merged using *mergeBED* from the *bedtools* package^91^ with parameters: *-iobuf 5G -d 100* to obtain a common reference set to later test with *DESeq2*^86^. Finally, the R package ChIPseeker (annotatePeak) was used to annotate the peaks on the reference genome with a distance to TSS of −3000 and +3000 bp^92,93^. The functional enrichment analysis of ChIP-seq (GO and KEGG) was performed using the online tool DAVID with an FDR cut-off value of 0.05 to select the enriched categories. Venn Diagrams were generated with the online tool Venny 2.1.0.

### Extraction, identification, and quantification of primary metabolites in Arabidopsis pistils

The dissected pistils from flowers before fertilization (from stage 11 to 12^94^) were collected at 12.00 am and immediately frozen in liquid nitrogen to quench metabolism. Freshly homogenized (50 mg) pistils were obtained for each biological replicate (4) and genotype (Col-0, *abs*, *stk*, and *abs stk*). Metabolite extraction and derivatization for untargeted metabolomics were carried out following the protocols described by Lisec et al.^95^. The derivatized extracts were injected into a 5MS capillary column (30 m × 0.25 mm × 0.25 µm) (with 10 m of pre-column) (Thermo Fisher Scientific, Waltham, MA, USA) using a single quadrupole GC/MS. Injector and source were set at 250 °C and 260 °C. 1 µl of the sample was injected in a splitless mode with a helium flow of 1 ml/min using the following programmed temperature: isothermal 5 min at 70 °C followed by a 5 °C/m ramp to 350 °C and a final 5 min heating at 330 °C. Mass spectra were recorded in electronic impact (EI) mode at 70 eV, scanning at 40-600 m/z range, scan time 0.2 s. The mass spectrometric solvent delay was set at 9 min. Pooled samples that served as quality control (QCs), n-alkane standards (C8-C40 all-even), and blanks were injected at scheduled intervals for instrumental performance, tentative identification, and monitoring of shifts in retention indices (RI). The RAW files were analyzed using MS-DIAL version 4.20 (http://prime.psc.riken.jp/compms/msdial/main.html)^96^. The centroided EI-MS data with at least 20 data points across the peaks were considered for further deconvolution using a sigma window value of 0.5. Spectral annotation was performed using an open-source EI-spectra reference assembled as described by Misra (2019). A retention index (RI) reference generated in-house with n-alkanes as described above was used for RI matching, and a cosine similarity of 70% was used as a cut-off for spectral matching with a reference database for compound ID assignment. For metabolite annotation, we followed the Metabolomics Standards Initiative (MSI) levels of the International Metabolomics Society: annotations were considered level 2 (putative annotation based on spectral library similarity) or level 3 (putatively characterized compound class based on spectral similarity) to known compounds of a chemical class as suggested (Sumner et al.)^97^. Spectral features found in blank runs were filtered out when present >2-fold higher than in samples on average. For relative quantification purposes, when we encountered multiply silylated (n-TMS) features of well-annotated metabolites, we retained the major (more abundant) compounds and left out other minor (low abundance) versions for consistent comparison across all samples. A matrix of annotated metabolites and their corresponding abundances across all samples were used for further processing, summary statistics, and data visualization. The metabolomic experiments were carried out adopting a completely randomized design with five replications. Statistical analysis for the GC/MS dataset was performed using MetaboAnalyst version 4.0^98^. Briefly, relative abundance values from the MS-DIAL outputs were normalized using the QC and the internal standard ribitol as the reference standard, then were log-transformed and Pareto scaled. Normalized data were successively statistically analyzed through univariate (one-way ANOVA using LSD test as post-hoc, *p* < 0.05) and multivariate analysis [unsupervised principal component (PCA) analysis, and supervised PLS-DA analysis]. To avoid overfitting, the PLS-DA model was validated using Q2 as a performance measure, the 10-fold cross validation and setting in the permutation test a permutation number of 20. Data were further represented through a heatmap, built on the top 60 compounds resulting from the ANOVA analysis, and hierarchically clustered using the Euclidean distance measurement and Ward as a clustering algorithm.

### Confocal microscopy of embryos

To analyze the percentage of developing zygotes and embryos, siliques from hand-pollinated pistils were collected at 1 DAP and 2 DAP. Samples were treated with 0.2 M NaOH 1% (v/v) SDS solution for 3 h at 37 °C, rinsed three times with distilled water, and finally moved in 2% (v/v) bleach solution at room temperature for 10 min. Siliques were rinsed five times with distilled water, then dissected using needles on a glass slide, and seeds were stained with SR2200 and Propidium Iodide (PI) solution (Merck). Images were acquired using a Nikon A1 confocal laser scanning microscope. SR2200 was excited with a 405 nm laser line, and emission was detected between 440 and 470 nm, whereas PI was excited with a 561 nm laser line and emission was detected between 660 and 730 nm. Confocal images were analyzed using Fiji software^76^.

### Aniline blue staining of ovules and seeds

Emasculated pistils (1 DAE) and hand-pollinated siliques (3 DAP) were treated as reported previously^10^. Samples were collected in 1 M NaOH solution and left overnight at room temperature. Afterwards, the samples were incubated in aniline blue solution [1% (w/v) aniline blue and 0.1 M K_3_PO_4_] for at least 3 h. Ovules/seeds were isolated with needles on a glass slide and observed using a Nikon A1 confocal laser scanning microscope. The samples were excited with a laser at 445 nm and emission was detected between 490 and 545 nm. Z-stack images were acquired using a Nikon A1 confocal laser scanning microscope (Z-step 0.5 µm). 3D images were obtained using the 3D project tools on Fiji software^76^. Aniline blue signal was quantified on Z-project images (Sum Projection) with Fiji software^76^. CTCF was calculated by subtracting the integrated density of the ROI (region of interest) in the chalazal phloem end and the integrated density of the same ROI in the background of the image. The ROI was defined using a thresholding method to discriminate the signal from the background noise.

### CFDA tracer detection in ovules and seeds

Pistils (1 DAE) and hand-pollinated siliques (3 DAP) were cut with a sharp blade at the root of the silique pedicel and placed in PCR tubes containing 100 μl of 5(6)-Carboxyfluorescein diacetate (CFDA) solution (0.1 mg/ml, Merck) at 22 °C for 4 h^10^. The pistils and siliques were dissected with needles on a glass slide under a stereomicroscope to isolate ovules and seeds and mounted in water. Images were acquired using a Nikon A1 confocal laser scanning microscope. The CFDA was excited with a laser at 488 nm, and emission was detected between 520 and 540 nm. Confocal images were analyzed using Fiji software^76^.

### Seed coat cells measurements

Siliques derived from hand-pollinated pistils (2 DAP) were treated as described in the “Confocal microscopy of embryos” paragraph. Seeds were stained with SR2200. Images of seeds were acquired using a Nikon A1 laser scanning confocal microscope. SR2200 was excited with a 405 nm laser line, and emission was detected between 440 and 470 nm. Confocal images of seeds were analyzed using Fiji software^76^, measuring the number and length of oi2 cells, the total seed area, the seed cavity area and the seed aspect ratio (AR) (15 seeds for each genetic background). Statistical differences between the different genotypes were analyzed using one-way ANOVA (total seed area, oi2 number of cells, seed cavity area, seed aspect ratio) or nested ANOVA (oi2 cell length) with post-hoc Tukey’s HSD test (*p* < 0.05).

### Statistics and reproducibility

Apart from RNA-seq, ChIP-seq and metabolomic data analyses, described in the Methods section, statistic was performed using one-way ANOVA with post-hoc Tukey’s Honestly Significant Difference (HSD) test with the GraphPad Prism software (version 8 for Windows), unless otherwise stated. *p* value < 0.05 was interpreted as statistically significant. Other details such as the number of replicates, the sample size, the individual values and the level of significance are specified in the text, figure legends and/or Source data. The experiments shown in the paper were repeated at least three times, unless otherwise stated.

### Reporting summary

Further information on research design is available in the Nature Portfolio Reporting Summary linked to this article.

## Supplementary information

Supplementary Information

Description of Additional Supplementary Files

Supplementary Data 1

Supplementary Data 2

Supplementary Data 3

Supplementary Data 4

Supplementary Data 5

Supplementary Data 6

Supplementary Data 7

Supplementary Data 8

Supplementary Data 9

Reporting Summary

Transparent Peer Review file

## Source data

Source data

## Data Availability

The RNA-seq raw data generated in this study have been deposited to the NCBI under the accession PRJNA1492831 and PRJNA1492637. The ChIP-seq raw data generated in this study have been deposited to the NCBI under the accession PRJNA1491678. RNA-seq and ChIP-seq final processed data (list of DEGs and STK target genes) are included in Supplementary Datasets 1, 2, 5 and 6. Metabolomic data are included in Supplementary Dataset 4. All the materials of the study are available from the corresponding author upon request. Source data are provided with this paper.
