# Supplementary material for: ARABIDOPSIS Bsister and SEEDSTICK MADS-box transcription factors modulate maternal nutrient flow for seed development in Arabidopsis
Source: Nat Commun. 2026 Sep 10;17:10687. doi: 10.1038/s41467-026-77573-2 (PMC13655123; doi:10.1038/s41467-026-77573-2)
Supplement: Supplementary file 1 [file 41467_2026_77573_MOESM12_ESM.pdf]

nature portfolio

Corresponding author(s): Lucia Colombo
Last updated by author(s): Aug 25, 2026

Reporting Summary

Nature Portfolio wishes to improve the reproducibility of the work that we publish. This form provides structure for consistency and transparency in reporting. For further information on Nature Portfolio policies, see our Editorial Policies and the Editorial Policy Checklist.

Statistics

For all statistical analyses, confirm that the following items are present in the figure legend, table legend, main text, or Methods section.

n/a	Confirmed	
☐	☒	The exact sample size (*n*) for each experimental group/condition, given as a discrete number and unit of measurement
☐	☒	A statement on whether measurements were taken from distinct samples or whether the same sample was measured repeatedly
☐	☒	The statistical test(s) used AND whether they are one- or two-sided<br>*Only common tests should be described solely by name; describe more complex techniques in the Methods section.*
☒	☐	A description of all covariates tested
☐	☒	A description of any assumptions or corrections, such as tests of normality and adjustment for multiple comparisons
☐	☒	A full description of the statistical parameters including central tendency (e.g. means) or other basic estimates (e.g. regression coefficient) AND variation (e.g. standard deviation) or associated estimates of uncertainty (e.g. confidence intervals)
☐	☒	For null hypothesis testing, the test statistic (e.g. *F*, *t*, *r*) with confidence intervals, effect sizes, degrees of freedom and *P* value noted<br>*Give P values as exact values whenever suitable.*
☒	☐	For Bayesian analysis, information on the choice of priors and Markov chain Monte Carlo settings
☒	☐	For hierarchical and complex designs, identification of the appropriate level for tests and full reporting of outcomes
☒	☐	Estimates of effect sizes (e.g. Cohen's *d*, Pearson's *r*), indicating how they were calculated

Our web collection on statistics for biologists contains articles on many of the points above.

Software and code

Policy information about availability of computer code

Data collection	- RNA-seq libraries were prepared and sequenced by Biomarker Technologies (BMK) on an Illumina NovaSeq X platform with 150 PE reads.<br>- ChIP-seq libraries were sequenced on an Illumina high-throughput sequencing platform with 150 PE reads.<br>- Confocal images were acquired with a Nikon A1 laser scanning confocal microscope using NIS-Elements software.<br>- Images of ovules stained with Lugol's solution and images of in situ hybridization were captured with an Axiocam MRc5 camera (Zeiss) using Axiovision software (version 4.1).

Data analysis	- For RNA-seq Raw reads were processed using Trimmomatic v0.39, mapped to the TAIR10 reference genome using STAR v2.7.10b, and quantified with featureCounts v2.1.1 using the Araport11 annotation. Count filtering was performed with HTSFilter. Differential expression analysis and principal component analysis were performed in R (RStudio, R v4.4.1) using DESeq2. Venn diagrams were generated with Venny v2.1.0 and KEGG enrichment analysis was performed using ShinyGO v0.81.<br>- For ChIP-seq raw read quality was assessed using FastQC. Adapter trimming was performed with Trimmomatic, reads were aligned to the TAIR10 reference genome using Bowtie2, and peak calling was performed with MACS2. Significant peaks were merged using mergeBED from BEDTools, differential analysis was performed using DESeq2, and peak annotation was carried out using the ChIPseeker R package. Functional enrichment analysis was performed using DAVID and Venn diagrams were generated with Venny v2.1.0.<br>- Raw GC/MS data were processed using MS-DIAL v4.20. Relative abundance values were normalized using QC samples and the internal standard (ribitol), log-transformed and Pareto scaled. Statistical analyses, including one-way ANOVA with Fisher's LSD post hoc test, principal component analysis (PCA), partial least squares discriminant analysis (PLS-DA), permutation testing and heatmap generation, were performed using MetaboAnalyst v4.0.<br>- Confocal images were analysed using Fiji software.<br>- Graphpad Prism 8 awas used for statistical analysis and graphical data presentation.

For manuscripts utilizing custom algorithms or software that are central to the research but not yet described in published literature, software must be made available to editors and reviewers. We strongly encourage code deposition in a community repository (e.g. GitHub). See the Nature Portfolio guidelines for submitting code & software for further information.

Data

Policy information about availability of data

All manuscripts must include a data availability statement. This statement should provide the following information, where applicable:

- Accession codes, unique identifiers, or web links for publicly available datasets
- A description of any restrictions on data availability
- For clinical datasets or third party data, please ensure that the statement adheres to our policy

The RNA-seq raw data generated in this study have been deposited to the NCBI under the accession PRJNA1492831[https://www.ncbi.nlm.nih.gov/bioproject/PRJNA1492831] and PRJNA1492637 [https://www.ncbi.nlm.nih.gov/bioproject/PRJNA1492637]. The ChIP-seq raw data generated in this study have been deposited to the NCBI under the accession PRJNA1491678 [https://www.ncbi.nlm.nih.gov/bioproject/PRJNA1491678]. RNA-seq and ChIP-seq final processed data (list of DEGs and STK target genes) are included in Supplementary Dataset 1, 2, 5, 6. Metabolomic data are included in Supplementary Dataset 4. Source data are provided with this paper. All the materials of the study are available from the Corresponding Author upon request.

Research involving human participants, their data, or biological material

Policy information about studies with human participants or human data. See also policy information about sex, gender (identity/presentation), and sexual orientation and race, ethnicity and racism.

Reporting on sex and gender	*Use the terms sex (biological attribute) and gender (shaped by social and cultural circumstances) carefully in order to avoid confusing both terms. Indicate if findings apply to only one sex or gender; describe whether sex and gender were considered in study design; whether sex and/or gender was determined based on self-reporting or assigned and methods used.*<br>*Provide in the source data disaggregated sex and gender data, where this information has been collected, and if consent has been obtained for sharing of individual-level data; provide overall numbers in this Reporting Summary. Please state if this information has not been collected.*<br>*Report sex- and gender-based analyses where performed, justify reasons for lack of sex- and gender-based analysis.*
Reporting on race, ethnicity, or other socially relevant groupings	*Please specify the socially constructed or socially relevant categorization variable(s) used in your manuscript and explain why they were used. Please note that such variables should not be used as proxies for other socially constructed/relevant variables (for example, race or ethnicity should not be used as a proxy for socioeconomic status).*<br>*Provide clear definitions of the relevant terms used, how they were provided (by the participants/respondents, the researchers, or third parties), and the method(s) used to classify people into the different categories (e.g. self-report, census or administrative data, social media data, etc.)*<br>*Please provide details about how you controlled for confounding variables in your analyses.*
Population characteristics	*Describe the covariate-relevant population characteristics of the human research participants (e.g. age, genotypic information, past and current diagnosis and treatment categories). If you filled out the behavioural & social sciences study design questions and have nothing to add here, write "See above."*
Recruitment	*Describe how participants were recruited. Outline any potential self-selection bias or other biases that may be present and how these are likely to impact results.*
Ethics oversight	*Identify the organization(s) that approved the study protocol.*

Note that full information on the approval of the study protocol must also be provided in the manuscript.

Field-specific reporting

Please select the one below that is the best fit for your research. If you are not sure, read the appropriate sections before making your selection.

☒ Life sciences ☐ Behavioural & social sciences ☐ Ecological, evolutionary & environmental sciences

For a reference copy of the document with all sections, see nature.com/documents/nr-reporting-summary-flat.pdf

Life sciences study design

All studies must disclose on these points even when the disclosure is negative.

Sample size	No sample-size calculation was performed. Sample sizes were chosen based on previous studies and are reported in the Methods section, in the Figure legends, and in Source Data.
Data exclusions	No data were excluded from the analyses.
Replication	RNA-seq experiments were performed on four biological replicates. ChIP-seq experiments were performed on two biological replicates of immunoprecipitated (IP) samples and one input sample. Metabolomic analysis was performed on four biological replicates. For complementation analyses, between two and three independent transgenic lines were analysed All the other experiments were performed on at least three biological replicates.
Randomization	Plants of the same genotype were randomly allocated into experimental groups.
Blinding	Investigators were not blinded during the experiments. To minimize potential bias, experiments were performed independently by different researchers.

Reporting for specific materials, systems and methods

We require information from authors about some types of materials, experimental systems and methods used in many studies. Here, indicate whether each material, system or method listed is relevant to your study. If you are not sure if a list item applies to your research, read the appropriate section before selecting a response.

Materials & experimental systems

n/a	Involved in the study	
☐	☒	Antibodies
☒	☐	Eukaryotic cell lines
☒	☐	Palaeontology and archaeology
☒	☐	Animals and other organisms
☒	☐	Clinical data
☒	☐	Dual use research of concern
☐	☒	Plants

Methods

n/a	Involved in the study	
☐	☒	ChIP-seq
☒	☐	Flow cytometry
☒	☐	MRI-based neuroimaging

Antibodies

Antibodies used	anti-GFP antibody (Abcam, ab290)<br>anti-DIG-AP (Roche, Cat No. 11093274910)
Validation	The commercial anti-GFP antibody (Abcam, ab290) has been validated for ChIP and multiple other applications. Product information and validation data are available at product profile: https://www.abcam.com/en-us/products/primary-antibodies/gfp-antibody-ab290.<br>The commercial anti-Digoxigenin-AP, Fab fragments (Roche, Cat No. 11093274910) has been validated for in situ mRNA hybridization in many published papers. Product information and validation data are available at product profile: https://www.sigmaaldrich.com/IT/it/product/roche/11093274910.

Dual use research of concern

Policy information about [dual use research of concern]

Hazards

Could the accidental, deliberate or reckless misuse of agents or technologies generated in the work, or the application of information presented in the manuscript, pose a threat to:

No	Yes	
x		Public health
x		National security
x		Crops and/or livestock
x		Ecosystems
x		Any other significant area

Experiments of concern

Does the work involve any of these experiments of concern:

No	Yes	
x		Demonstrate how to render a vaccine ineffective
x		Confer resistance to therapeutically useful antibiotics or antiviral agents
x		Enhance the virulence of a pathogen or render a nonpathogen virulent
x		Increase transmissibility of a pathogen
x		Alter the host range of a pathogen
x		Enable evasion of diagnostic/detection modalities
x		Enable the weaponization of a biological agent or toxin
x		Any other potentially harmful combination of experiments and agents

Plants

Seed stocks	Arabidopis Col-0 seed stocks were maintained at University of Milan, Italy. The abs/tt16-6, stk-2, abs/tt16-6 stk-2, pSTK::STK-GFP, pABS::ABS-GFP and pWOX9::WOX9-GFP seeds were previously described and already available at University of Milan or kindly provided by collaborators.
Novel plant genotypes	All the transgenic plants were generated by Agrobacterium-mediated transformation. Multiple T1 plants were obtained from selection, and two or three independent T2 lines were analysed depending on the experiment, as described in the main text.
Authentication	Plant genotypes were tested as follows:<br>- abs/tt16-6, stk-2, abs/tt16-6 stk-2: PCR-based genotyping.<br>- pSTK::STK-GFP, pABS::ABS-GFP, pWOX9::WOX9-GFP: screening for presence of GFP signal by fluorescence microscopy.<br>- pDD22::BAM5, pSTK::BG_PPAP, pSTK::MEE48 lines: selection on germination medium and PCR-based genotyping to test the presence of the construct.

ChIP-seq

Data deposition

- [x] Confirm that both raw and final processed data have been deposited in a public database such as GEO.
- [x] Confirm that you have deposited or provided access to graph files (e.g. BED files) for the called peaks.

Data access links<br>*May remain private before publication.*	https://dataview.ncbi.nlm.nih.gov/object/PRJNA1491678?reviewer=77fcl7o3qbsv5qh614jr2ep3ge
Files in database submission	IP1_pre-fertilization<br>IP2_pre-fertilization<br>Input_pre-fertilization<br>IP1_post-fertilization<br>IP2_post-fertilization<br>Input_post-fertilization
Genome browser session<br>(e.g. UCSC)	https://genome.ucsc.edu/s/Chiara_Astori/ChIPseq_STK

Methodology

Replicates	Two biological replicates of immunoprecipitated (IP) samples and one input sample, as reference, were sequenced.
Sequencing depth	Total reads<br>Input_pre-fertilization: 6277273<br>IP1_pre-fertilization: 8906668<br>IP2_pre-fertilization: 10796483<br>Input_post-fertilization: 7659204

IP1_post-fertilization: 8700086
IP2_post-fertilization: 5951935

Paired aligned one
Input_pre-fertilization: 4201531
IP1_pre-fertilization: 6340900
IP2_pre-fertilization: 7182184
Input_post-fertilization: 5094901
IP1_post-fertilization: 6097140
IP2_post-fertilization: 4036216

Sequence length 50
Paired-end reads

Antibodies

anti-GFP antibody (Abcam, ab290)

Peak calling parameters

For peak calling the program MACS2 was used with parameters callpeak -g 101274395 -q 0.05 -B --mfold 2 20 -f BAMPE

Data quality

The quality of the raw reads was assessed with FastQC. Adapter sequences were removed from the sequencing reads using Trimmomatic and the quality of trimmed reads was reassessed. Only reads with MAPQ score > 40 were kept. During peak calling, MACS2 automatically discarded peaks with a q-value higher that 5.00e-02. Peaks were merged by BEDTools.
This approach retrived a list of 6798 peaks with FDR lower than 5% , including 1549 peaks with fold enrichment > 5.0 (PRE-fertilization samples), and a list of 8230 peaks with FDR lower than 5% , including 2224 peaks with fold enrichment > 5.0 (POST-fertilization samples).

Software

Raw data were trimmed using Trimmomatic 0.39. Mapping was performed using Bowtie 2 (version 2.4). Filtering was performed with SAMtools (version 1.14). Peak calling was performed using MACS2 (version 2.1.1.20160309), and peaks merged with BEDTools (version 2.31). Peak annotation was performed using ChIPseeker, and the GO enrichment analysis was performed with the online tools DAVID.
