# Supplementary material for: ARABIDOPSIS Bsister and SEEDSTICK MADS-box transcription factors modulate maternal nutrient flow for seed development in Arabidopsis
Source: Nat Commun. 2026 Sep 10;17:10687. doi: 10.1038/s41467-026-77573-2 (PMC13655123; doi:10.1038/s41467-026-77573-2)
Supplement: Supplementary file 2 [file 41467_2026_77573_MOESM13_ESM.pdf]

nature portfolio

Peer Review File

ARABIDOPSIS Bsister and SEEDSTICK MADS-box transcription factors modulate maternal nutrient flow for seed development in Arabidopsis

Corresponding Author: Professor Lucia Colombo

This file contains all reviewer reports in order by version, followed by all author rebuttals in order by version.

Version 0:

Reviewer comments:

Reviewer #1

(Remarks to the Author)
This manuscript provides a comprehensive analysis of the mechanisms behind the reduced fertility of the abs stk double mutant. Starting from the observations of starch accumulation in the embryo sac, the authors established through a solid work flow, that ABS and STK regulates genes involved in sugar metabolism and transport, as well as in callose break down at the CHZ region, as to the degradation of sporophytic starch and transport of sugar through the maternal tissue (funiculus and CHZ), via integuments, ending in the embryo to sustain its early growth. It combines microscopic analysis with transcriptomics, metabolomics, and ChIP-seq analysis.

Find some comments about the work:

Concerning the robustness of the observations: There are often a big variation in the number of observations among genotypes, questioning the bias of the input data for the statistical analysis. I wonder whether the authors tested for the normality of the data and whether another non-parametric test would be more appropriate. For examples, Figure 4c n = 5 to 24!!, Figure S6 n = 8-13, etc. Some n in some genotypes are very low compared to others. Ideally, the authors should have obtained observations in a similar amount of samples to obtain robust data.

Concerning Supplementary Fig 5a and Fig 9, why is this Annobar plot so long? Is there not a better to visualise such profile? I was also interesting whether difference in the STK binding pattern profile between samples pre-fertilization and post-fertilization was observed. It may be worth to just mention it either way.

On the same experiments, a Venn diagram comparing the RNAseq (abs stk vs WT), pattern of expression and ChIPseq of STK presented in Supplementary Tables 2 and 6 could be worth adding to the figures. It would illustrate the overlap in the dataset and strengthen the arguments made by the authors on STK's roles.

Also for the robustness of the conclusion of the authors on enhanced callose deposition in the abs stk mutant ovules/seeds, the fluorescence intensity of aniline blue staining in fig 6b should be quantified.

Minor comments:
- On line 97, the expression of STK and ABS in seeds in referred to by citing ref 3 (Coen et al, 2017). However, this article does not show any expression pattern on STK and ABS in seeds. The authors should either provide another reference, amend the text or provide such pictures.
- On lines 290-291, PDLP1 and PDLP2 expression was upregulated in the double mutant compared to wt, but also in stk vs wt, contrary to what the text states.
- Supplemental tables: I would recommend the authors to add one line in teh first sheet of the tables to indicate which table it is, its title and brief legend, as this information is lost as the file name are changed by the submission system. It would be appreciate by the reader when navigating the supplemental files.
- In figure legends: please replace Tuckey by Tukey.
- On line 247: replace "with comprised nutrient flow" by "with the hypothesis of a comprised..."

Reviewer #2

(Remarks to the Author)
The manuscript entitled “ARABIDOPSIS Bsister and SEEDSTICK MADS-box transcription factors modulate maternal nutrient flow for seed development in Arabidopsis” by Banfi et al. showed that MADS domain transcriptional factors ABS and STK play critical roles in regulating nutrient flow during ovule maturation and seed development. They combined RNA-seq, metabolomics, ChIP-seq analyses to dissect the nutrient allocation in ovule and seed development and provide new insights into this research field. The manuscript is well-written, easy to follow, but indeed there are several aspects need further improvements to meet the requirements of Nature Communications. Please check the comments below:

Major issues：
1. The introduction is not well-structured, the background is not clear enough, I would encourage the author to expand the content. For example, there are several well-known plant sugar transporters, including SUTs/SUCs, MSTs and SWEET, key findings related to these sugar transporters should be mentioned in the introduction section. Also, the author should mention the difference from long-distance movement and phloem loading.
2. Figure 1a, the developmental stage of the stk mutant is not the same with others, it’s obviously immature ovule. Please double check the result.
3. Figure 1b, the difference between WT and double mutant is clear, while it’s hard to identify the difference between abs or stk single mutant to the bas stk double mutant. Please clearly describe and mark the pronounce of nucellus in the figure and result section. Also the statistical analysis in Figure 1c is not convincing, as the author only used 8 seeds for this analysis. Please increase the number of seeds and clearly describe their origin, like from how many siliques.
4. The expression pattern analysis of STK and ABS should extend to at least 2DAP, I think it’s meaningful to check their expression pattern at the stage when nucellus difference can be clearly spotted (like the stage in Figure 1b).
5. The author collected pistil sample for RNA-seq analysis, this triggers my concern for the dilution effect of expression pattern analysis. As seen in Figure 2c, the fold change of gene expression is relatively not obvious, which may be related to sample collection. Although the author performed in situ hybridization to confirm the expression pattern change in double mutant and WT, but the robustness and reliability of in situ hybridization are not convincing enough. I would suggest the author use fluorescent marker lines of several candidates and perform time-lapse/sequential observation of their expression pattern during ovule development and after fertilization. This will significantly improve the quality of data and reliability of the conclusion.
6. The author claimed that “ABS appears to be a key factor limiting accumulation of soluble sugars in young flowers, whereas STK tends to promote their accumulation” in the manuscript, which is not well-supported. Just based on the metabolomic analysis result and give such conclusion is not sufficient, not to mention that the author also concludes that “abs is epistatic to stk in terms of sucrose, glucose 6-phosphate and fructose 6-phosphate accumulation”. To make such conclusion, solid genetic evidence is absolutely required.
7. In Figure 4a, the author tried to claim the starch accumulation in ovule by Lugol staining, I would like to draw their attention to sampling stage, as it would severely interfered with the result. In principle, all ovule should be at the same stage, and the staining result should be quantified to support the conclusion.
8. The author ectopically expressed BAM5 in central cell to observe the starch accumulation in female gametophyte. There are several concerns regarding this experimental design: first, only BAM5 can not fully represent starch metabolism, other starch-related genes/candidates should also be included; second, please consider the promoter being use here, at least explain clearly the rationale of ectopically expressed the genes in central cell.
9. The result section regarding to callose turnover is only descriptive, based only on these observations, the conclusion at the end of this section is way too speculative. The author should either provide more direct evidence to support this conclusion (which is recommended), or at least tone down the conclusion (but this will definitely reduce the importance of this phenomenon).
10. I have general concern for the sample selection of RNA-seq in the manuscript. Using silique is simple, but may not be perfect to reflect the precise change that the authors are interested. This is not easy to modify, that I am truly aware, so the author needs to perform extra experiments to confirm it, like the what I commented on Figure 2c, also applies to Figure 7. Of course, not the same experiments are required, but further confirmation is definitely needed.
11. The model is quite clear, but to support the blocking effect, please provide extra experimental evidence (genetic investigation would be the best), for example, if the author could artificially block the callose degradation at the phloem end gate and the similar effects can be observed, that will be fantastic.

Minor issues:
1. Staining result should be quantified, also in situ hybridization and other staining.
2. Supplementary Figure 6c, please provide figures with better quality, in this version, it’s difficult to see the difference.
3. The author could also consider Whole amount in situ hybridization instead of sectioned samples, which might be more easy to see the difference and more reliable.

Reviewer #3

(Remarks to the Author)
The manuscript describes and interesting, well-planned and well-executed study. The authors have combined their experience and skills in analysis of ovule structure and ovule development mutations with recent results on the importance of nutrient transport into fertilized ovules for seed filling/growth (e. g. Liu, X. et al., 2025) to extend these studies.
They first show that there is a synergistic effect of a combination of the abs and stk mutations on ovule morphology and anatomy, detailing these effects in a series of excellent micrographs in Fig. 1. They further confirm prior results that the

double mutant accumulates a higher level of starch than wild type, and this was previously shown to persist following fertilization, in contrast to wild type where the starch decreases dramatically.
Using functional genes producing GFP fusion proteins they show that ABS and STK have complementary non-overlapping expression patterns in ovules. This partly contradicts prior work from this group (Misszzotti et al, 2012) where in situ hybridization showed similar complementary expression patterns, except that there was overlapping signal in the ovule endothelium. Endothelium expression is not visible for either gene here. This could be a difference in protein accumulation vs. mRNA accumulation, or a result of the transgene analysis. Since the transgenes are functional, if there is mRNA production in the endothelium it is not functionally relevant.
Transcriptomics were used to test for changes in gene expression in the mutant lines. No additive effect on DEGs were observed for the two mutations combined. But this may not be meaningful since the RNA was isolated from complete gynoecia and the genes appear to affect only specific tissue of the ovules so effects on specific regions would not be observed. Still, these studies provide information on candidate genes for regulation that could affect starch metabolism. Figure 2 and Supplementary Figure 1 display analysis of these data and are clear and informative. The authors also cite Supplementary Tables 1 and 2, but these are not tables, but multi-tab Excel files of data and analysis of data. They provide support for the figures, but are not nearly as simple to interpret as would be tables summarizing the data.
They tested expression of two genes, TRE1 and BAM4 by examining in situ hybridization results. These results appeared to confirm the lower expression of both genes. This is important because this shows that the downregulation is manifest in ovules, rather than in other pistil tissues included in the RNA-seq analysis.
Metabolomics was applied to detect observable effects on starch metabolism that could be associated with the persistence of starch in the double mutant. Figure 3a and supplementary figures 3 and 4 provide clear informative displays of the most relevant results of this analysis. Supplementary Table 3 is an Excel datafile of the results that backs up these figures, but is again, not a table. The results show clear changes in levels of important intermediates in starch metabolism between the double mutant and both wild-type and single mutant lines. In figure 3b they combine the results of the transcriptomic, metabolomic, and starch staining results to illustrate how the changes in gene expression correlate with the changes in metabolite level in a pattern that could explain the starch accumulation/persistence in the double mutant. The illustration of the results on the different DEGs with the changes in carbohydrates is compelling. This is a clear, informative figure. The only change I would suggest is that the change in starch level is not reflected in the diagram. While it was not the metabolomic results that indicated the elevated starch, I would still add a red up arrow to the starch oval to indicate that it is also elevated as this would make it more clear how the other changes relate to starch. (Unless I have missed something as to why this should not be done).
ChIP-seq was used to see if the subject DEGs might be directly regulated by STK. About one is six genes in the Arabidopsis genome were found to be bound by STK. These include genes in carbohydrate metabolism and transport. Examination of the information in the dataset (referred to as Supplementary table 4) I found that many of the genes that were focused on in the earlier sections were included in the regulated list (notable genes such as BAM4 and TRE1). Some mention of these genes that were focused on earlier in the text would help the manuscript as sorting through the large dataset in supplementary material is significant work for a reader. The results confirm that it is possible that genes important in starch accumulation and degradation could be direct targets of STK regulation.
Use of a central cell-expressing transgene producing an isozyme of a starch degrading enzyme was an excellent test of the hypothesis that the persistent starch of the double mutant played a role in the mutant phenotypic effects. The transgene was shown not only to reduce the CC starch, but to at least partially reverse all of the phenotypic effects of the double mutant including cell divisions, embryo development and viable seed formation. Thus, the aberrant persistence of the starch and failure to release its products is an important part of the effect of the double mutant.
Callose accumulation in the chalaza has previously been shown to constitute a reversible block to seed development that is normally reversed by fertilization. A transported fluorescent tracer was used to confirm the callose block that is reversed by fertilization in wild-type and the two single mutants. However, this block persisted following fertilization in the double mutant. The association of these changes in permeability with callose quantity was confirmed by direct staining for callose which showed that callose removal was decreased in the chalaza of the double mutant. This continued interruption of nutrient flow in the double mutant could explain why the transgene promoting starch mobilization was not sufficient to fully restore seed formation in the double mutant.
The authors applied RNA-seq and ChIP-seq to examine regulation of callose degradation and sugar transport. They found results consistent with ABS and STK promoting expression of genes involved in callose metabolism. A case for modulation of sugar transport was less strong. The model they propose for regulation of nutrient flow into the fertilized ovule is supported by their data.
Overall the authors have used an impressive array of approaches to identify and characterize the likely pathway for the effects on development and fertility in the abs stk double mutant. Rather than being a direct regulation of anatomical features (e. g. the additional cell layer in the integument or development of the endothelium), they are shown to be derivative effects of nutrient restriction in the fertilized ovule. This restriction is the result of a combination of failure in mobilization of nutrients from stored starch, and a failure to remove the chalazal callose that restricts nutrient flow from the funiculus. Hence the fertilized ovules behave more like unfertilized ovules and fail to develop. Their work extends other recent work on nutrient transport into ovules with important regulatory information. The manuscript is clear, well-organized and well-written.
The methods section appears sufficient to enable reproduction of the presented work.
I have only a small number of specific comments for improvement of the manuscript.
1) line 177, I would say "mis-regulation" instead of "deregulation" because both lower and higher expression are observed.
2) The text refers multiple times to Supplementary Tables. The initial version of the manuscript file page did not have any supplementary tables listed. When I asked the publisher for correction, the names of the supplementary data files were changed to Supplementary Table X etc. But these files don't contain tables, but rather are multi-tab excel data files. These files should be referred to as "Supplementary Datafile X". If they want supplemental tables, then they should prepare them (some would actually be useful if they prepared summaries from the supplemental data files of the most pertinent genes). In addition, the text refers to "Supplementary Table 8" which appears in the list of files on the manuscript page at the publisher,

but does not appear on the list of Supplementary Tables at the end of the manuscript. Also in the set of files on the manuscript page at the publisher there is a “Supplementary Table 9” that also does not appear in the list of supplementary information nor anywhere else in the manuscript. This all should be cleared up prior to publication.
3) The results show that it appears to be the lack of carbohydrates released from starch degradation, rather than the presence of starch in the ovule that prevents development. So perhaps refer more to starch mobilization that to starch degradation in the text to emphasize this point. E. g. line 386 they refer to “reducing the starch in the embryo sac” when in fact it is probably the release of degradation products that is the more important aspect of this process.

Version 1:

Reviewer comments:

Reviewer #1

(Remarks to the Author)
The authors did a great job in integrating my (and the ones of the other reviewers) comments into this revised version of the manuscript. This is a great piece of work.

I only have three textual remarks:
line 76: What is STP?

Line 223: "122 out of 135 metabolites significantly differed between the wt and at least one of the three mutants ". I think you meant that their amount significantly differed?

Line 268: "the development of abs stk embryos was delayed". To reflect on this observation, the seed set in the double mutant is reduced. One would have expected that the opposite trend (faster development) as (more) resources are available for less seeds. Yet the development is delayed and seeds are smaller. So, is this delay really because of carbohydrate access? Also, less seeds per silique may bias the measurement.

Reviewer #2

(Remarks to the Author)
After evaluating the revised manuscript and the authors response, I am basically satisfied that all my previous concerns have been addressed properly. The authors have done additional experiments and analyses, which strengthened the manuscript. Although the genetic analysis is still missing, I hope the authors can make careful interpretation and be aware of this weakness.

Reviewer #3

(Remarks to the Author)
The authors have fully addressed my concerns on the original manuscript through their revisions. The addition of tables I and II to the supplementary information are especially appreciated. My reading of the responses to the other reviewers indicates further improvement of the manuscript.
I have no additional suggestions.

Open Access This Peer Review File is licensed under a Creative Commons Attribution 4.0 International License, which permits use, sharing, adaptation, distribution and reproduction in any medium or format, as long as you give appropriate credit to the original author(s) and the source, provide a link to the Creative Commons license, and indicate if changes were made.
In cases where reviewers are anonymous, credit should be given to 'Anonymous Referee' and the source.
The images or other third party material in this Peer Review File are included in the article's Creative Commons license, unless indicated otherwise in a credit line to the material. If material is not included in the article's Creative Commons license and your intended use is not permitted by statutory regulation or exceeds the permitted use, you will need to obtain permission directly from the copyright holder.
To view a copy of this license, visit https://creativecommons.org/licenses/by/4.0/

RESPONSE TO REVIEWER COMMENTS

Reviewer #1 (Remarks to the Author):

This manuscript provides a comprehensive analysis of the mechanisms behind the reduced fertility of the abs stk double mutant. Starting from the observations of starch accumulation in the embryo sac, the authors established through a solid work flow, that ABS and STK regulates genes involved in sugar metabolism and transport, as well as in callose break down at the CHZ region, as to the degradation of sporophytic starch and transport of sugar through the maternal tissue (funiculus and CHZ), via integuments, ending in the embryo to sustain its early growth. It combines microscopic analysis with transcriptomics, metabolomics, and ChIP-seq analysis.

Find some comments about the work:

Concerning the robustness of the observations: There are often a big variation in the number of observations among genotypes, questioning the bias of the input data for the statistical analysis. I wonder whether the authors tested for the normality of the data and whether another non-parametric test would be more appropriate. For examples, Figure 4c n = 5 to 24!!, Figure S6 n = 8-13, etc. Some n in some genotypes are very low compared to others. Ideally, the authors should have obtained observations in a similar amount of samples to obtain robust data.

1. We thank the reviewer for the comment. As suggested by the reviewer, we have increased the number of observations (n) in the experiments described in Figure 1c, 4c (renamed Figure 5c), 5b (renamed Figure 6b), and Supplementary Figure 6a (renamed Supplementary Figure 7a) to obtain an equal number of observations among the genotypes in the frame of each experiment. The new values are in the updated version of the Source Data file. The statistic was conducted using one-way ANOVA or nested ANOVA followed by Tukey HSD ($p < 0.05$). Additionally, we have updated the graphs throughout the manuscript to better illustrate the variability of individual measurements.

Figure 1c:

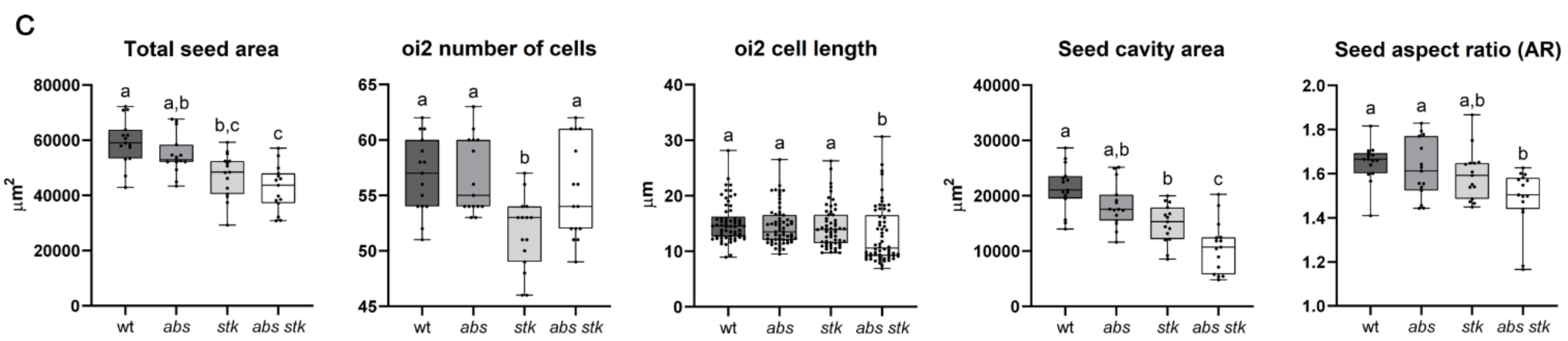

Figure 5c:

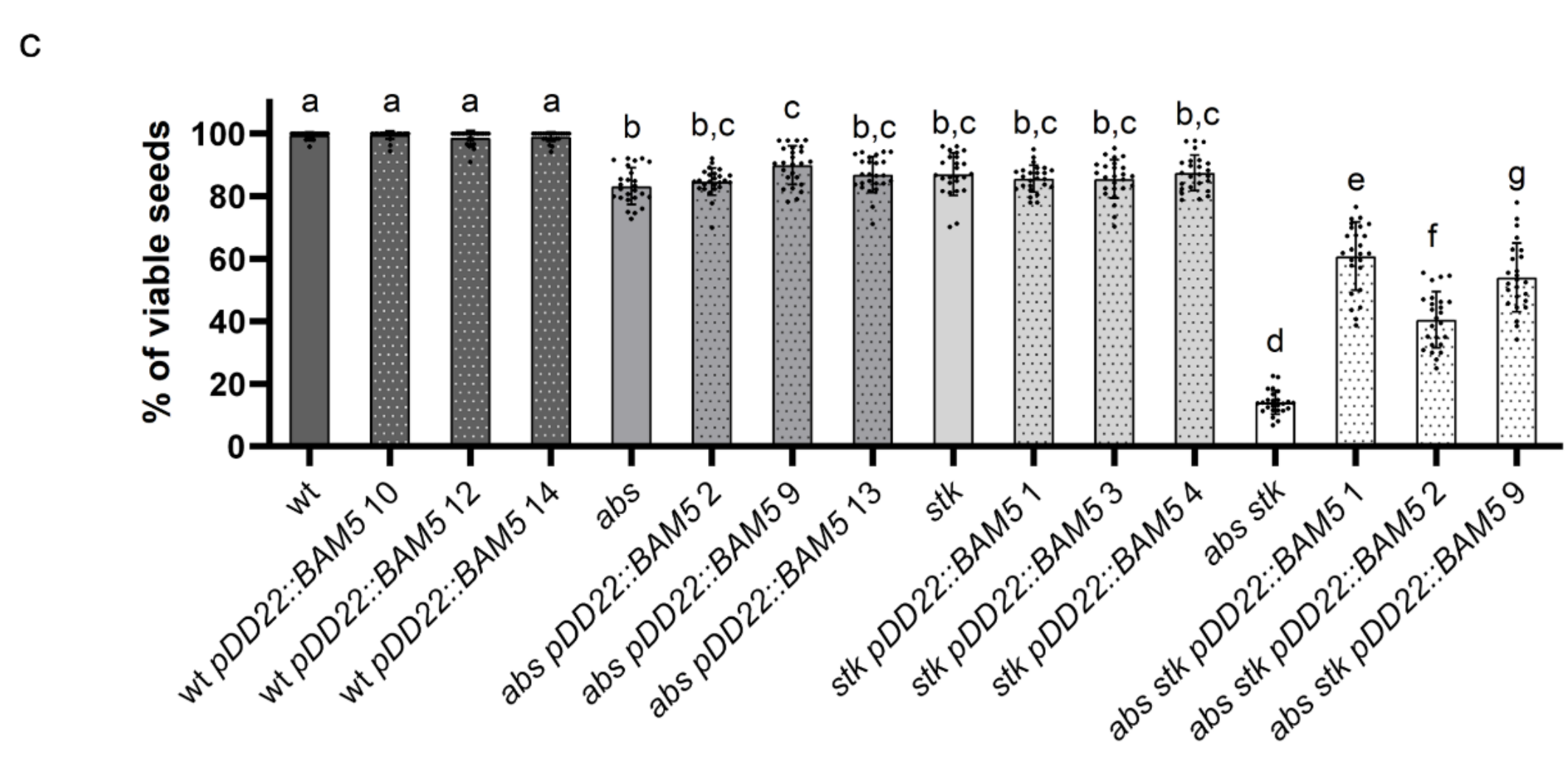

Figure 6b:

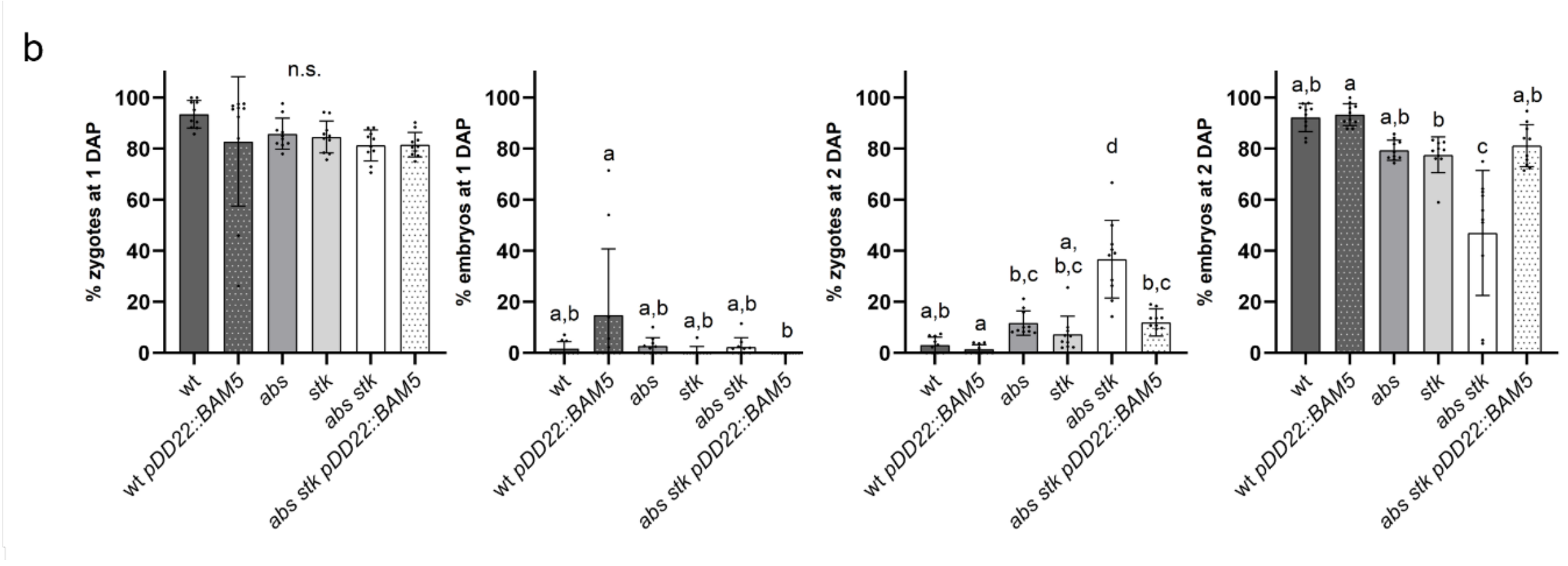

Supplementary Figure 7a:

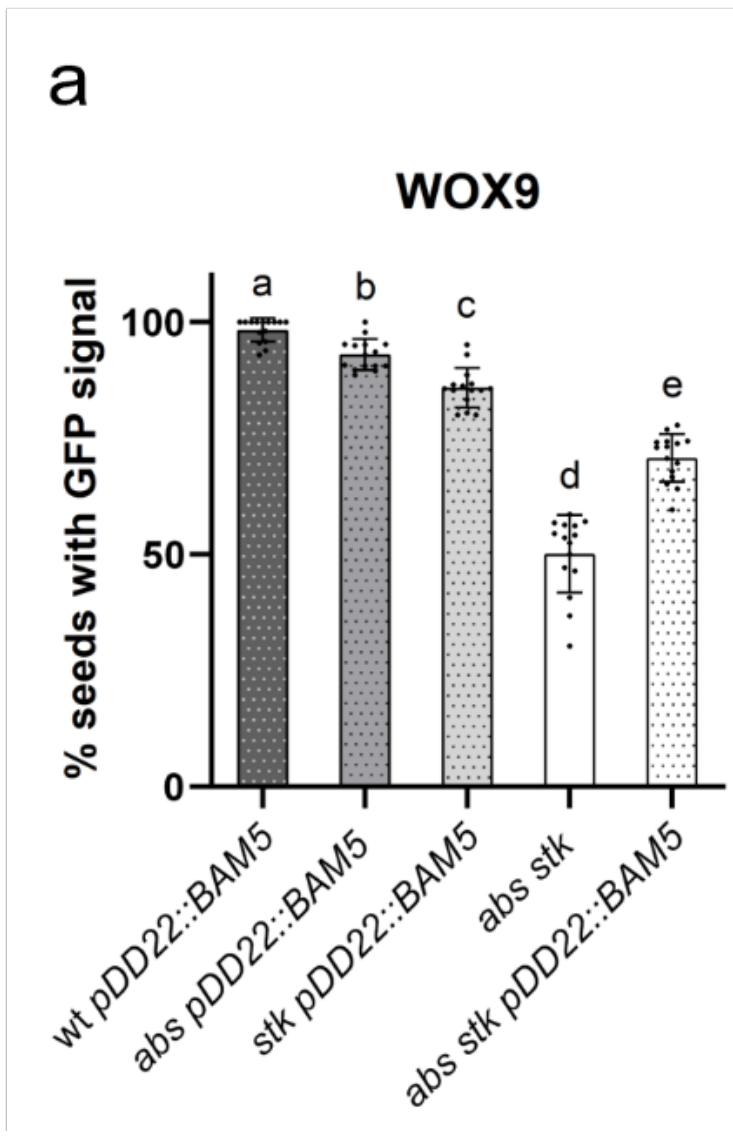

Concerning Supplementary Fig 5a and Fig 9, why is this Annobar plot so long? Is there not a better

to visualise such profile? I was also interesting whether difference in the STK binding pattern profile between samples pre-fertilization and post-fertilization was observed. It may be worth to just mention it either way.

2. We thank the reviewer for the suggestion. We have reduced the height of the Annobar plots in Supplementary Figure 5a (renamed Supplementary Figure 4) and Supplementary Figure 9 (renamed Supplementary Figure 12a) according to the reviewer's comment.

We did not observe substantial differences in the STK binding profile between pre- and post-fertilization samples as regards the percentage of STK peaks in the different regions of the genome. This is well represented by the two AnnoBar plots, which are very similar (compare Supplementary Figure 4 with Supplementary Figure 12a). We have added a sentence in the text to comment on this (line 358-360).

Supplementary Figure 4:

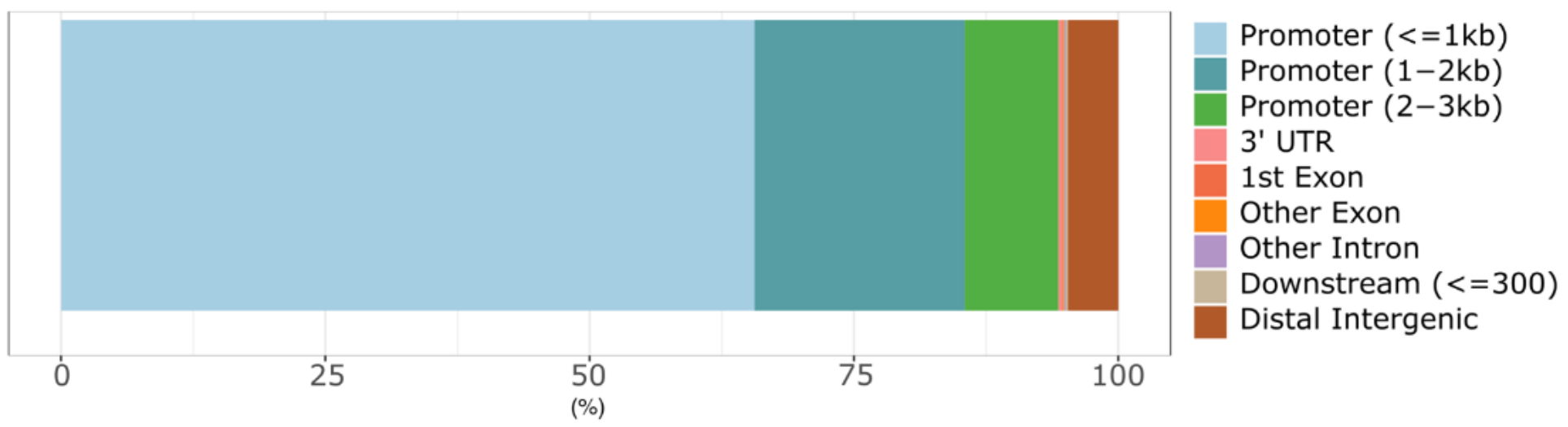

Supplementary Figure 12a:

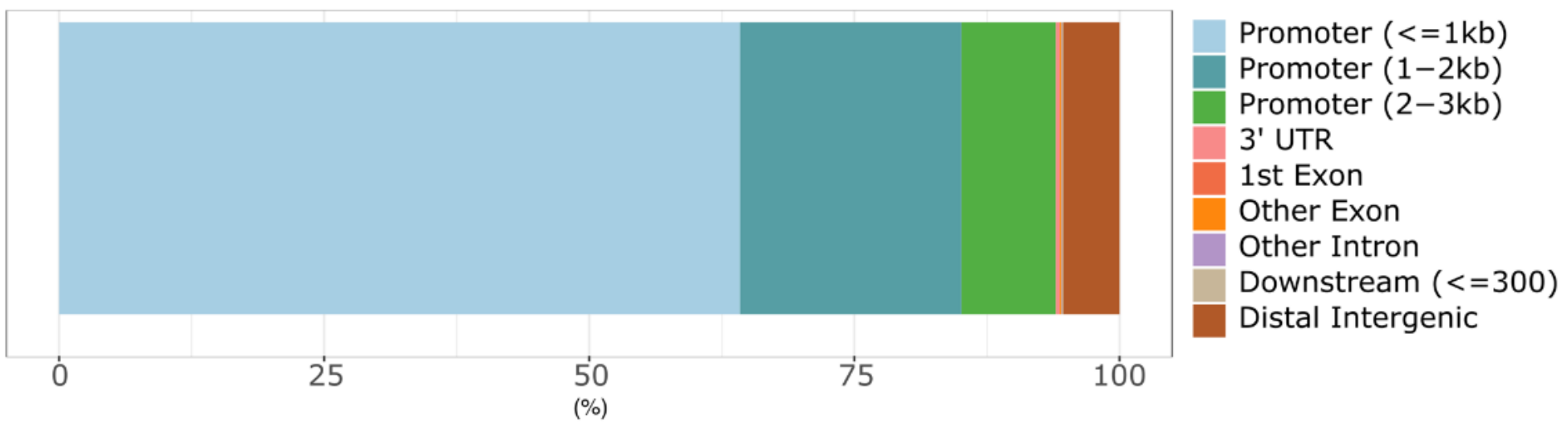

As suggested by Reviewer 1, to clearly show the differences in STK target genes before and after fertilization, we have added a comparison between the two ChIP-seq datasets (Supplementary Dataset 7); this comparison is represented by the Venn Diagram in Supplementary Figure 12b. The diagram indicates that the majority of STK targets are shared between pre- and post-fertilization samples (4382 genes), although there are several genes targeted by STK only in pre- (1103) or post-fertilization (1905) stages. We have added a description in the text, line 360-362.

Supplementary Figure 12b:

b

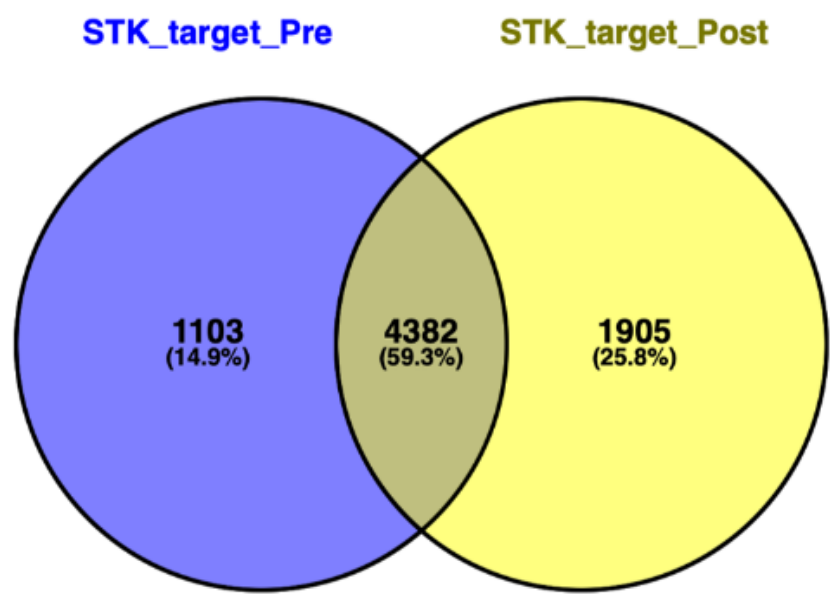

Furthermore, to highlight the regions of the STK binding site for the genes mentioned in the text, we also added images for ChIP-seq peak visualization in pre- and post-fertilization samples (Figure 3b and Figure 8d, respectively). We specifically represented the genes involved in starch and sucrose metabolism (pre- fertilization) and callose turnover, sugar transport and auxin response (post- fertilization) that are differentially expressed between *abs stk* mutant and wt in pre- and post-fertilization RNA-seq data. We described this in the Result section, line 208-212 and 362-372, respectively.

On the same experiments, a Venn diagram comparing the RNAseq (abs stk vs WT), pattern of expression and ChIPseq of STK presented in Supplementary Tables 2 and 6 could be worth adding to the figures. It would illustrate the overlap in the dataset and strengthen the arguments made by the authors on STK's roles.

3. We thank the reviewer for the suggestion. We have added two Venn diagrams comparing the RNA-seq (*abs stk* vs wt comparison) and STK ChIP-seq data in pre- and post-fertilization samples (Figure 3a and Figure 8c, respectively). The diagrams show that in pre-fertilization samples, only the 18% of genes are both STK direct targets and deregulated in *abs stk* to wt comparison (768 downregulated, 1025 upregulated), while in post-fertilization samples, the 20% of STK targets are also differentially expressed in the *abs stk* to wt comparison (1097 downregulated, 1436 upregulated). This suggests that although STK directly regulates many genes, several of the differentially expressed genes in the *abs stk* mutant compared to wt are likely not direct STK targets, or they might be ABS targets.

Figure 3a:

a

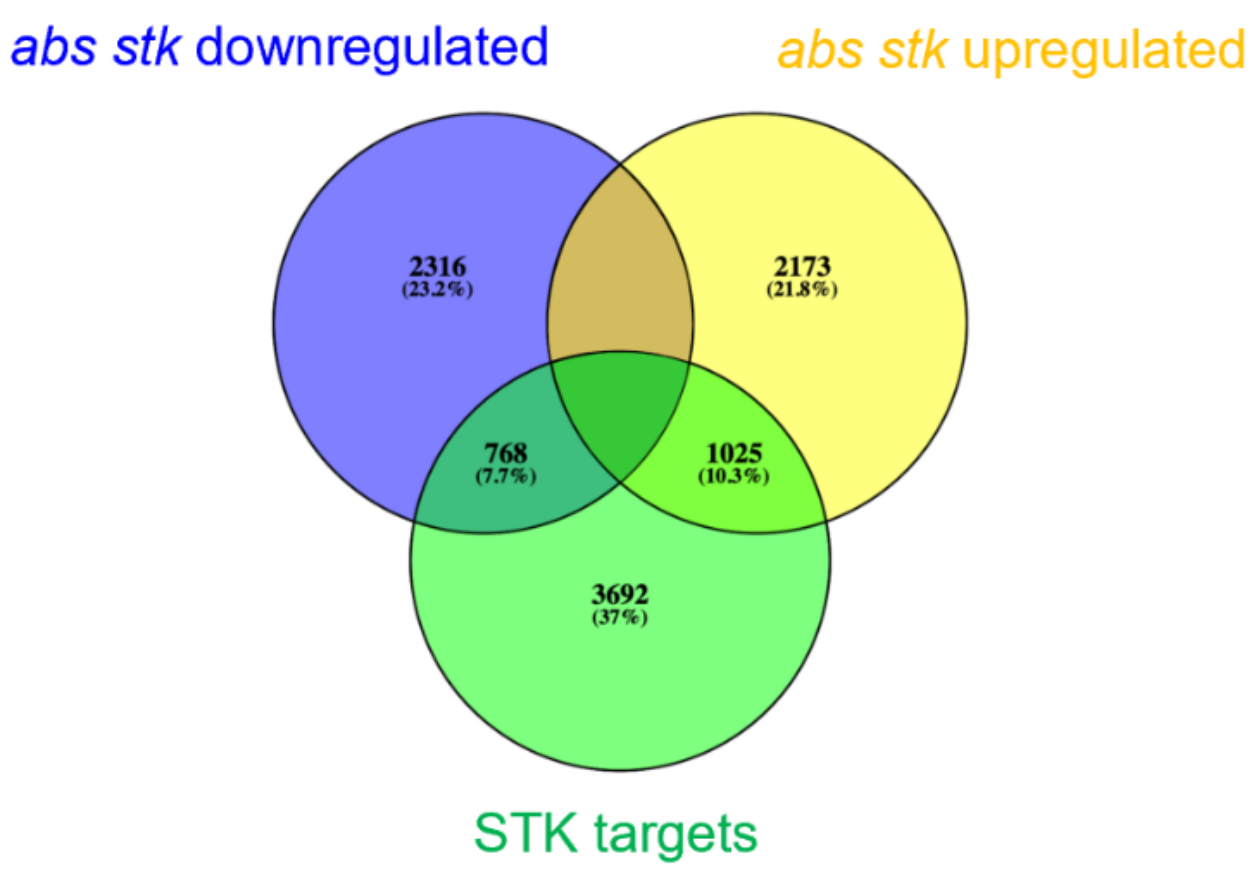

Figure 8c:

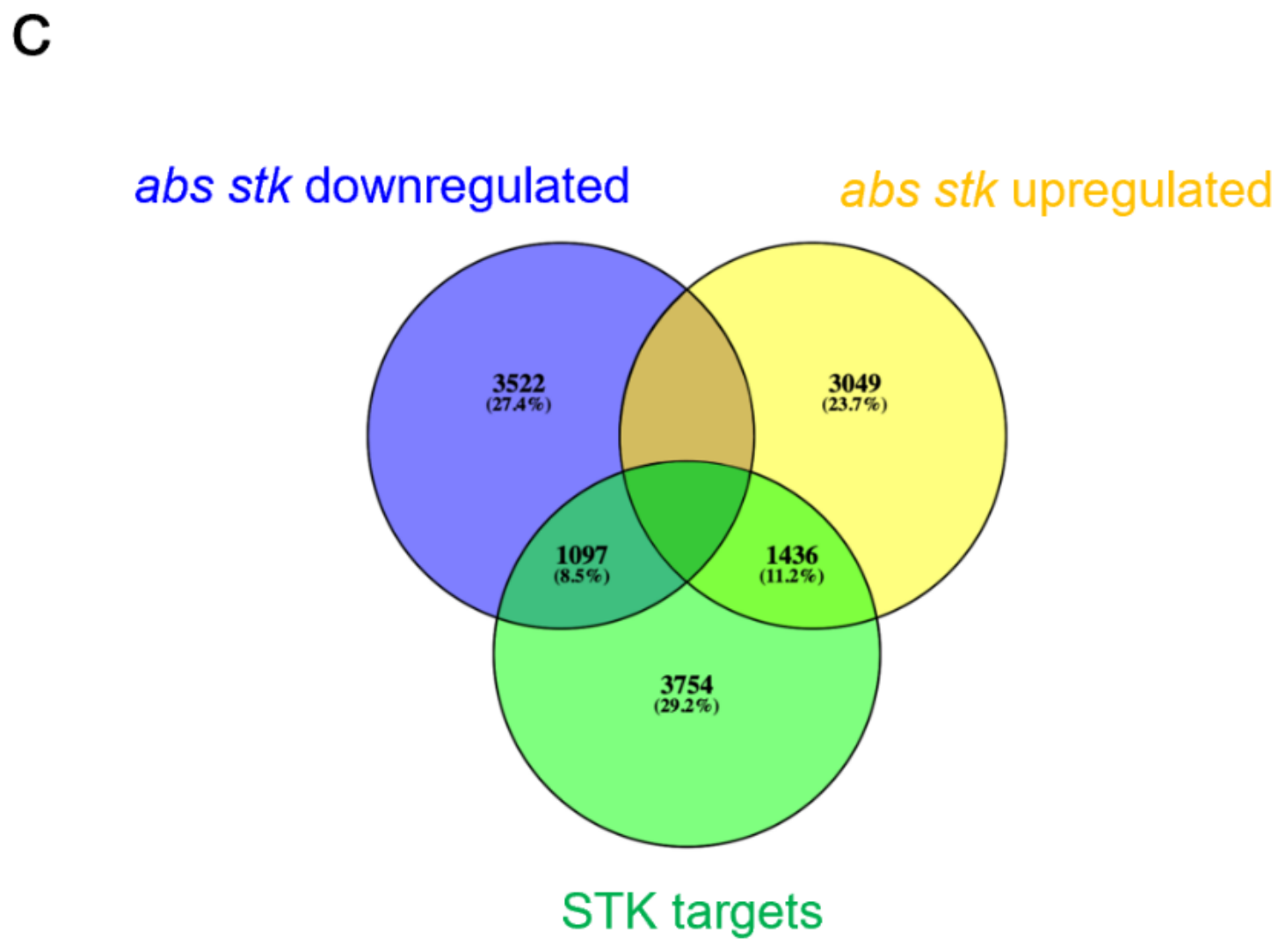

Also for the robustness of the conclusion of the authors on enhanced callose deposition in the abs stk mutant ovules/seeds, the fluorescence intensity of aniline blue staining in fig 6b should be quantified.

4. We thank the reviewer for the suggestion. As suggested, we have quantified callose at the ovule/seed chalazal region by measuring the fluorescence of aniline blue staining by using Fiji software, confirming our previous observations (Figure 7c and d, Supplementary Figure 9, and Source Data). In particular, we have calculated the CTCF (Corrected Total Cell Fluorescence), which represents the fluorescence intensity of a certain area (Region Of Interest, ROI) corrected by subtraction of the background. The ROI (highlighted in yellow in Figure 7d) was selected based on the aniline blue signal in the chalazal region, where the callose gate is located (Liu *et al.*, 2025). We also measured the dimension of this area. In *abs stk* mutant, we detected a higher fluorescence intensity of aniline blue and a larger area occupied by aniline blue signal, in either 1 DAE ovules and 3 DAP seeds, compared to wt. This indicates that in the *abs stk* double mutant more callose is accumulated at the chalaza of ovules and seeds, likely in more cells, and is not degraded after fertilization. No difference was observed between *abs stk* and *abs stk pDD22::BAM5* plants, which correlates with CFDA tracer accumulation (Figure 7a). Interestingly, an intermediate value of fluorescence intensity between wt and *abs stk* was observed for *stk* mutant ovules at 1 DAE. However, at 3 DAP the callose presence in *stk* is similar to wt. We described these results in the text, line 298-307.

Figure 7:

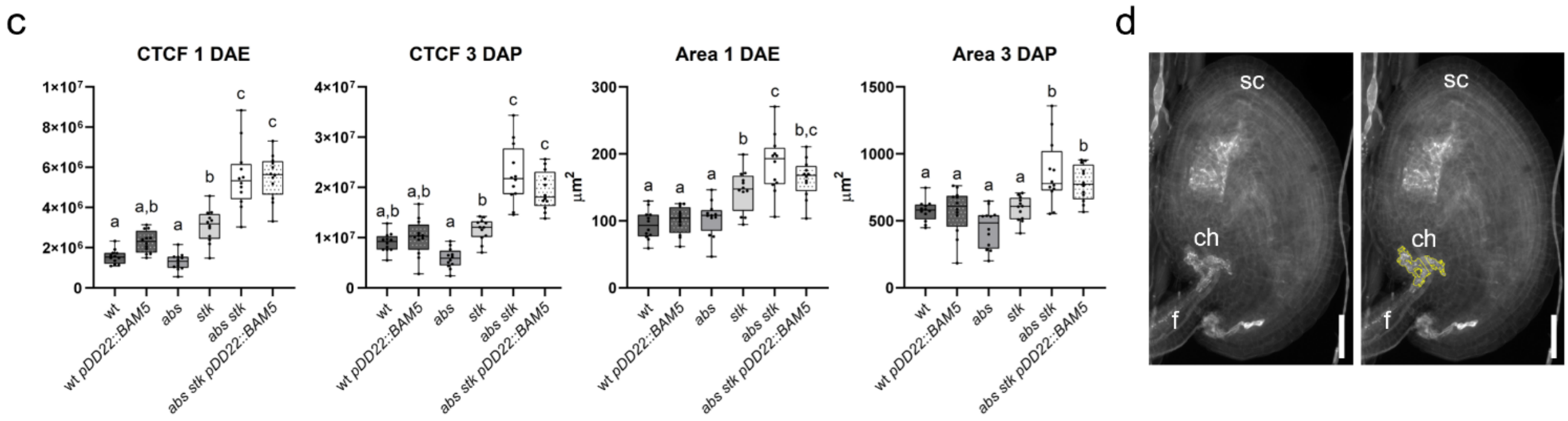

Minor comments:

- On line 97, the expression of STK and ABS in seeds in referred to by citing ref 3 (Coen et al, 2017). However, this article does not show any expression pattern on STK and ABS in seeds. The authors should either provide another reference, amend the text or provide such pictures.

We thank the reviewer for the comment, and we apologise for the mistake. The expression pattern of ABS (*3.4ProTT16:gTT16-GFP*) in seeds is shown in Coen et al., 2017 (Figure 3c), whereas the expression pattern of STK (*pSTK::STK-GFP*) is shown in Mizzotti et al., 2014. Nevertheless, because this work also investigates the role of STK and ABS during seed development, we have included new images illustrating STK-GFP and ABS-GFP expression patterns in seeds at 1, 2 and 3 DAP (Figure 1d and Supplementary Figure 1), and we have described them in the text (line 133-136). In seeds until 3 DAP, the ABS protein localization domain is restricted to the two innermost layers of the seed coat, and in the nucellus, whereas STK-GFP is localized in the three outermost layers, in the chalazal seed coat and in the funiculus. As for pre-fertilization stages, the expression domains of the two fusion proteins do not overlap.

- On lines 290-291, PDLP1 and PDLP2 expression was upregulated in the double mutant compared to wt, but also in stk vs wt, contrary to what the text states.

We thank the reviewer for the comment but, as it was shown in Figure 7b (renamed Figure 8a), *PDLP1* and *PDLP2* are exclusively differentially expressed in wt to *abs stk* comparison.

Figure 8a:

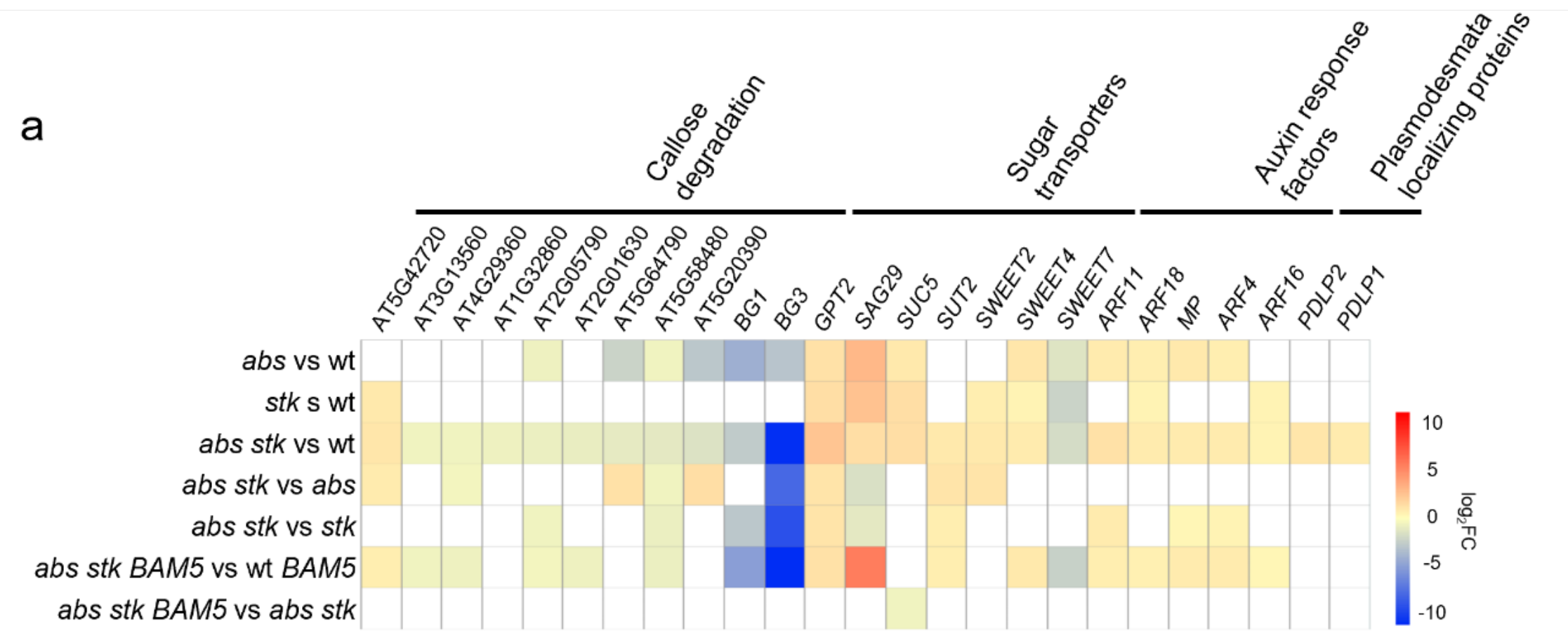

- Supplemental tables: I would recommend the authors to add one line in teh first sheet of the tables

to indicate which table it is, its title and brief legend, as this information is lost as the file name are changed by the submission system. It would be appreciate by the reader when navigating the supplemental files.

We thank the reviewer for the suggestion. We have added this information to all the multi-tab excel files (Supplementary Tables), which have been renamed to Supplementary Datasets as suggested by Reviewer 3.

- In figure legends: please replace Tuckey by Tukey.

We have corrected the text as suggested by the reviewer.

- On line 247: replace "with comprised nutrient flow" by "with the hypothesis of a comprised..."

We have changed the text as suggested by the Reviewer 1 (line 293).

Reviewer #2 (Remarks to the Author):

The manuscript entitled “ARABIDOPSIS Bsister and SEEDSTICK MADS-box transcription factors modulate maternal nutrient flow for seed development in Arabidopsis” by Banfi et al. showed that MADS domain transcriptional factors ABS and STK play critical roles in regulating nutrient flow during ovule maturation and seed development. They combined RNA-seq, metabolomics, ChIP-seq analyses to dissect the nutrient allocation in ovule and seed development and provide new insights into this research field. The manuscript is well-written, easy to follow, but indeed there are several aspects need further improvements to meet the requirements of Nature Communications. Please check the comments below:

Major issues：
1. The introduction is not well-structured, the background is not clear enough, I would encourage the author to expand the content. For example, there are several well-known plant sugar transporters, including SUTs/SUCs, MSTs and SWEET, key findings related to these sugar transporters should be mentioned in the introduction section. Also, the author should mention the difference from long-distance movement and phloem loading.

We thank the reviewer for the comment, and we apologise for the lack of clarity in the introduction. The reviewer's suggestions have been incorporated into the text to both expand and clarify the introduction.

2. Figure 1a, the developmental stage of the stk mutant is not the same with others, it’s obviously an immature ovule. Please double check the result.

We thank the reviewer for the comment. All the pictures of mature-staged ovules (Figures 1a, 4a, and 4b, which have been renamed as Figure 1a, 5a, and 5b, respectively) were taken from pistils at stage 12c (Smyth *et al.*, 1990), ensuring that all ovules were at the same developmental stage (3-VI); this has been now specified in the Methods section. However, it is possible that some ovules may have subtle developmental differences, also due to the genetic background. We have verified the stages to check that the female gametophyte was indeed at the stage 3-VI (see also response 7 to

Reviewer 2). To more accurately represent a *stk* mature stage ovule, we have changed the previous *stk* image (Figure 1a) with a new one. See also response 7 to Reviewer 2.

Figure 1a:

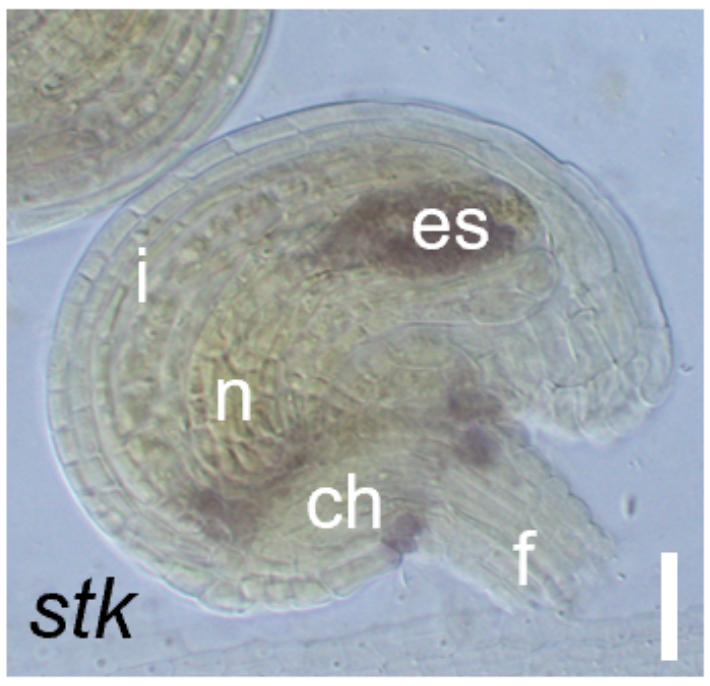

3. Figure 1b, the difference between WT and double mutant is clear, while it's hard to identify the difference between abs or stk single mutant to the bas stk double mutant. Please clearly describe and mark the pronounce of nucellus in the figure and result section. Also the statistical analysis in Figure 1c is not convincing, as the author only used 8 seeds for this analysis. Please increase the number of seeds and clearly describe their origin, like from how many siliques.

We apologise for the lack of clarity. As suggested by Reviewer 2, we have marked in Figure 1b the different seed tissues (nucellus, seed cavity, outer integument 2) with different colours. In particular, the persistence of the nucellus, which is evident in the *abs* and *abs stk* seeds, is now more clearly highlighted in the figure (yellow). Furthermore, it is also evident that *abs stk* has reduced seed cavity with respect to the wt and the single mutants, and reduced seed size. We have also replaced the original images with more representative ones and expanded the description of the phenotypic differences among genotypes in the Results section, line 116-124.

Figure 1b:

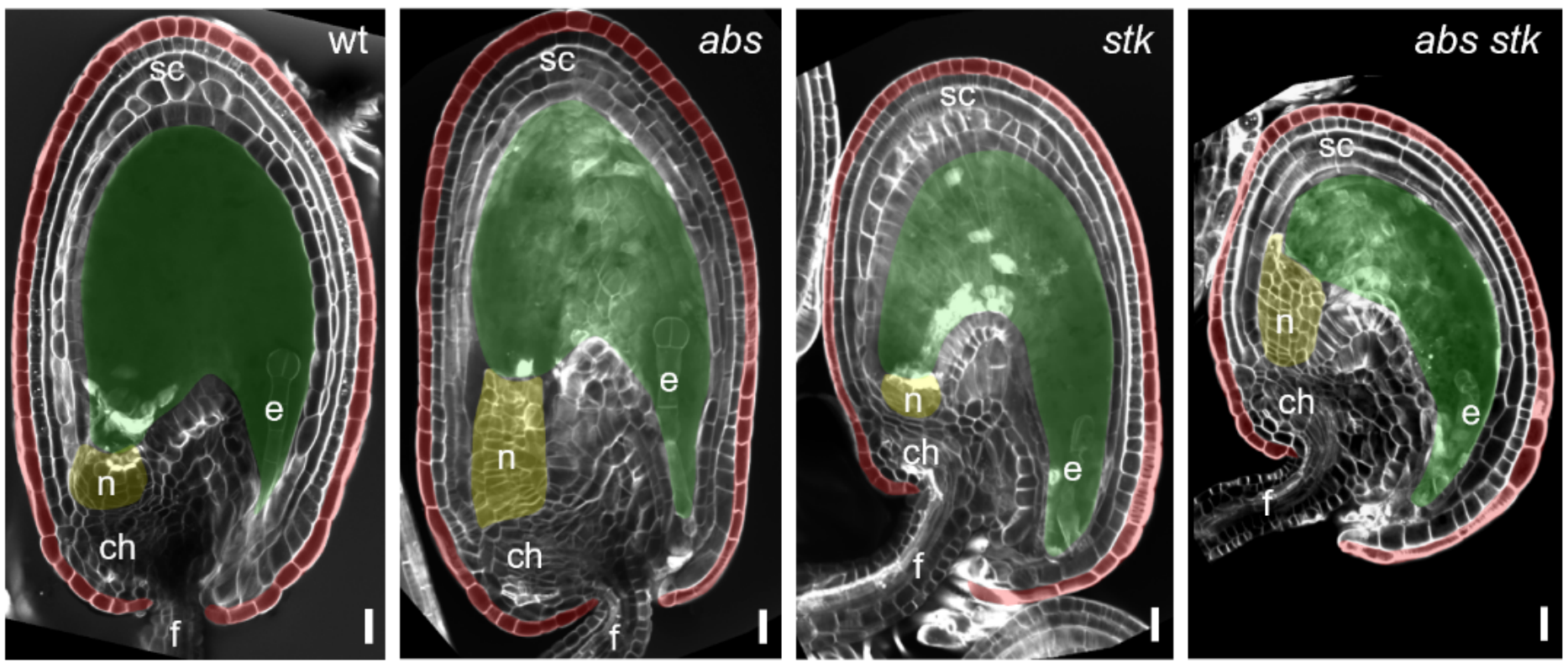

To better characterize the *abs stk* seed phenotype and the differences with respect to single mutants, we complemented the original measurements of oi2 cell number and cell length with additional measurements of the total seed area, the seed cavity area, and seed aspect ratio (Figure 1c and Source Data). *abs stk* seeds at 2 DAP are characterized by an equal number of oi2 cells compared to the wt, even though those cells have reduced length. This aspect, in addition to the reduced seed cavity area, may contribute to the reduction in total seed area observed in the double mutant. The *stk* and *abs* mutations are additive in determining the cavity area in the double mutant seeds, which is reduced compared to both the single mutants' seeds. The reduced seed cavity of *stk* single mutant

may be attributed to the reduced seed area and oi2 cell number, which was already reported (Paolo *et al.*, 2021). On the other hand, the lack of nucellus degeneration caused by *abs* mutation (Xu *et al.*, 2016) may further reduce the cavity size in the double mutant seeds. The smaller *abs stk* seeds are also characterized by a reduced aspect ratio, which indicates a rounder seed shape.

As suggested by Reviewer 2, we have increased the number of observations to n=15 seeds from 4 siliques for each genotype (Figure 1c and Source data).

Figure 1c:

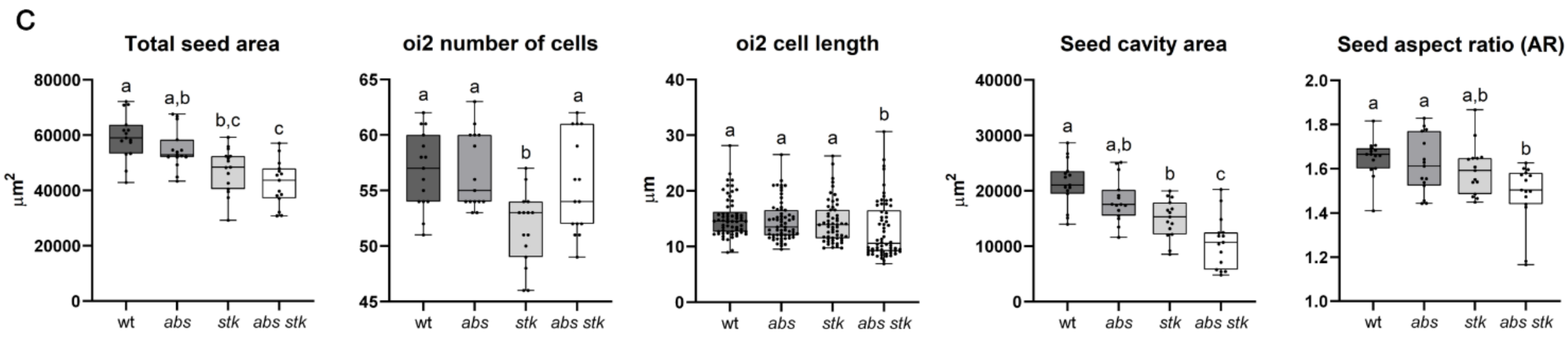

4. The expression pattern analysis of STK and ABS should extend to at least 2DAP, I think it's meaningful to check their expression pattern at the stage when nucellus difference can be clearly spotted (like the stage in Figure 1b).

We thank the reviewer for the comment. We have added new images illustrating STK-GFP and ABS-GFP expression patterns in seeds at 1, 2 and 3 DAP (Figure 1d and Supplementary Figure 1), and we have described them in the text (line 133-136). In seeds until 3 DAP, the ABS protein localization domain is restricted to the two innermost layers of the seed coat, and in the nucellus, whereas STK-GFP is localized in the three outermost layers, in the chalazal seed coat and in the funiculus.

5. The author collected pistil sample for RNA-seq analysis, this triggers my concern for the dilution effect of expression pattern analysis. As seen in Figure 2c, the fold change of gene expression is relatively not obvious, which may be related to sample collection. Although the author performed in situ hybridization to confirm the expression pattern change in double mutant and WT, but the robustness and reliability of in situ hybridization are not convincing enough. I would suggest the author use fluorescent marker lines of several candidates and perform time-lapse/sequential observation of their expression pattern during ovule development and after fertilization. This will significantly improve the quality of data and reliability of the conclusion.

We agree with the reviewer about the dilution effect that the samples used for the RNA-seq analysis (pistils) may have on identifying specific differences, which are limited to the ovule tissues. Thus, we performed a more precise visualization of differential gene expression, as suggested by Reviewer 2. Although the use of fusion proteins is an excellent suggestion, generating several marker lines and introducing them into the mutant backgrounds required a few plant generations. Furthermore, since the regulatory regions of these genes have not been previously reported, we would also need to confirm that we cloned the genomic region containing all the regulatory elements of the promoter by complementing the mutants.

Therefore, we opted for HCR-RNA FISH to visualize the simultaneous expression of selected genes in whole-mount ovules, and to identify the differences in expression pattern between *abs stk*

mutant and wt. In particular, we have tested the expression of starch-related genes *BAM4*, *TRE1* and *GPT2*, observing a consistent expression trend with respect to the RNA-seq results (Figure 2b). We have described the experiment in the Results section (line 167-177).

6. The author claimed that "ABS appears to be a key factor limiting accumulation of soluble sugars in young flowers, whereas STK tends to promote their accumulation" in the manuscript, which is not well-supported. Just based on the metabolomic analysis result and give such conclusion is not sufficient, not to mention that the author also concludes that "abs is epistatic to stk in terms of sucrose, glucose 6-phosphate and fructose 6-phosphate accumulation". To make such conclusion, solid genetic evidence is absolutely required.

We are grateful for the reviewer's input and recognize that the sentences in question were excessively overstated. Therefore, we have revised the text according to reviewer comments (line 236-237).

7. In Figure 4a, the author tried to claim the starch accumulation in ovule by Lugol staining, I would like to draw their attention to sampling stage, as it would severely interfered with the result. In principle, all ovule should be at the same stage, and the staining result should be quantified to support the conclusion.

We thank the reviewer for the comment and double-checked the ovule stages, as also mentioned in response 2 to Reviewer 2. All the pictures of mature-staged ovules (Figures 1a, 4a, and 4b, which have been renamed as Figure 1a, 5a, and 5b, respectively) were taken from pistils at stage 12c (Smyth *et al.*, 1990), ensuring that all ovules were at the same developmental stage (3-VI); this has been now specified in the Methods section. We have also checked the ovule stages by observing the mature female gametophytes. Although the resolution in Figure 4a (renamed as Figure 5a) is not high enough to recognize individual cells, in Figure 4b (renamed as Figure 5b) it is possible to count the number of integument layers and to identify the female gametophyte cells, showing that all ovules are indeed at the 3-VI stage.

Figure 5b:

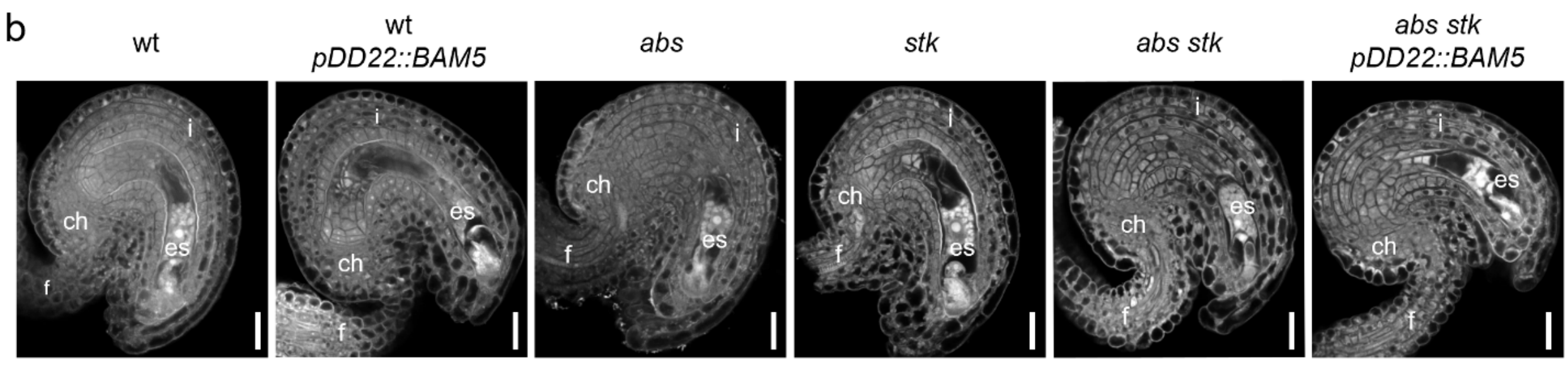

Despite that, it is possible that some ovules within the same pistil may have a slight developmental differences. Indeed, there are subtle morphological differences between the wt and the mutants (for example, the gametophyte is smaller in the *abs stk* mutant compared to the wt), which may account for some of the variations observed among the genotypes that are not due to differences in the ovule stages. To support this, we have examined several ovules for each genotype (at least 30 ovules, from 3 different plants), obtaining consistent observations. To more accurately represent a *stk* mature ovule, we have changed the previous *stk* image in Figure 4a (renamed as Figure 5a) with a new one.

Figure 5a:

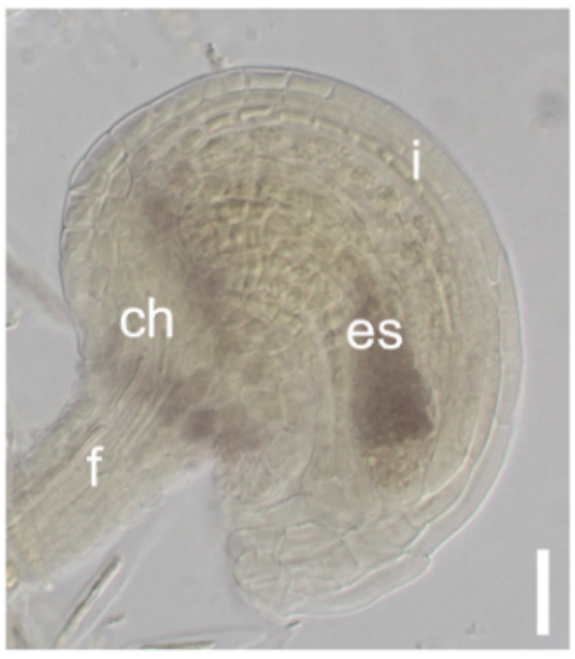

We agree with the reviewer that the quantification of the starch in the ovules would be a great support to the conclusion; however, unfortunately, Lugol staining does not allow reliable quantitative measurements. Nevertheless, the use of Lugol staining to assess macroscopic differences in starch accumulation between wild type and mutant Arabidopsis ovules was previously reported (Mizzotti *et al.*, 2012; Hedhly *et al.*, 2016). We have examined numerous *abs stk* and *abs stk pDD22::BAM5* ovules (at least 30 ovules, from 3 different plants), and we consistently observed that the Lugol staining was clearly less prominent in the latter. This difference is particularly evident in the position of the central cell (CC). However, because Lugol staining does not permit accurate quantification, we cannot estimate the magnitude of this reduction. Thus, we used "larger starch deposit" (which refers to a change in spatial distribution) instead of "higher starch" (which refers to a change in the amount of starch) in the text (line 114, line 251, line 1013, and line 1070). Likewise, we changed "the *pDD22::BAM5* construct decreases starch accumulation" to "showed less prominent Lugol signal… suggesting reduced starch accumulation" (line 254).

8. The author ectopically expressed BAM5 in central cell to observe the starch accumulation in female gametophyte. There are several concerns regarding this experimental design: first, only BAM5 can not fully represent starch metabolism, other starch-related genes/candidates should also be included; second, please consider the promoter being use here, at least explain clearly the rationale of ectopically expressed the genes in central cell.

1. We agree with the reviewer that BAM5 alone can not fully represent starch metabolism; however, its ectopic expression under the *pDD22* promoter is sufficient to partially rescue the *abs stk* phenotype. Furthermore, it has been reported that overexpression of *RsBAMY1* (*BAM5* ortholog in radish) in Arabidopsis is sufficient to reduce the starch content (Takahashi *et al.*, 2012), supporting the fact that ectopic expression of a single enzyme, such as BAM5, can indeed be enough to impact starch content.

2. We apologise for the lack of clarity. One of the major phenotypic differences between *abs stk* and wt ovules, observed in this work and previously reported in manuscripts, is the larger starch accumulation in the mutant, with the most pronounced accumulation occurring in the CC (Mizzotti et al., 2012) (Figure 1a and Figure 4a, which have been renamed as Figure 1a and Figure 5a). As the *abs stk* developing embryos show severe defects (Mizzotti *et al.*, 2012), we hypothesized that starch accumulation could be one of the causes of seed defects observed in the *abs stk* double mutant. Thus, we decided to ectopically express a starch-degrading enzyme (BAM5) inside the CC, with the unique aim of evaluating whether mobilizing the starch in the CC could rescue *abs stk* seed defects and, in particular, embryo development. Among the *BAM* genes, we chose *BAM5* because it is not

predicted to be expressed inside the CC based on single-cell data (Hou *et al.*, 2021). Furthermore, as mentioned above, it has been previously reported that the ectopic expression of its ortholog is sufficient to reduce starch accumulation (Takahashi *et al.*, 2012). We thus generated a construct in which *BAM5* expression was driven by the *pDD22* promoter, which is specifically active in the CC and endosperm nuclei (Steffen *et al.*, 2007). We have better described this in the text (line 250-255).

9. The result section regarding to callose turnover is only descriptive, based only on these observations, the conclusion at the end of this section is way too speculative. The author should either provide more direct evidence to support this conclusion (which is recommended), or at least tone down the conclusion (but this will definitely reduce the importance of this phenomenon).

We thank the reviewer for the suggestion. According to Reviewer's suggestion, we have added callose quantification to strengthen our data (see also response 4 to Reviewer 1). Second, to support the hypothesis that *abs stk* fertility defects are mainly due to the lack of callose degradation at the chalazal region of the seed upon fertilization, we have generated *pSTK::BG_PPAP* and *pSTK::MEE48* constructs and introduced them into the *abs stk* mutant background. This strategy aimed to ectopically express callose-degrading enzymes (β-1,3-Glucanases) in the chalazal seed coat of the double mutant, where the *STK* promoter is active, to rescue at least partially the *abs stk* fertility phenotype (Figure 9). We have chosen to express *BG_PPAP* and *MEE48* genes because it has been reported that *BG_PPAP* constitutive expression is able to increase the nutrient transport in the Arabidopsis seed (Liu *et al.*, 2025), while *MEE48* ortholog was used to reduce callose deposition in Arabidopsis ovules (Pinto *et al.*, 2024). We described these experiments in the last paragraph of the Results section. See also response 11 to Reviewer 2.

10. I have general concern for the sample selection of RNA-seq in the manuscript. Using silique is simple, but may not be perfect to reflect the precise change that the authors are interested. This is not easy to modify, that I am truly aware, so the author needs to perform extra experiments to confirm it, like the what I commented on Figure 2c, also applies to Figure 7. Of course, not the same experiments are required, but further confirmation is definitely needed.

We thank and agree with the Reviewer's comment that the expression changes derived from RNA-seq on siliques may not accurately reflect the expression changes that specifically occur in seeds. Therefore, we opted for HCR-RNA FISH to visualize the simultaneous expression of selected genes in whole-mount seeds, and to identify the differences in expression pattern between *abs stk* mutant and wt. In particular, we have tested the expression of callose-related genes *BG1* and *PDLP1*, and auxin-related gene *ARF5/MP*, observing a consistent expression trend with respect to the RNA-seq results (Figure 8b). We have described this experiment in the Results section (line 324-327 and 338-342).

11. The model is quite clear, but to support the blocking effect, please provide extra experimental evidence (genetic investigation would be the best), for example, if the author could artificially block the callose degradation at the phloem end gate and the similar effects can be observed, that will be fantastic.

We thank the reviewer for the suggestion. It has already been described that mutation of *BG_PPAP*, encoding a callose degrading enzyme, causes reduced nutrient transport in seeds, which is associated with reduced seed size (Liu *et al.*, 2025). However, the sole mutation of this gene was

not sufficient to completely block the transport. In fact, the CFDA tracer was still present in the seed coat of *bg_ppap* mutant seeds, even if in lower amount.

As also stated in response 9 to Reviewer 2, to prove that *abs stk* fertility defects are due to the lack of callose degradation at the chalazal seed coat, we have generated *pSTK::BG_PPAP* and *pSTK:MEE48* constructs and introduced them into the *abs stk* mutant background. This strategy aimed to ectopically express callose-degrading enzymes (β-1,3-Glucanases) in the chalazal seed coat of the double mutant, where the *STK* promoter is active, to rescue at least partially the *abs stk* fertility phenotype (Figure 9). As stated above, *BG_PPAP* constitutive expression is able to increase the nutrient transport in the Arabidopsis seed (Liu *et al.*, 2025), while *MEE48* ortholog was used to reduce callose deposition in Arabidopsis ovules (Pinto *et al.*, 2024). We have shown and reported in the revised version of the manuscript (last paragraph of the Results section) that in one *pSTK::BG_PPAP abs stk* line, and in two independent *abs stk pSTK::MEE48* transformant lines, the ectopic expression of β-1,3-Glucanase partially rescues the seed set compared to the *abs stk* control plants, as we observed a higher percentage of developing seeds inside the siliques of the transformant lines (Figure 9a). We have quantified callose accumulation in the chalazal region of ovules, measured as CTCF (Corrected Total Cell Fluorescence) of aniline blue signal (see also response 4 to Reviewer 1), of those lines confirming that the constructs expression causes a reduction of callose amount compared to *abs stk* mutant (Supplementary Figure 13). Nevertheless, the callose quantity was still higher than the wild type, explaining the only partial rescue of the phenotype. We have also analysed CFDA tracer in 3 DAP seeds of *pSTK::BG_PPAP abs stk* and *pSTK::MEE48 abs stk* lines. We have observed a rescue in tracer distribution in only few seeds (5-10%) compared to *abs stk* lines in which the tracer is blocked in the chalazal seed coat in 100% of seeds (we have analysed at least 90 seeds from 3 plants for each genotype) (Figure 9b). We described these experiments in the last paragraph of the Results section.

Figure 9:

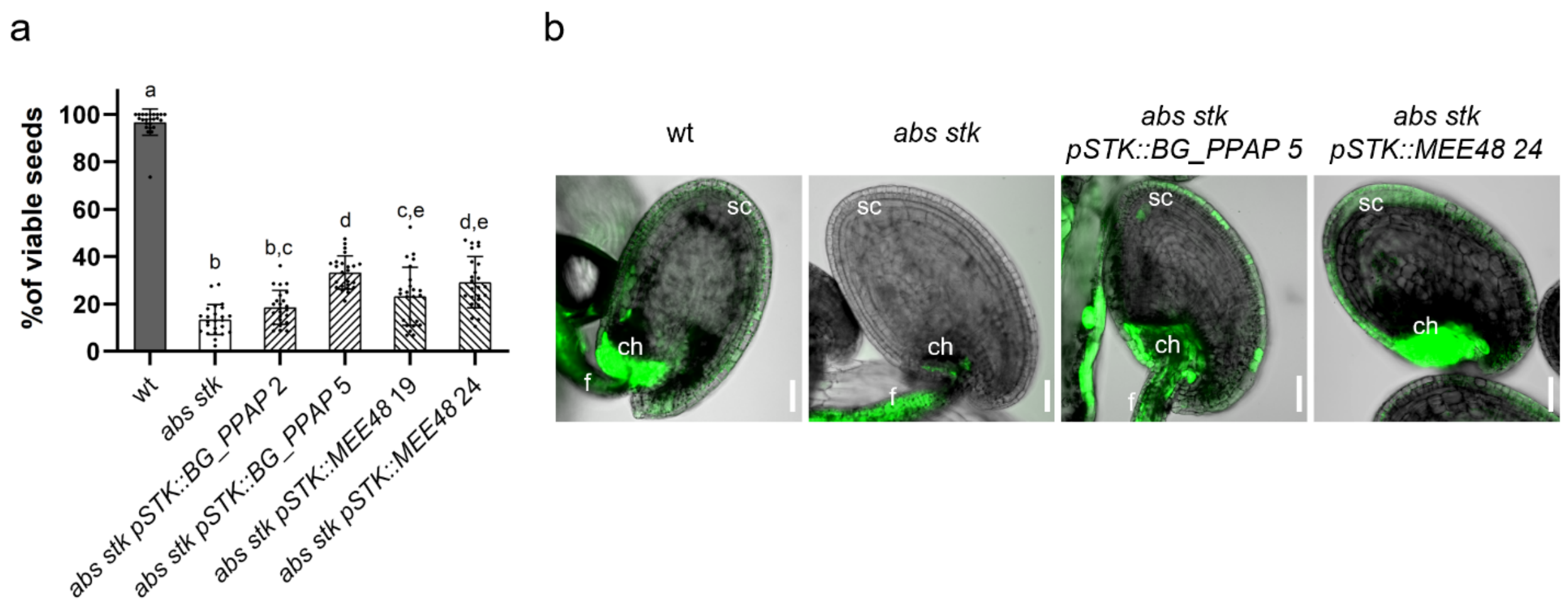

Minor issues:

1. Staining result should be quantified, also in situ hybridization and other staining.

We have quantified the fluorescence signal of aniline blue staining (Figure 7 and Supplementary Figure 13) as described in response 4 to Reviewer 1 and in the text.

2. Supplementary Figure 6c, please provide figures with better quality, in this version, it's difficult to see the difference.

We apologise for the low-quality of the images. We have added better pictures to Supplementary Figure 6c that has been renamed to Supplementary Figure 7c.

3. The author could also consider Whole amount in situ hybridization instead of sectioned samples, which might be more easy to see the difference and more reliable.

We thank the reviewer for the suggestion. We have performed whole-mount HCR-RNA FISH on ovules and seeds (Figure 2b and 8b), obtaining comparable results to digoxigenin-based *in situ* hybridization that were already included in the first version of the manuscript (Supplementary Figure 3).

Reviewer #3 (Remarks to the Author):

The manuscript describes and interesting, well-planned and well-executed study. The authors have combined their experience and skills in analysis of ovule structure and ovule development mutations with recent results on the importance of nutrient transport into fertilized ovules for seed filling/growth (e. g. Liu, X. et al., 2025) to extend these studies.
They first show that there is a synergistic effect of a combination of the abs and stk mutations on ovule morphology and anatomy, detailing these effects in a series of excellent micrographs in Fig. 1. They further confirm prior results that the double mutant accumulates a higher level of starch than wild type, and this was previously shown to persist following fertilization, in contrast to wild type where the starch decreases dramatically.
Using functional genes producing GFP fusion proteins they show that ABS and STK have complementary non-overlapping expression patterns in ovules. This partly contradicts prior work from this group (Misszzotti et al, 2012) where in situ hybridization showed similar complementary expression patterns, except that there was overlapping signal in the ovule endothelium. Endothelium expression is not visible for either gene here. This could be a difference in protein accumulation vs. mRNA accumulation, or a result of the transgene analysis. Since the transgenes are functional, if there is mRNA production in the endothelium it is not functionally relevant.
Transcriptomics were used to test for changes in gene expression in the mutant lines. No additive effect on DEGs were observed for the two mutations combined. But this may not be meaningful since the RNA was isolated from complete gynoecia and the genes appear to affect only specific tissue of the ovules so effects on specific regions would not be observed. Still, these studies provide information on candidate genes for regulation that could affect starch metabolism. Figure 2 and Supplementary Figure 1 display analysis of these data and are clear and informative. The authors also cite Supplementary Tables 1 and 2, but these are not tables, but multi-tab Excel files of data and analysis of data. They provide support for the figures, but are not nearly as simple to interpret as would be tables summarizing the data.

We apologise to the reviewer for the lack of clarity. We have changed the names of the multi-tab Excel files, previously referred to as Supplementary Tables, into Supplementary Datasets. Following the reviewer's suggestion, we have also included a description of the contents of each Supplementary Dataset in the first sheet of each file. In addition, to provide a clearer and more

accessible summary of the RNA-seq and ChIP-seq data, we have generated Supplementary Table 1 and 2 (previously presented as multi-tab Excel files).

They tested expression of two genes, TRE1 and BAM4 by examining in situ hybridization results. These results appeared to confirm the lower expression of both genes. This is important because this shows that the downregulation is manifest in ovules, rather than in other pistil tissues included in the RNA-seq analysis.
Metabolomics was applied to detect observable effects on starch metabolism that could be associated with the persistence of starch in the double mutant. Figure 3a and supplementary figures 3 and 4 provide clear informative displays of the most relevant results of this analysis. Supplementary Table 3 is an Excel datafile of the results that backs up these figures, but is again, not a table. The results show clear changes in levels of important intermediates in starch metabolism between the double mutant and both wild-type and single mutant lines. In figure 3b they combine the results of the transcriptomic, metabolomic, and starch staining results to illustrate how the changes in gene expression correlate with the changes in metabolite level in a pattern that could explain the starch accumulation/persistence in the double mutant. The illustration of the results on the different DEGs with the changes in carbohydrates is compelling. This is a clear, informative figure. The only change I would suggest is that the change in starch level is not reflected in the diagram. While it was not the metabolomic results that indicated the elevated starch, I would still add a red up arrow to the starch oval to indicate that it is also elevated as this would make it more clear how the other changes relate to starch. (Unless I have missed something as to why this should not be done).
ChIP-seq was used to see if the subject DEGs might be directly regulated by STK. About one is six genes in the Arabidopsis genome were found to be bound by STK. These include genes in carbohydrate metabolism and transport. Examination of the information in the dataset (referred to as Supplementary table 4) I found that many of the genes that were focused on in the earlier sections were included in the regulated list (notable genes such as BAM4 and TRE1). Some mention of these genes that were focused on earlier in the text would help the manuscript as is significant work for a reader. The results confirm that it is possible that genes important in starch accumulation and degradation could be direct targets of STK regulation.

We thank the reviewer for the suggestion. We have now included in the Results section a description of the STK target genes involved in starch and sucrose metabolism (line 208-212). Additionally, we have included images for ChIP-seq peak visualization in both pre- and post-fertilization stages (Figure 3b and Figure 8d). These images specifically represent the genes associated with starch and sucrose metabolism (pre-fertilization), and callose turnover, sugar transport and auxin response (post-fertilization) that are differentially expressed between *abs stk* mutant and wt in pre- and post-fertilization RNA-seq data, respectively.

In response to the reviewers' suggestions (see also Response 3 to Reviewer 1), we have also added Venn diagrams comparing the RNA-seq (*abs stk* vs wt comparison) and ChIP-seq data in both pre- and post-fertilization stages (Figure 3a and Figure 8c, respectively) and summarized these data in Supplementary Table 1 and 2. We described this in the Result section, line 208-212 and 362-372, respectively. We thank the reviewer for the comments since we believe that these additions will contribute to directly find information about the direct STK target mentioned in the text without sorting through the large dataset in Supplementary Datasets.

Use of a central cell-expressing transgene producing an isozyme of a starch degrading enzyme was an excellent test of the hypothesis that the persistent starch of the double mutant played a role in the mutant phenotypic effects. The transgene was shown not only to reduce the CC starch, but to at least partially reverse all of the phenotypic effects of the double mutant including cell divisions, embryo development and viable seed formation. Thus, the aberrant persistence of the starch and failure to release its products is an important part of the effect of the double mutant.
Callose accumulation in the chalaza has previously been shown to constitute a reversible block to seed development that is normally reversed by fertilization. A transported fluorescent tracer was used to confirm the callose block that is reversed by fertilization in wild-type and the two single mutants. However, this block persisted following fertilization in the double mutant. The association of these changes in permeability with callose quantity was confirmed by direct staining for callose which showed that callose removal was decreased in the chalaza of the double mutant. This continued interruption of nutrient flow in the double mutant could explain why the transgene promoting starch mobilization was not sufficient to fully restore seed formation in the double mutant.
The authors applied RNA-seq and ChIP-seq to examine regulation of callose degradation and sugar transport. They found results consistent with ABS and STK promoting expression of genes involved in callose metabolism. A case for modulation of sugar transport was less strong. The model they propose for regulation of nutrient flow into the fertilized ovule is supported by their data.
Overall the authors have used an impressive array of approaches to identify and characterize the likely pathway for the effects on development and fertility in the abs stk double mutant. Rather than being a direct regulation of anatomical features (e. g. the additional cell layer in the integument or development of the endothelium), they are shown to be derivative effects of nutrient restriction in the fertilized ovule. This restriction is the result of a combination of failure in mobilization of nutrients from stored starch, and a failure to remove the chalazal callose that restricts nutrient flow from the funiculus. Hence the fertilized ovules behave more like unfertilized ovules and fail to develop. Their work extends other recent work on nutrient transport into ovules with important regulatory information. The manuscript is clear, well-organized and well-written.
The methods section appears sufficient to enable reproduction of the presented work.
I have only a small number of specific comments for improvement of the manuscript.
1) line 177, I would say “mis-regulation” instead of “deregulation” because both lower and higher expression are observed.

We thank the reviewer for the suggestion, and we modified the text accordingly (line 247).

2) The text refers multiple times to Supplementary Tables. The initial version of the manuscript file page did not have any supplementary tables listed. When I asked the publisher for correction, the names of the supplementary data files were changed to Supplementary Table X etc. But these files don’t contain tables, but rather are multi-tab excel data files. These files should be referred to as “Supplementary Datafile X”. If they want supplemental tables, then they should prepare them (some would actually be useful if they prepared summaries from the supplemental data files of the most pertinent genes).

We apologise to the reviewer for the lack of clarity. We have modified the multi-tab excel data files’ name (previously named Supplementary Tables) to Supplementary Datasets. To summarize the RNA-seq and ChIP-seq data in a clearer way, we generated Supplementary Table 1 and 2 as the Reviewer suggested.

In addition, the text refers to “Supplementary Table 8” which appears in the list of files on the manuscript page at the publisher, but does not appear on the list of Supplementary Tables at the end of the manuscript.

We apologise for the mistake. We have corrected the list of Supplementary Files and Supplementary Datasets at the end of the manuscript according to the revisions.

Also in the set of files on the manuscript page at the publisher there is a “Supplementary Table 9” that also does not appear in the list of supplementary information nor anywhere else in the manuscript. This all should be cleared up prior to publication.

We apologise to the reviewer for the lack of clarity. The “Supplementary Table 9” in the set of files on the manuscript page contains the Source Data file. We modified the file name for clarity.

3) The results show that it appears to be the lack of carbohydrates released from starch degradation, rather than the presence of starch in the ovule that prevents development. So perhaps refer more to starch mobilization that to starch degradation in the text to emphasize this point. E. g. line 386 they refer to “reducing the starch in the embryo sac” when in fact it is probably the release of degradation products that is the more important aspect of this process.

We thank the reviewer for the suggestion and we agree that the key aspect is the mobilization of carbohydrates derived from starch, rather than the starch degradation itself. We have thus modified “starch degradation” to “starch mobilization” throughout the text accordingly.

References:

Hedhly, A., Vogler, H., Schmid, M.W., Pazmino, D., Gagliardini, V., Santelia, D. and Grossniklaus, U. (2016) Starch turnover and metabolism during flower and early embryo development. *Plant Physiol.*, **172**, 2388–2402.

Hou, Z., Liu, Y., Zhang, M., Zhao, L., Jin, X., Liu, L., Su, Z., Cai, H. and Qin, Y. (2021) High-throughput single-cell transcriptomics reveals the female germline differentiation trajectory in Arabidopsis thaliana. *Commun. Biol.*, **4**. Available at: http://dx.doi.org/10.1038/s42003-021-02676-z.

Liu, X., Nakajima, K.P., Adhikari, P.B., et al. (2025) Fertilization-dependent phloem end gate regulates seed size. *Curr. Biol.*, **35**, 2049-2063.e3. Available at: https://doi.org/10.1016/j.cub.2025.03.033.

Mizzotti, C., Mendes, M.A., Caporali, E., Schnittger, A., Kater, M.M., Battaglia, R. and Colombo, L. (2012) The MADS box genes SEEDSTICK and ARABIDOPSIS Bsister play a maternal role in fertilization and seed development. *Plant J.*, **70**, 409–420. Available at: https://onlinelibrary.wiley.com/doi/10.1111/j.1365-313X.2011.04878.x.

Paolo, D., Rotasperti, L., Schnittger, A., Masiero, S., Colombo, L. and Mizzotti, C. (2021) The Arabidopsis MADS-Domain Transcription Factor SEEDSTICK Controls Seed Size via Direct Activation of E2Fa. *Plants*, **10**, 192. Available at: https://www.mdpi.com/2223-7747/10/2/192.

Pinto, S.C., Leong, W.H., Tan, H., et al. (2024) Germline β−1,3-glucan deposits are required for female gametogenesis in Arabidopsis thaliana. *Nat. Commun.*, **15**. Available at: http://dx.doi.org/10.1038/s41467-024-50143-0.

Smyth, D.R., Bowman, J.L. and Meyerowitz, E.M. (1990) Early flower development in Arabidopsis. *Plant Cell*, **2**, 755–767.

Steffen, J.G., Kang, I.-H., Macfarlane, J. and Drews, G.N. (2007) Identification of genes expressed in the Arabidopsis female gametophyte. *Plant J.*, **51**, 281–292. Available at: http://www.arabidopsis.org.

Takahashi, I., Kuboi, T., Fujiwara, T. and Hara, M. (2012) Overexpression of an extraplastidic β-amylase which accumulates in the radish taproot influences the starch content of Arabidopsis thaliana. *Plant Biotechnol.*, **29**, 447–455.

Xu, W., Fiume, E., Coen, O., Pechoux, C., Lepiniec, L. and Magnani, E. (2016) Endosperm and nucellus develop antagonistically in arabidopsis seeds. *Plant Cell*, **28**, 1343–1360.

REVIEWERS' COMMENTS

Reviewer #1 (Remarks to the Author):

The authors did a great job in integrating my (and the ones of the other reviewers) comments into this revised version of the manuscript. This is a great piece of work.

We thank the reviewer for the positive comment and for appreciating the revised version of our manuscript.

I only have three textual remarks:
line 76: What is STP?

We thank the reviewer for the comment. STP (Sugar Transporter Protein) is a clade of transporters belonging to the MST superfamily. We have now specified that in the text (line 76).

Line 223: "122 out of 135 metabolites significantly differed between the wt and at least one of the three mutants ". I think you meant that their amount significantly differed?

We thank the reviewer for pointing that out; we modified the text according to the reviewer's suggestion (line 223).

Line 268: "the development of abs stk embryos was delayed". To reflect on this observation, the seed set in the double mutant is reduced. One would have expected that the opposite trend (faster development) as (more) resources are available for less seeds. Yet the development is delayed and seeds are smaller. So, is this delay really because of carbohydrate access? Also, less seeds per silique may bias the measurement.

We thank the reviewer for the comment, and we apologise for the lack of clarity. Our data show that *abs stk* double mutant is characterized by increased callose accumulation in the chalaza of ovules and early seeds, which may restrict the nutrients from being released at the phloem end gate. This interpretation is supported by CFDA tracer analysis, which showed that the tracer movement was blocked at the seed chalaza of *abs stk* seeds. Thus, the increased callose accumulation observed in the double mutant is consistent with reduced nutrient flow in developing seeds. Interestingly, this phenotype was already associated with reduced seed dimensions in *bg_ppap* mutant (Liu et al., 2025). Moreover, we observed a delay in the early stages of embryo development in *abs stk* mutant compared to wt, *abs* and *stk* single mutants. This delay was rescued by mobilising sugars by introducing *pDD22:BAM5* construct into the *abs stk* mutant background. Together, these results support a contribution of reduced nutrient availability to the delayed embryo development in *abs stk* seeds. We agree with the reviewer that the reduced seed set in *abs stk* mutant could potentially influence seed development. However, the observation of impaired nutrient flow at the chalaza, together with the rescue of the embryo delay by promoting sugar mobilisation, supports that carbohydrate availability is likely linked to this phenotype.

Reviewer #2 (Remarks to the Author):

After evaluating the revised manuscript and the authors response, I am basically satisfied that all my previous concerns have been addressed properly. The authors have done additional experiments and

analyses, which strengthened the manuscript. Although the genetic analysis is still missing, I hope the authors can make careful interpretation and be aware of this weakness.

We thank the reviewer for appreciating our revisions and additional experiments. We are aware that our work has some limitations, and we agree that future studies incorporating genetic analyses will be important to strengthen our findings.

Reviewer #3 (Remarks to the Author):

The authors have fully addressed my concerns on the original manuscript through their revisions. The addition of tables I and II to the supplementary information are especially appreciated. My reading of the responses to the other reviewers indicates further improvement of the manuscript.
I have no additional suggestions.

We thank the reviewer for recognising the improvements we made to our work.
