# Supplementary material for: ARABIDOPSIS Bsister and SEEDSTICK MADS-box transcription factors modulate maternal nutrient flow for seed development in Arabidopsis
Source: Nat Commun. 2026 Sep 10;17:10687. doi: 10.1038/s41467-026-77573-2 (PMC13655123; doi:10.1038/s41467-026-77573-2)
Supplement: Supplementary file 3 [file 41467_2026_77573_MOESM1_ESM.pdf]

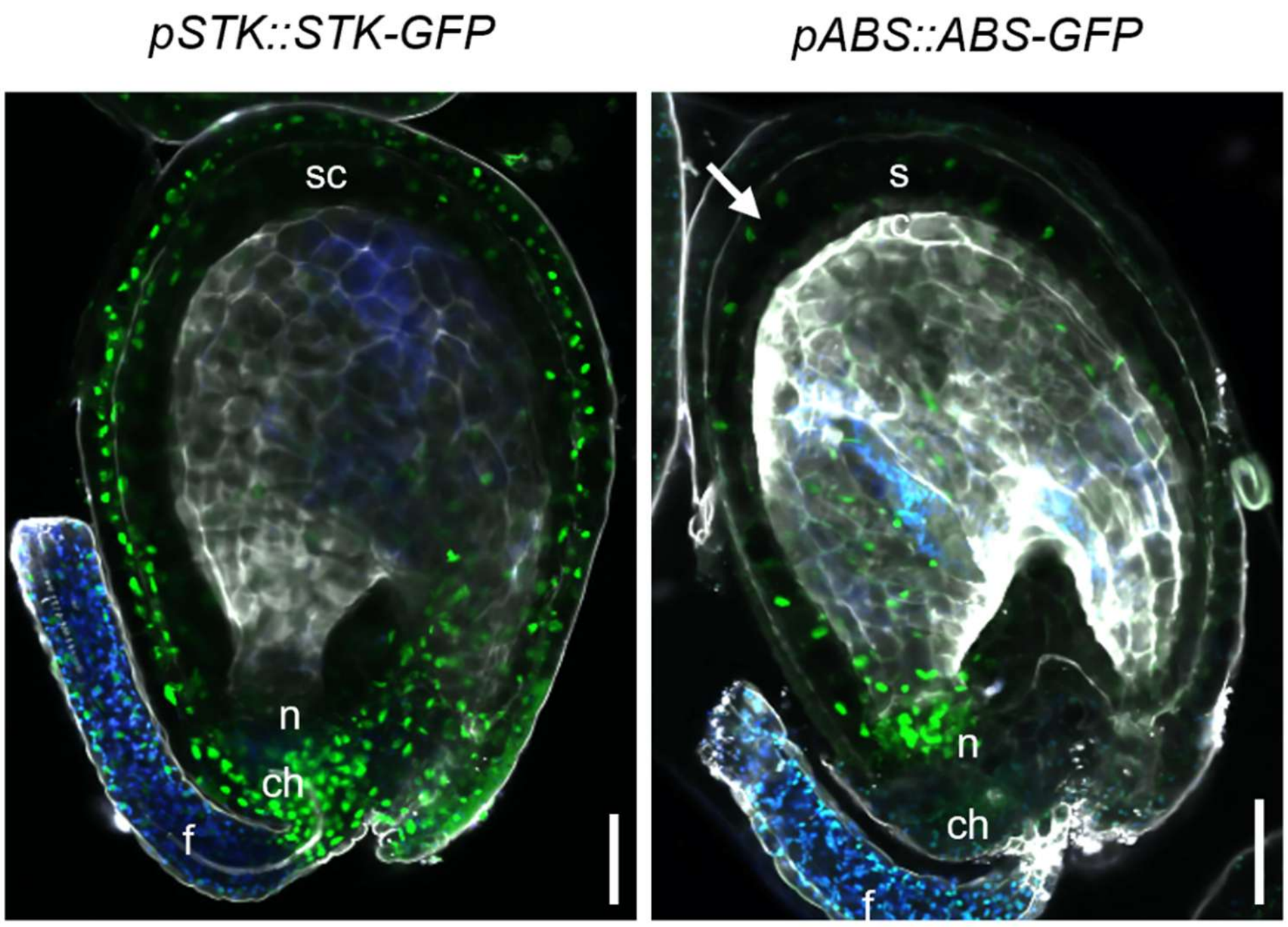

Supplementary Fig. 1. STK and ABS protein localization in seeds at 3 DAP. Confocal images showing the expression of *pSTK::STK-GFP* and *pABS::ABS-GFP* in seeds. GFP signal is marked in green, cell walls are marked in white, and chlorophyll autofluorescence is marked in blue. The white arrow indicates ABS-GFP signal in the two inner layers of the seedcoat. ch: chalaza, f: funiculus, n: nucellus, sc: seed coat. Scale bar 50 μm.

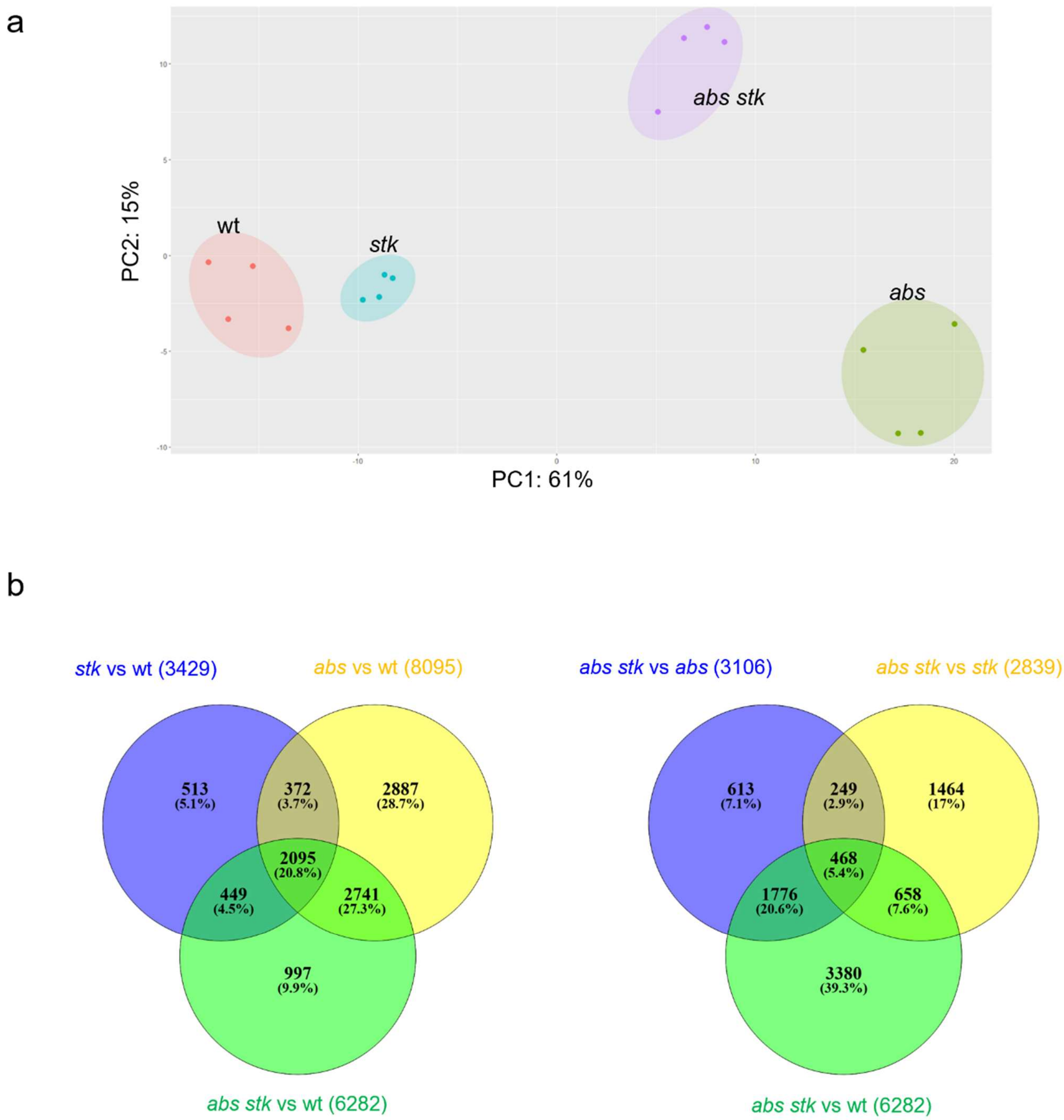

Supplementary Fig. 2. PCA and Venn Diagrams of RNA-seq data pre-fertilization. a Principal Component Analysis (PCA) displaying the first two principal components (PC1, PC2) shows good clustering of the replicates of the four different RNA-seq samples (wt, *abs*, *stk*, *abs stk*). **b** Venn Diagrams of the DEGs in mutant to wt comparisons and in the double to single mutants comparison. FDR ≤ 0.05 was considered to identify the DEGs. n=4.

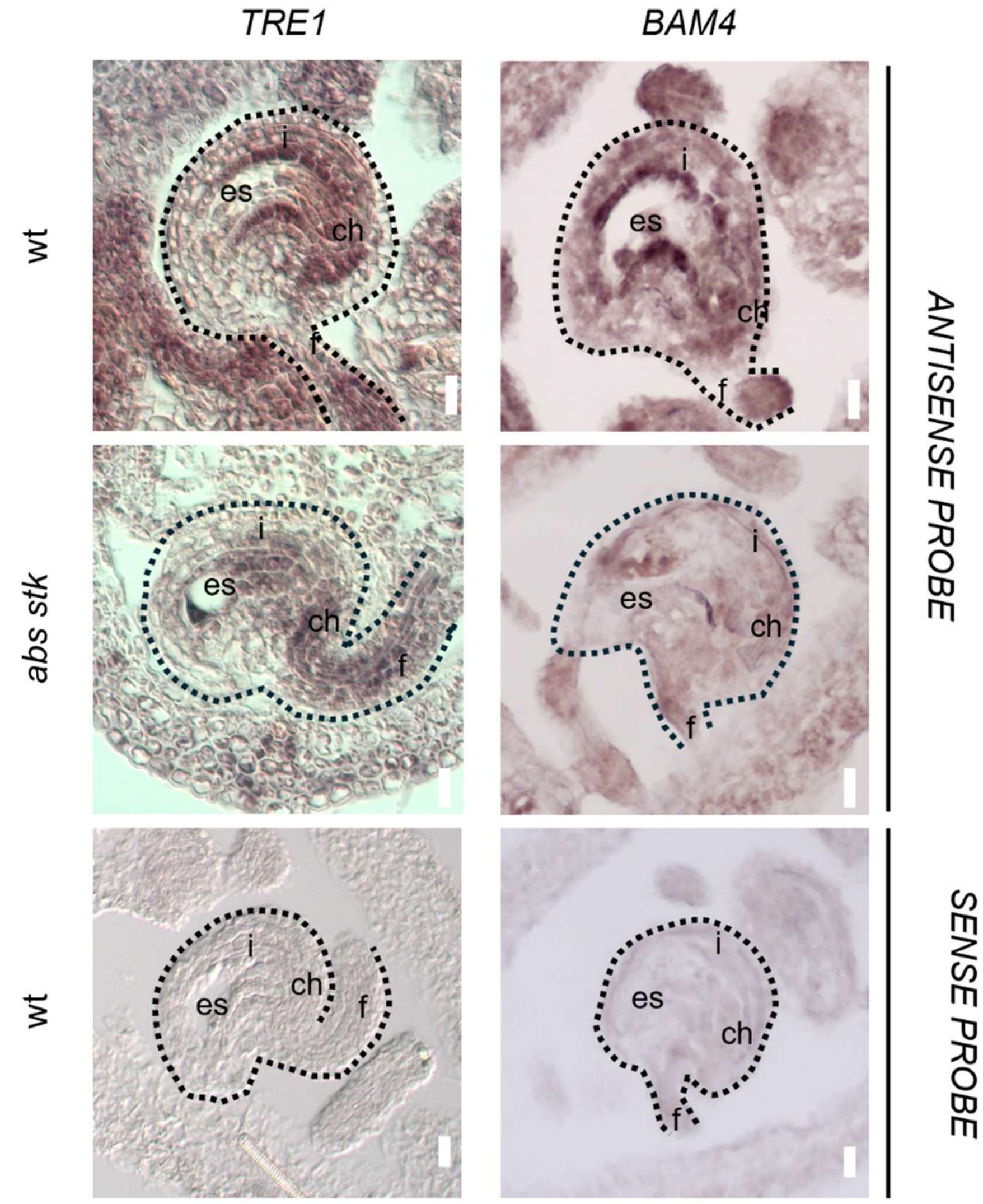

Supplementary Fig. 3. ***In situ*** **hybridization on** ***TRE1*** **and** ***BAM4*** **in wt and** ***abs stk*** **ovules.** The ovule border is highlighted by dotted line. es: embryo sac, f: funiculus, i: integuments, ch: chalaza. Scale bar 20 µm. Experiments were repeated on three independent individuals with similar results, and representative images are shown.

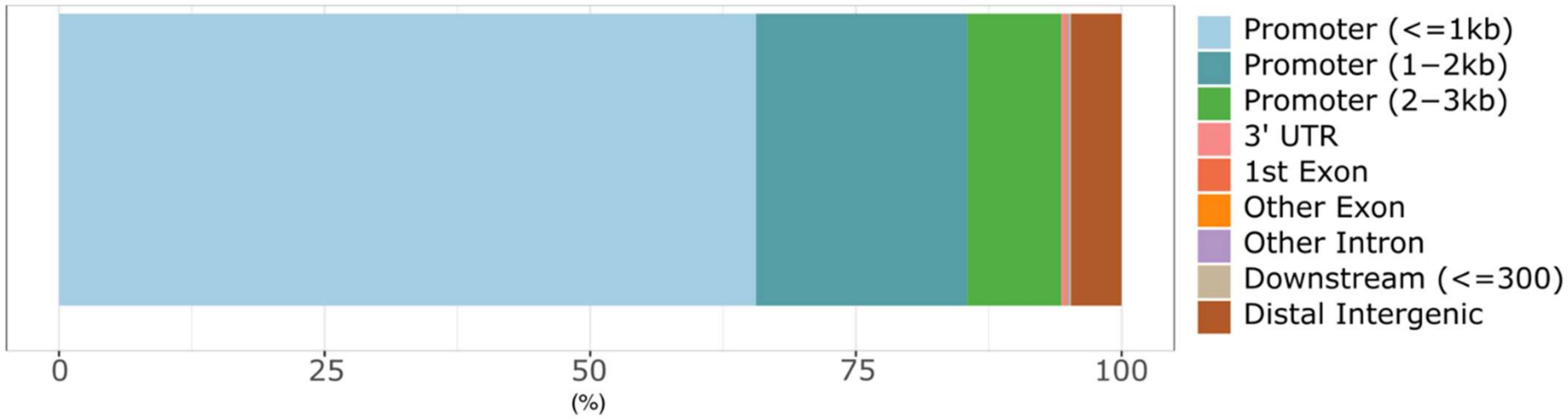

Supplementary Fig. 4. AnnoBar plot of STK ChIP-Seq data pre-fertilization. AnnoBar plot represents the percentage of peaks in the genomic region of STK target genes.

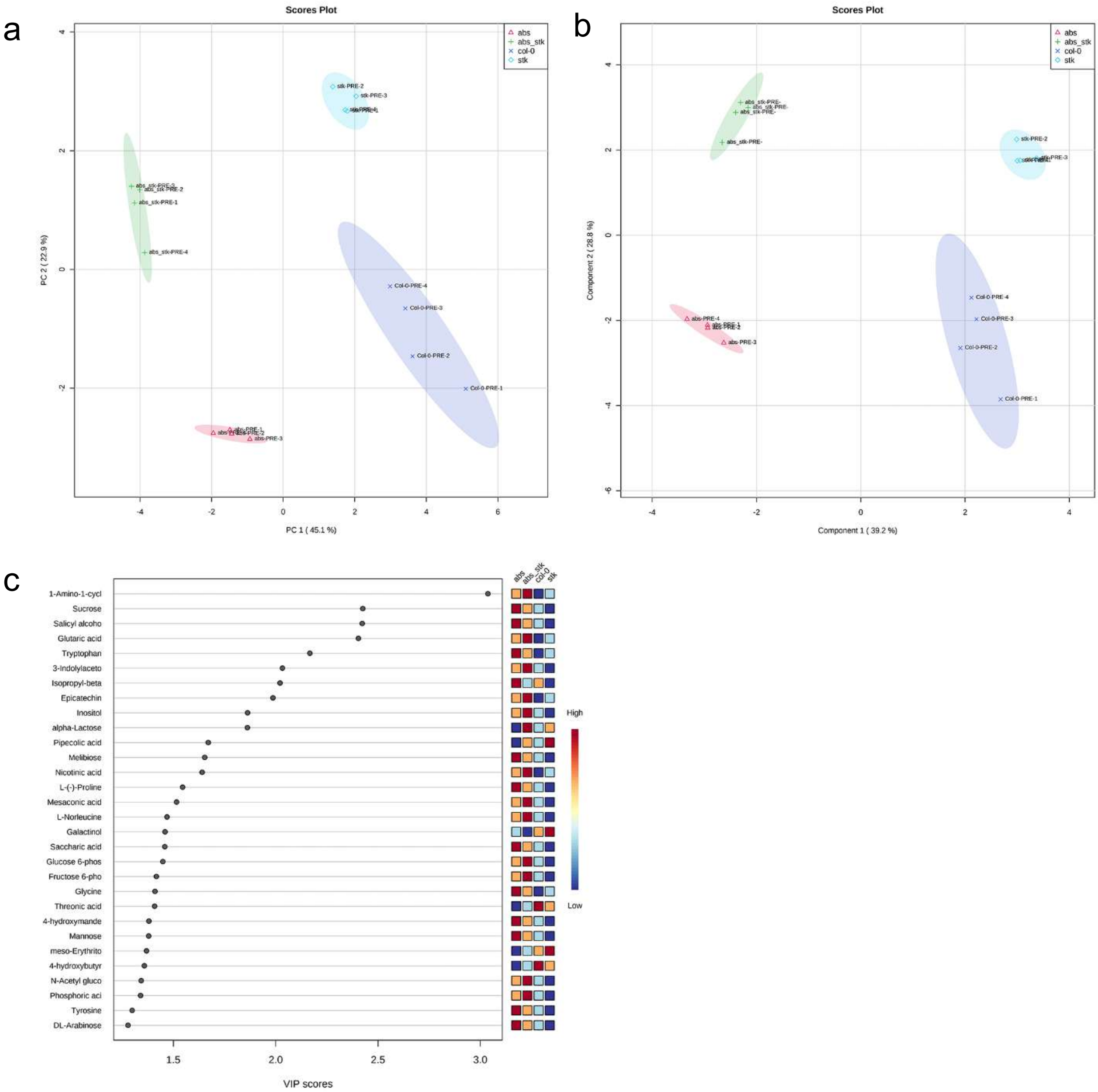

Supplementary Fig. 5. Different statistical analyses performed on the metabolome profile conducted on the wt and mutant flowers pre-fertilization. a PCA score plot, **b** PLSD-A score plot and **c** related VIP scores of wt (Col-0) and mutants (*stk, abs*, and *abs stk*) in Col-0 background. n=4.

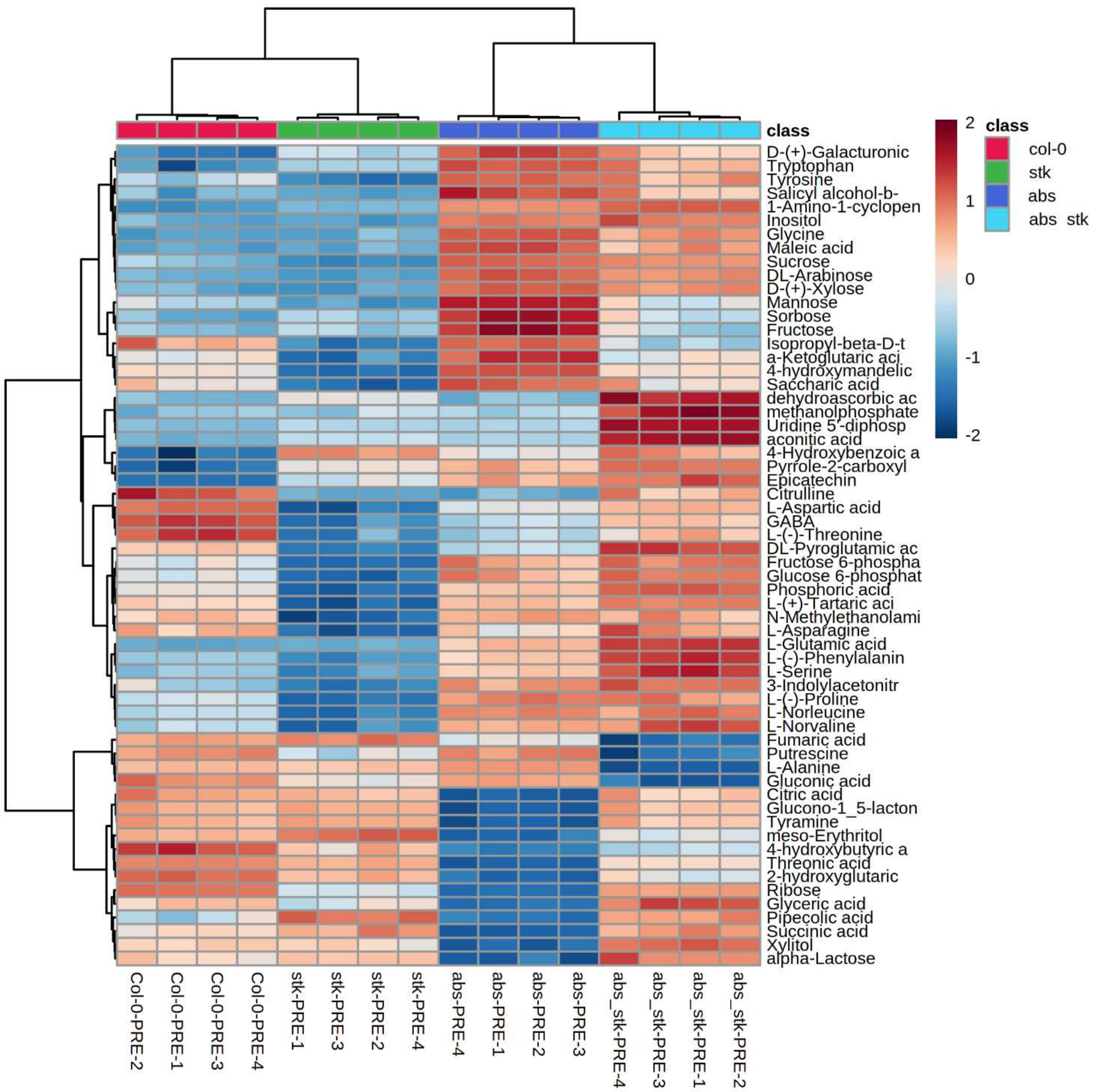

Supplementary Fig. 6. Heatmap showing differentially accumulated metabolites in wt and mutant plants. Clustering result shown as a heatmap built with the top 60 out of 122 metabolites with significant differences as determined by univariate one-way ANOVA using LSD test as post-hoc. Each rectangle represents the amount of a metabolite in false-color scale. Dark red or dark blue regions indicate an increase or decrease in metabolite content, respectively. Col-0: wt.

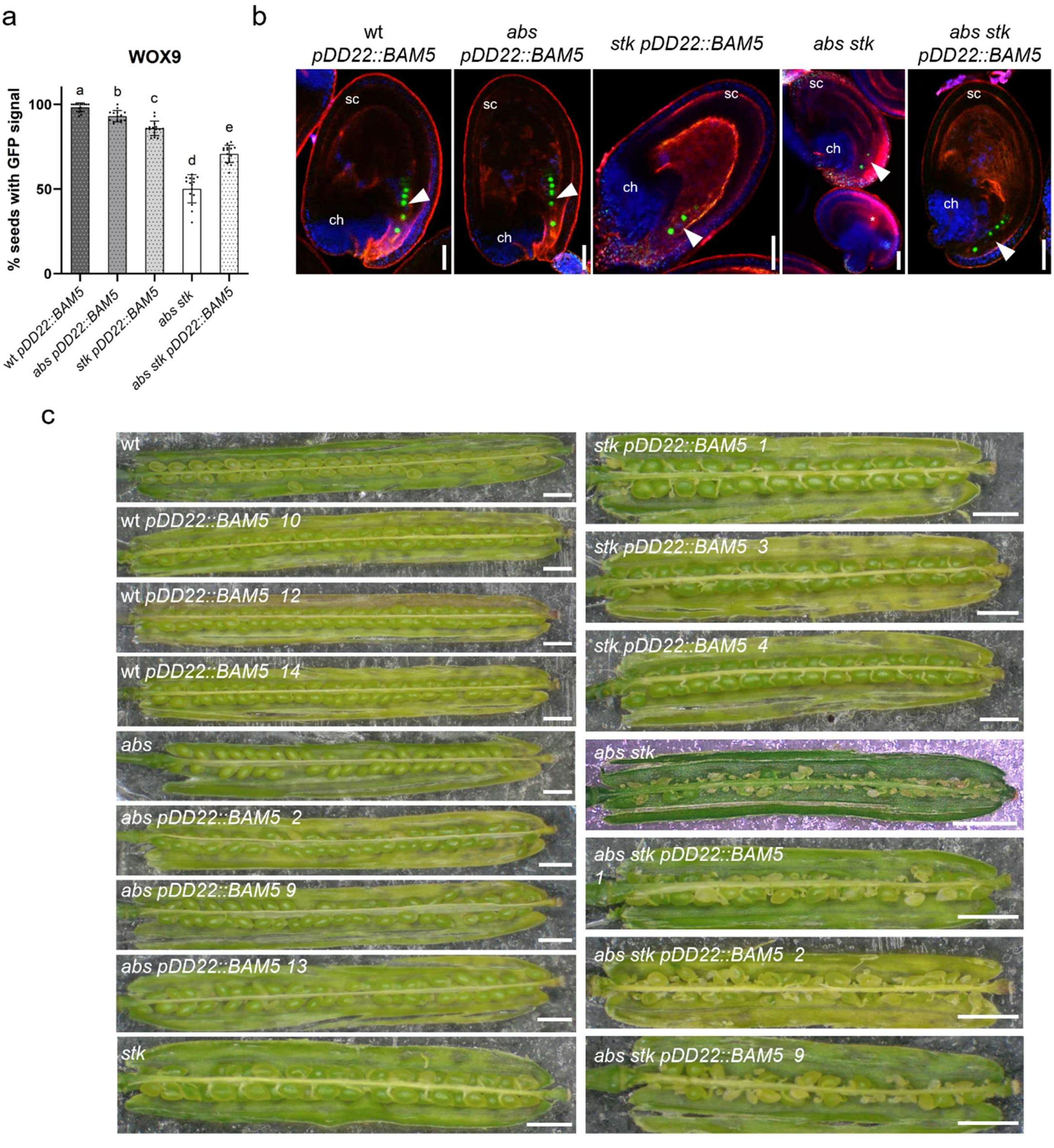

Supplementary Fig. 7. Characterization of the *pDD22::BAM5* transformant lines. a Percentage of seeds showing a WOX9-GFP signal at 3 DAP in wt *pDD22::BAM5*, *abs pDD22::BAM5*, *stk pDD22::BAM5*, *abs stk*, *abs stk pDD22::BAM5* (n=15). The dots represent the single values, whereas the bars represent the standard deviation. Different letters above the bars represent significant differences ($p < 0.05$) as determined by one-way ANOVA followed by Tukey's HSD, whereas the same letters indicate absence of statistical difference between the respective genotypes. Three independent measurements showed similar results. Source data are provided as a Source Data file. **b** Confocal images of seeds of *abs stk* and *pDD22::BAM5* transgenic lines expressing the WOX9-GFP protein in the suspensor of the embryos (arrowheads). **c** Siliques of the different transgenic lines and controls analyzed. ch: chalaza, sc: seed coat. Scale bars **b**: 50 µm **c**: 1mm. Experiments were repeated on at least three independent individuals with similar results, and representative images are shown in **b**, **c**.

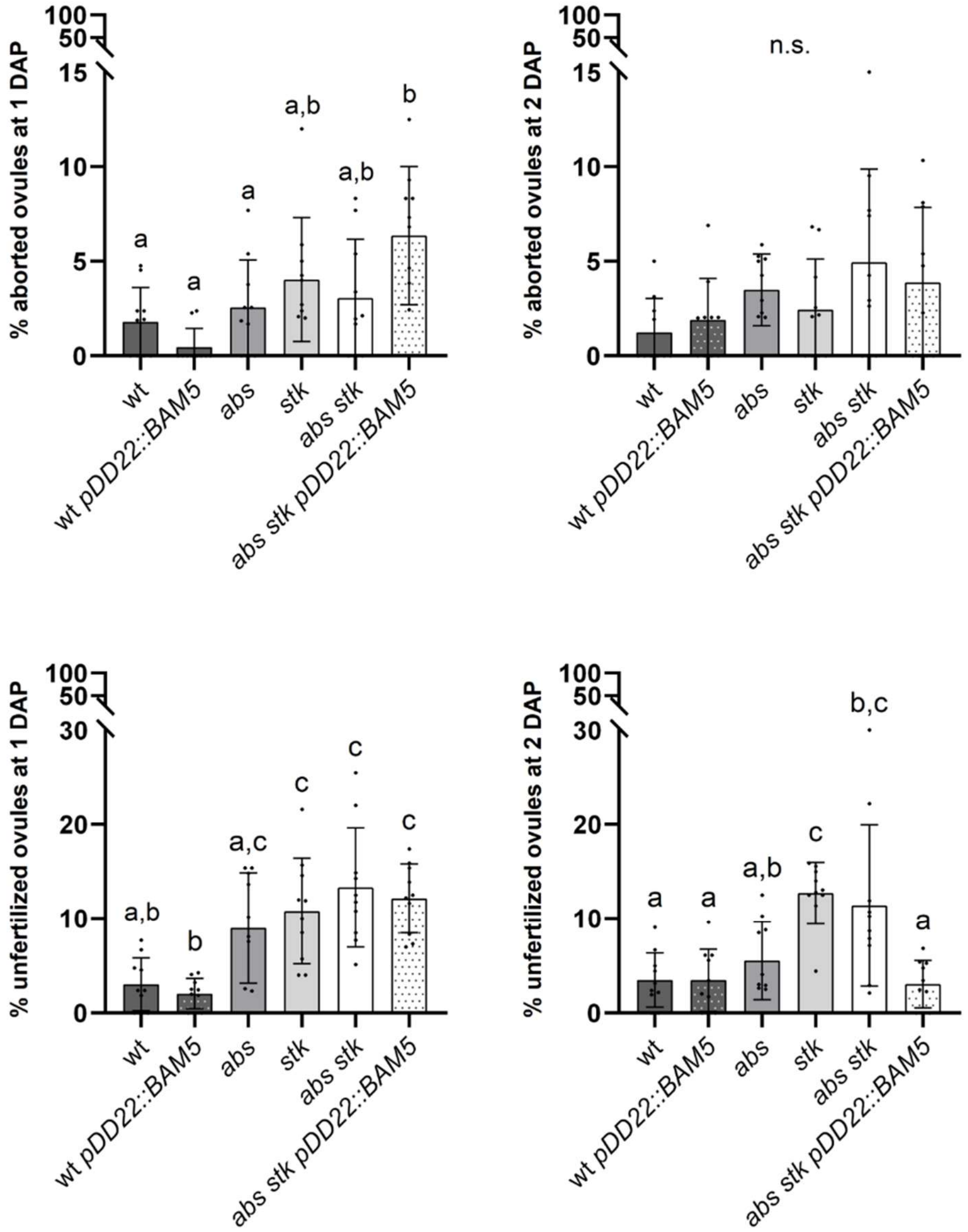

Supplementary Fig. 8. Percentage of aborted and unfertilized ovules in *pDD22::BAM5* transformant lines assessed by confocal imaging at 1 and 2 DAP. Bar plots showing the percentage of aborted and unfertilized ovules in the same analysis reported in Fig. 6 (n=10 siliques). The graphs represent the mean values ± SD, and the dots represent the single values. Different letters above the bars represent significant differences ($p < 0.05$) as determined by one-way ANOVA followed by Tukey's HSD, whereas the same letters indicate absence of statistical difference between the respective genotypes. Three independent measurements showed similar results. Source data are provided as a Source Data file.

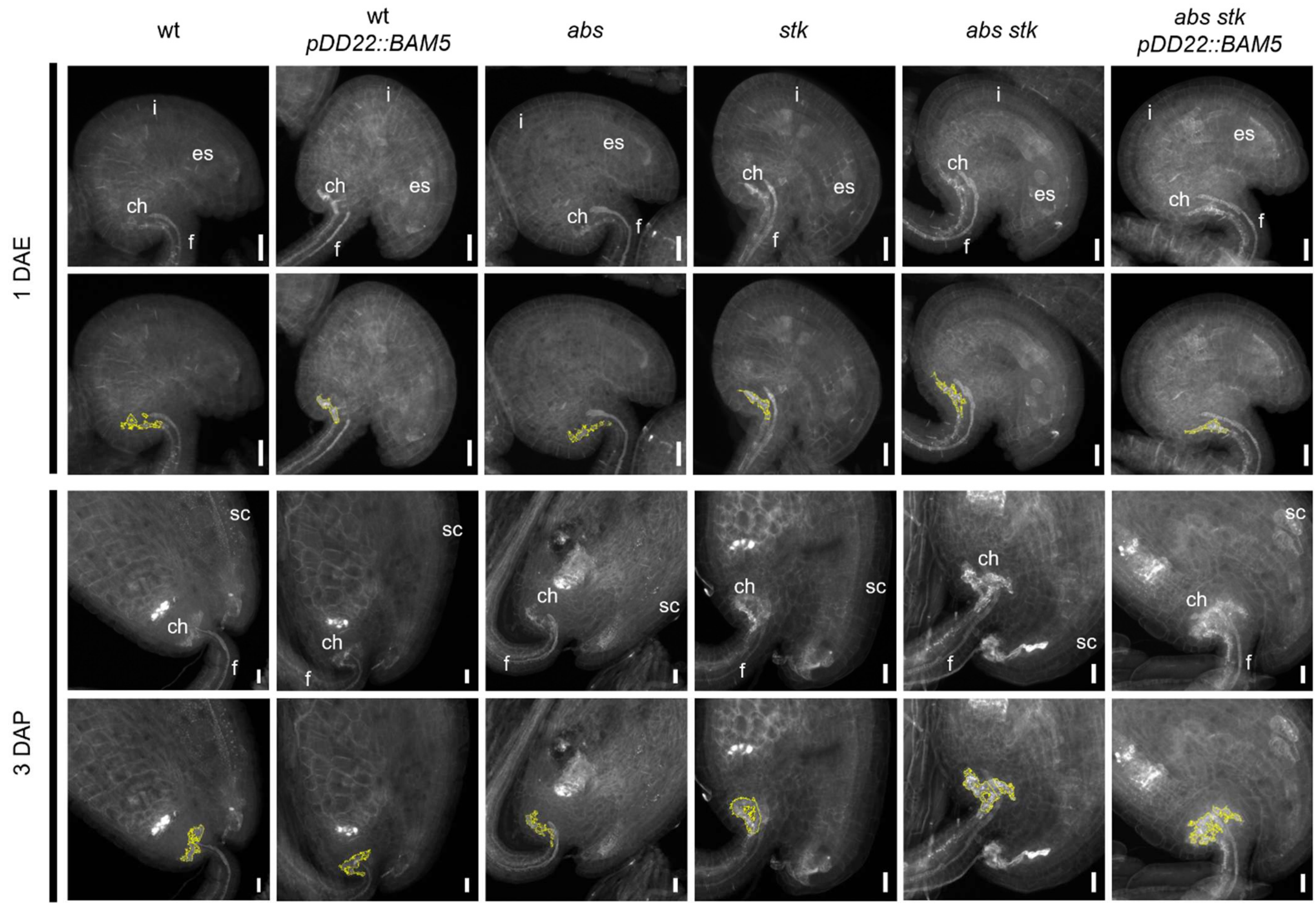

Supplementary Fig. 9. Representative confocal images of 1 DAE ovules and 3 DAP seeds used for callose quantification at the chalaza. Z-project of confocal images of ovules and seeds stained with aniline blue (white) that have been used for the quantification analysis in Fig. 7c (CTCF and area measurements). The area occupied by aniline blue signal (Region Of Interest: ROI) is highlighted in yellow. ch: chalaza, es: embryo sac, f: funiculus, i: integuments, sc: seed coat. Scale bars 20 µm.

a

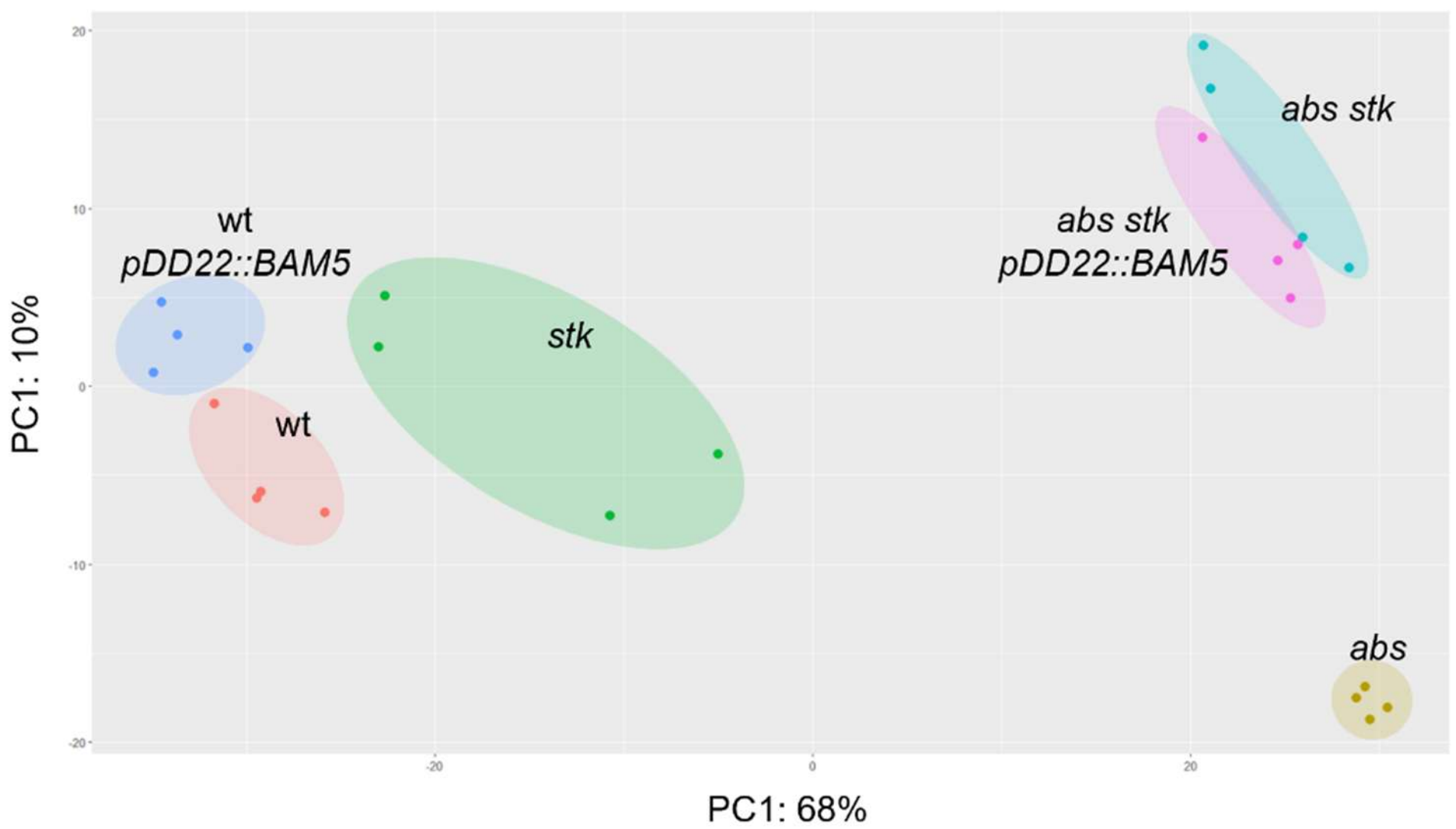

b

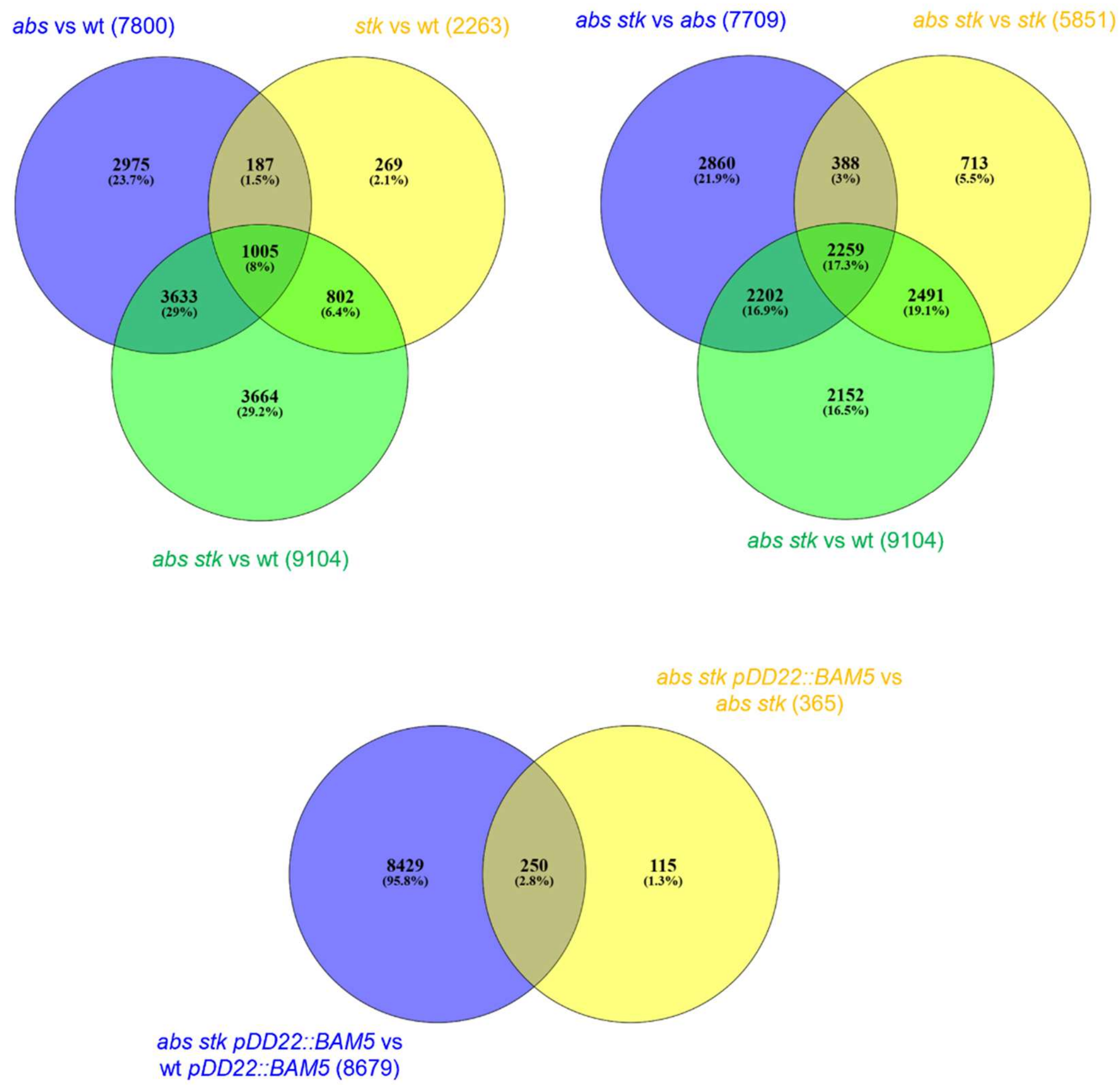

Supplementary Fig. 10. PCA and Venn Diagrams of RNA-seq data post-fertilization. a Principal Component Analysis (PCA) displaying the first two principal components (PC1, PC2) showing clustering of the four biological replicates of the RNA-seq samples at 3 DAP (*abs*, *stk*, abs *stk*, *abs stk pDD22::BAM5,* wt, wt *pDD22::BAM5*). n=4. **b** Venn Diagrams of the DEGs in mutant to wt comparisons and in the double to single mutants comparison and in the *abs stk pDD22::BAM5* comparisons. FDR ≤ 0.05 was considered to identify the DEGs. n=4.

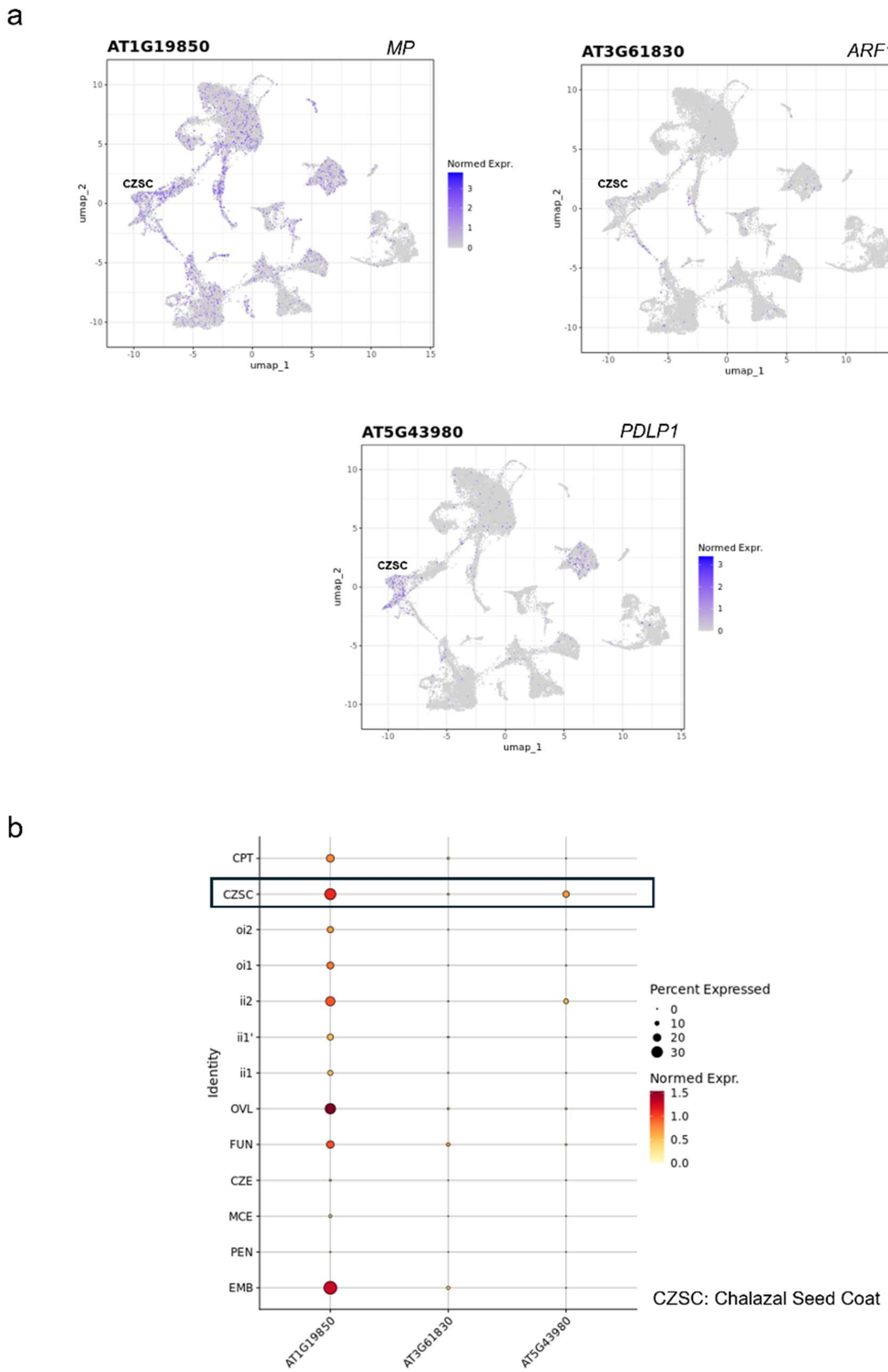

Supplementary Fig. 11: UMAP and Dotplot showing the expression pattern and level of *MP, ARF18* and *PDLP1* in snRNA-seq from Martin et al., 2026. a Uniform Manifold Approximation and Projection (UMAP) showing the expression of AT1G19850 (*MP*), AT3G61830 (*ARF18*) and AT5G43980 (*PDLP1*) in the seed at 3 DAP. **b** Dotplot showing the percentage of expression and the normalized expression of *MP, ARF18* and *PDLP1*.

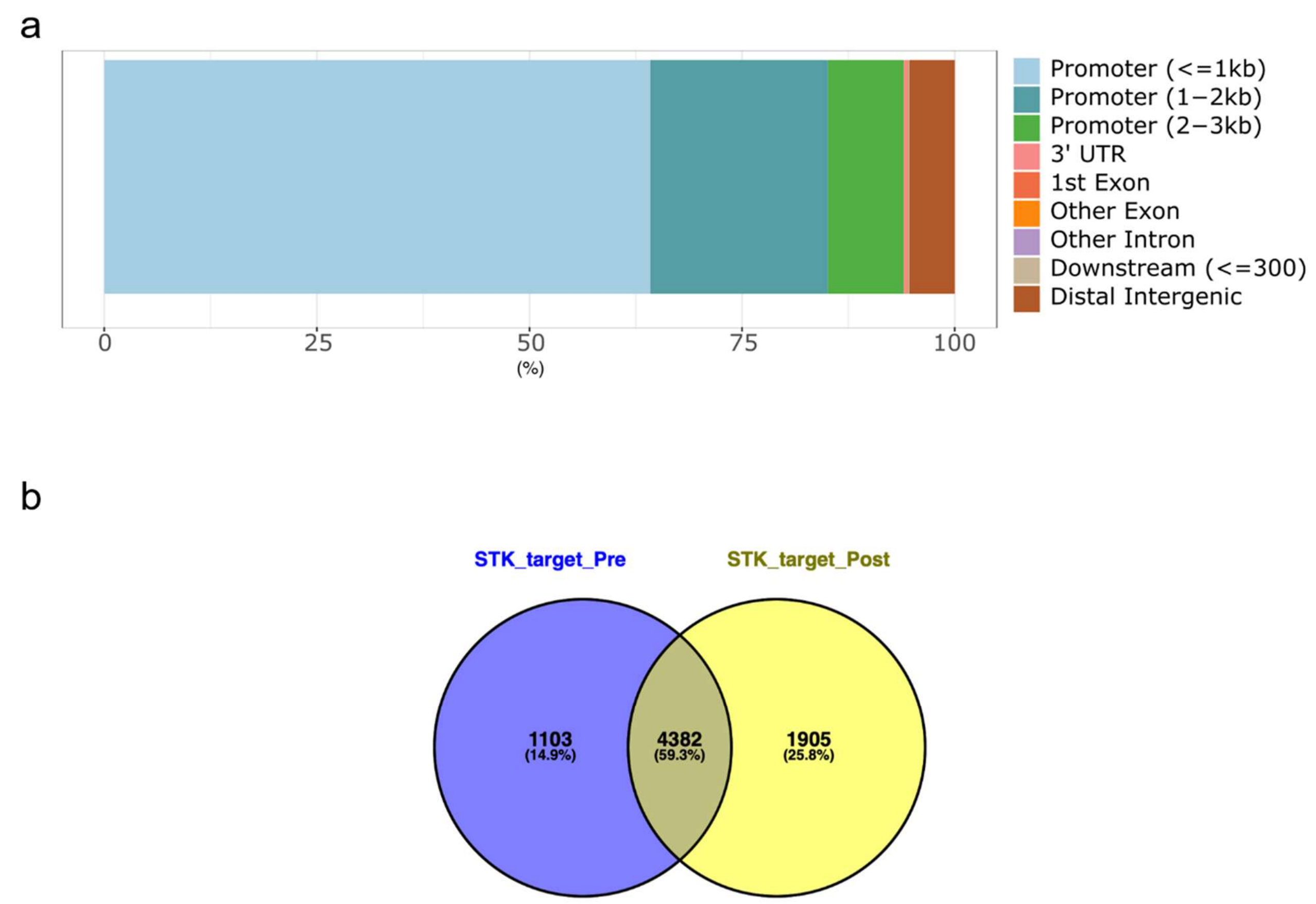

Supplementary Fig. 12. AnnoBar plot depicting the localization of the peaks in the STK ChIP-seq data post-fertilization and comparison between STK ChIP-seq in pre- and post-fertilization samples. a AnnoBar plot depicting the percentage distribution of ChIP-seq peaks across various genomic regions associated with STK target genes. **b** Venn Diagram of the intersection between STK targets pre- and post-fertilization.

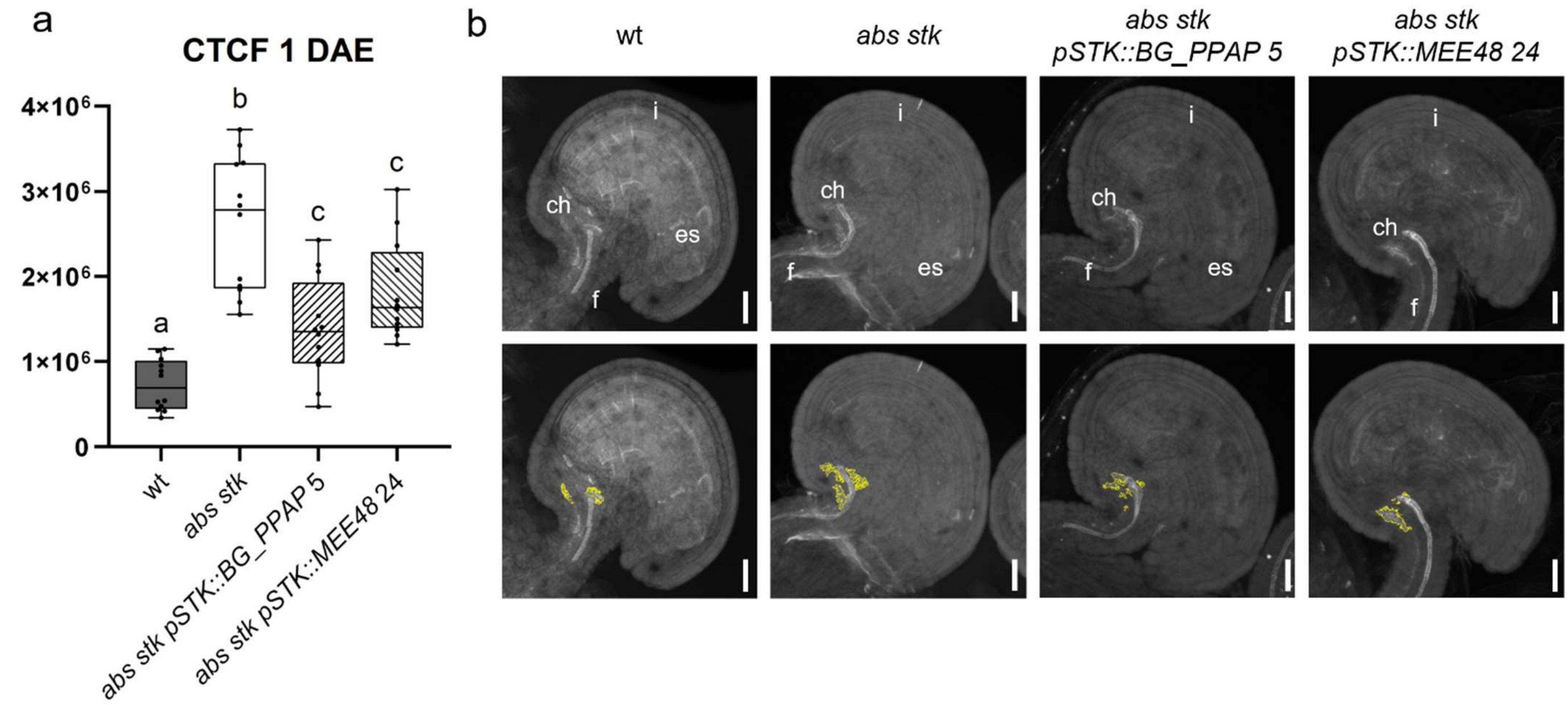

Supplementary Fig. 13. Aniline blue quantification in *pSTK::BG_PPAP* and *pSTK::MEE48* lines. a Box plots representing the Corrected Total Cell Fluorescence (CTCF) and the area of the aniline blue signal measured in the chalaza of 1 DAE ovules. Box plots elements correspond to: dots = single values; centre line = median; box limits = 25 and 75 percentile; whiskers = lowest and highest values. In the graph, different letters above the bars represent significant differences ($p < 0.05$) as determined by one-way ANOVA followed by Tukey's HSD, whereas the same letters indicate absence of a statistical difference between the respective genotypes. Three independent measurements showed similar results. Source data are provided as a Source Data file. **d.** Z-project of confocal images of ovules stained with aniline blue (white) that have been used for the quantification analysis in **a**. The area occupied by aniline blue signal (Region Of Interest: ROI) is highlighted in yellow. ch: chalaza, es: embryo sac, f: funiculus, i: integuments. Scale bar 20 µm.

Supplementary Table 1. Genes involved in starch and trehalose metabolism and sugar transport: comparison of ovule expression profiles from Zhang et al. (2023) with pre-fertilization RNA-seq and ChIP-seq datasets generated in this study. The table indicates: gene ID; gene name; whether each gene is predicted to be expressed in unfertilized ovule according to Zhang et al., 2023 transcriptomic data; whether each gene is differentially expressed, and the expression trend, in *abs stk* vs wt pre-fertilization RNA-seq data according to this study; whether each gene is STK target in pre-fertilization samples according to this study.

	**Gene ID**	**Gene name**	**Predicted expression in unfertilized ovules (Zhan et al., 2023)**	**DEG in RNAseq *abs stk* to wt pre-fertilization (this study)**	**STK target pre-fertilization (this study)**
**Starch**	AT4G24620	PGI/PGI1	yes	no	no
	AT5G51820	PGM/PGM1	yes	down	no
	AT5G48300	ADG1/APS1	yes	no	no
	AT1G05610	APS2	no	no	no
	AT5G19220	APL1/ADG2	yes	up	no
	AT1G27680	APL2	yes	down	no
	AT4G39210	APL3	yes	no	no
	AT2G21590	APL4	no	no	no
	AT1G32900	GBSS1/GBSS	yes	up	no
	AT5G24300	SS1/SSI	yes	up	no
	AT3G01180	SS2/SSII	yes	no	no
	AT1G11720	SS3/SSIII	yes	no	no
	AT4G18240	SS4/SSIV	yes	no	no
	AT3G20440	EMB2729/BE1	no	no	no
	AT5G03650	SBE2.2/BE2	yes	no	no
	AT2G36390	BE3	yes	no	yes
	AT2G39930	ISA1	yes	no	no
	AT1G03310	DBE1/ISA2	yes	up	no
	AT1G10760	SEX1/GWD1	yes	down	yes
	AT4G24450	PWD/GWD2	no	no	no
	AT5G26570	PWD/GWD3	yes	down	no
	AT3G52180	SEX4	yes	no	yes
	AT3G10940	LSF2	yes	no	no
	AT3G01510	LSF1	yes	no	no
	AT3G23920	BAM1	yes	up	no
	AT4G00490	BAM2	yes	up	no
	AT4G17090	CT-BMY/BAM3	yes	no	yes
	AT5G55700	BAM4	yes	down	yes
	AT4G15210	BAM5	yes	up	no
	AT2G32290	BAM6	no	no	no
	AT2G45880	BAM7	yes	no	no
	AT5G45300	BMY2/BAM8	yes	up	no
	AT5G18670	BMY3/BAM9	yes	down	yes
	AT4G25000	AMY1	yes	up	no
	AT1G76130	AMY2	yes	no	no
	AT1G69830	AMY3	yes	down	no
	AT4G09020	ISA3	yes	down	no
	AT5G04360	LDA/PU1	yes	down	no

	AT3G29320	PHS1	yes	down	no
	AT3G46970	PHS2	yes	no	yes
	AT5G64860	DPE1	yes	down	yes
	AT2G40840	DPE2	yes	up	yes
**Trehalose**	AT1G78580	TPS1	yes	no	yes
	AT1G16980	TPS2	no	no	no
	AT1G17000	TPS3	no	no	no
	AT4G27550	TPS4	no	no	no
	AT4G17770	TPS5	yes	up	no
	AT1G68020	ATTPS6/TPS6	yes	down	no
	AT1G06410	TPS7	yes	no	yes
	AT1G70290	TPS8	yes	down	yes
	AT1G23870	TPS9	yes	down	yes
	AT1G60140	TPS10	yes	down	no
	AT2G18700	TPS11	yes	down	yes
	AT1G22210	TPP1/TPPC	no	no	no
	AT1G35910	TPPD/TPP2	yes	no	yes
	AT1G78090	TPP3/TPPB	yes	no	yes
	AT2G22190	TPPE/TPP4	yes	up	no
	AT4G12430	TPPF/TPP5	no	no	no
	AT4G22590	TPP6/TPPG	yes	no	yes
	AT4G39770	TPP7/TPPH	no	no	yes
	AT5G10100	TPPI/TPP8	yes	up	yes
	AT5G51460	ATTPPA/TPP9	yes	no	yes
	AT5G65140	TPP10/TPPJ	yes	no	no
	AT4G24040	TRE1	yes	down	yes
**Sugar transporters**	AT5G17520	MEX1	yes	no	no
	AT5G16150	PGLCT/GLT1	yes	down	yes
	AT5G54800	GPT1	yes	no	yes
	AT1G61800	GPT2	yes	up	no
	AT1G21460	SWEET1	yes	no	yes
	AT3G14770	SWEET2	yes	up	yes
	AT5G53190	SWEET3	no	no	no
	AT3G28007	SWEET4	yes	no	yes
	AT5G62850	SWEET5 AtVEX1	no	no	no
	AT1G66770	SWEET6	no	no	no
	AT4G10850	SWEET7	yes	no	no
	AT5G40260	SWEET8	yes	no	no
	AT2G39060	SWEET9	no	no	no
	AT5G50790	SWEET10	yes	no	no
	AT3G48740	SWEET11	yes	no	no
	AT5G23660	SWEET12	yes	no	no
	AT5G50800	SWEET13	no	no	no
	AT4G25010	SWEET14	no	no	no
	AT5G13170	SWEET15	yes	no	yes
	AT3G16690	SWEET16	no	no	yes
	AT4G15920	SWEET17	no	no	yes
	AT1G71880	SUC1	yes	no	yes
	AT1G22710	SUC2	yes	up	no
	AT2G02860	SUC3	yes	no	no
	AT1G09960	SUC4	yes	no	yes

	AT1G71890	SUC5	yes	down	yes
	AT5G43610	SUC6	no	no	no
	AT1G66570	SUC7	no	no	no
	AT2G14670	SUC8	no	no	no
	AT5G06170	SUC9	no	no	no

Supplementary Table 2. Genes involved in callose metabolism and regulation and sugar transport: comparison of chalazal seed coat domain expression profiles from Martin et al. (2026) with post-fertilization RNA-seq and ChIP-seq datasets generated in this study. The table indicates: gene ID; gene name; whether each gene is predicted to be expressed in the chalazal seedcoat (CZSC) of 3 DAP seeds according to Martin et al., 2025 snRNA-seq data; whether each gene is differentially expressed, and the expression trend, in *abs stk* vs wt post-fertilization RNA-seq data according to this study; whether each gene is STK target in post-fertilization samples according to this study.

	**Gene ID**	**Gene name**	**Predicted expression in CZSC (Martin et al., 2025)**	**DEG in RNAseq *abs stk* to wt post-fertilization (this study)**	**STK target post-fertilization (this study)**
**Sugar transporters**	AT5G17520	MEX1	no	no	no
	AT5G16150	PGLCT/GLT1	no	no	no
	AT5G54800	GPT1	yes	no	yes
	AT1G61800	GPT2	no	up	no
	AT1G21460	SWEET1	no	no	yes
	AT3G14770	SWEET2	no	up	yes
	AT5G53190	SWEET3	no	no	yes
	AT3G28007	SWEET4	no	up	yes
	AT5G62850	SWEET5 AtVEX1	no	no	no
	AT1G66770	SWEET6	no	no	no
	AT4G10850	SWEET7	no	down	yes
	AT5G40260	SWEET8	no	no	no
	AT2G39060	SWEET9	no	no	no
	AT5G50790	SWEET10	yes	no	no
	AT3G48740	SWEET11	no	no	no
	AT5G23660	SWEET12	no	no	yes
	AT5G50800	SWEET13	no	no	no
	AT4G25010	SWEET14	no	no	no
	AT5G13170	SAG29/SWEET15	no	up	yes
	AT3G16690	SWEET16	no	no	no
	AT4G15920	SWEET17	no	no	yes
	AT1G71880	SUC1	no	no	yes
	AT1G22710	SUC2	no	no	no
	AT2G02860	SUT2/SUC3	no	up	yes
	AT1G09960	SUC4	no	no	yes
	AT1G71890	SUC5	no	up	yes
	AT5G43610	SUC6	no	no	no
	AT1G66570	SUC7	no	no	no
	AT2G14670	SUC8	no	no	no
	AT5G06170	SUC9	no	no	no
**Callose metabolism**	AT3G57270	BG1	no	down	no
	AT3G57260	BG2	no	no	no
	AT3G57240	BG3	no	down	no
	AT4G16260		no	no	no
	AT1G77780		no	no	no
	AT1G77790		no	no	no
	AT5G20340	BG5	no	no	no

	AT5G20330	BETAG4	no	no	yes
	AT1G33220		no	no	no
	AT5G20390		no	down	yes
	AT5G20560		no	no	no
	AT5G56590		no	no	no
	AT4G29360	AT4G29360	no	down	no
	AT4G26830		no	no	no
	AT5G55180	AT5G55180	no	no	yes
	AT2G05790		no	down	no
	AT2G01630		yes	down	no
	AT1G66250		no	no	yes
	AT1G11820		no	no	no
	AT3G13560	AT3G13560	no	down	no
	AT4G14080	MEE48	no	no	no
	AT3G23770		no	no	no
	AT3G07320	AT3G07320	no	no	no
	AT2G16230		no	no	no
	AT4G34480	AT4G34480	no	no	no
	AT5G42720	AT5G42720	yes	up	no
	AT3G15800	AT3G15800	no	no	no
	AT2G26600	AT2G26600	yes	no	no
	AT2G27500	AT2G27500	yes	no	yes
	AT1G32860	AT1G32860	yes	down	no
	AT5G42100	BG_PPAP	no	no	no
	AT4G18340	AT4G18340	no	no	no
	AT1G30080		yes	no	yes
	AT3G46570		no	no	no
	AT5G24318	AT5G24318	no	no	no
	AT3G55430		no	no	yes
	AT2G39640		no	no	no
	AT3G61410		no	no	no
	AT3G55780		no	no	no
	AT5G58090	AT5G58090	no	no	no
	AT4G31140	AT4G31140	no	no	yes
	AT5G20870	AT5G20870	no	no	no
	AT3G24330		no	no	no
	AT5G18220		no	no	no
	AT3G04010		no	no	yes
	AT1G64760	ZET	no	no	yes
	AT2G19440	ZETH	no	no	no
	AT5G64790		no	down	no
	AT5G58480		no	down	no
	AT4G04970	GSL1	yes	no	no
	AT2G13680	GSL2	no	no	no
	AT2G31960	GSL3	yes	no	no
	AT3G14570	GSL4	yes	no	yes
	AT4G03550	GSL5	no	no	yes
	AT1G05570	GSL6	no	no	yes
	AT1G06490	GSL7	no	no	no
	AT2G36850	GSL8	yes	no	no
	AT5G36870	GSL9	no	no	no

	AT3G07160	GSL10	yes	no	no
	AT3G59100	GSL11	yes	no	no
	AT5G13000	GSL12	yes	no	no
**ARFs**	AT1G59750	ARF1	no	no	yes
	AT5G62010	ARF2	no	no	no
	AT2G33860	ARF3	no	no	yes
	AT5G60450	ARF4	no	up	yes
	AT1G19850	MP/ARF5	yes	up	yes
	AT1G30330	ARF6	yes	no	yes
	AT5G20730	ARF7	yes	no	no
	AT5G37020	ARF8	yes	no	yes
	AT4G23980	ARF9	no	no	no
	AT2G28350	ARF10	no	no	yes
	AT2G46530	ARF11	no	up	yes
	AT1G34310	ARF12	no	no	no
	AT1G34170	ARF13	no	no	no
	AT1G35540	ARF14	no	no	no
	AT1G35520	ARF15	no	no	no
	AT4G30080	ARF16	no	up	yes
	AT1G77850	ARF17	no	no	no
	AT3G61830	ARF18	yes	up	no
	AT1G19220	ARF19	yes	no	yes
	AT1G35240	ARF20	no	no	no
	AT1G34410	ARF21	no	no	no
	AT1G34390	ARF22	no	no	no
	AT1G43950	ARF23	no	no	no
**PDLPs**	AT5G43980	PDLP1	yes	up	no
	AT1G04520	PDLP2	no	up	yes
	AT2G33330	PDLP3	no	no	no
	AT3G04370	PDLP4	no	no	no
	AT1G70690	PDLP5	no	no	no
	AT2G01660	PDLP6	no	no	yes
	AT5G37660	PDLP7	yes	no	no
	AT3G60720	PDLP8	yes	no	no

Supplementary Table 3. Probes and amplification hairpins used for HCR-RNA FISH.

**Transcript**	**Amplifier – Fluorescent dye**
BAM4 (AT5G55700)	X1 – Alexa Fluor 488
TRE1 (AT4G24040)	X3 – Alexa Fluor 546
GPT2 (AT1G61800)	X2 – Alexa Fluor 647
BG1 (AT1G52400)	X1 – Alexa Fluor 488
PDLP1 (AT5G43980)	X3 – Alexa Fluor 546
ARF5/MP (AT1G19850)	X2 – Alexa Fluor 647
