# Supplementary material for: ARABIDOPSIS Bsister and SEEDSTICK MADS-box transcription factors modulate maternal nutrient flow for seed development in Arabidopsis
Source: Nat Commun. 2026 Sep 10;17:10687. doi: 10.1038/s41467-026-77573-2 (PMC13655123; doi:10.1038/s41467-026-77573-2)
Supplement: Supplementary file 4 [file 41467_2026_77573_MOESM2_ESM.pdf]

Description of Additional Supplementary Files:

Supplementary Dataset 1: List of DEGs identified by RNA-seq at pre-fertilization stages and KEGG enrichment analysis.

Supplementary Dataset 2: List of STK target genes identified by STK ChIP-seq at pre-fertilization stages, KEGG and GO enrichment analysis.

Supplementary Dataset 3: Comparison between DEGs identified by RNA-seq and STK target genes identified by STK ChIP-seq at pre-fertilization stages.

Supplementary Dataset 4: Metabolomic analysis.

Supplementary Dataset 5: List of DEGs identified by RNA-seq at post-fertilization stages.

Supplementary Dataset 6: List of STK target genes identified by STK ChIP-seq at post-fertilization stages.

Supplementary Dataset 7: Comparison between STK target genes identified by STK ChIP-seq at pre-fertilization and post-fertilization stages.

Supplementary Dataset 8: Comparison between DEGs identified by RNA-seq and STK target genes identified by STK ChIP-seq at post-fertilization stages.

Supplementary Dataset 9: List of primer used in this study.
